# Fermented foods consumption, all-cause, and cause-specific mortality: a meta-analysis of prospective cohort studies

**DOI:** 10.3389/fnut.2026.1714437

**Published:** 2026-02-26

**Authors:** Antonia Matalas, Demosthenes Panagiotakos, Anthony Fardet, Isabelle Savary-Auzeloux, Christophe Chassard, Smilja Praćer, Guy Vergères, Diana Paveljšek

**Affiliations:** 1 Department of Nutrition – Dietetics, School of Health Sciences and Education, Harokopio University of Athens, Athens, Greece; 2 Unité de Nutrition Humaine (UNH), Institut national de recherche pour l'agriculture, l'alimentation et l'environnement (INRAE), Université Clermont Auvergne, Clermont-Ferrand, France; 3 Unité Mixte de Recherche sur le Fromage (UMRF 0545), Institut national de recherche pour l'agriculture, l'alimentation et l'environnement (INRAE), VetAgro Sup, Université Clermont Auvergne, Aurillac, France; 4 Institute for Biological Research Siniša Stanković, National Institute of the Republic of Serbia, University of Belgrade, Belgrade, Serbia; 5 Research Division Microbial Food Systems, Agroscope, Bern, Switzerland; 6 University of Ljubljana, Biotechnical Faculty, Ljubljana, Slovenia

**Keywords:** all-cause mortality, cancer, cardiovascular disease, cohort studies, fermented foods

## Abstract

**Purpose:**

Fermented foods are widely consumed, contribute important bioactive compounds and microbial metabolites to the diet, and play an important role in global nutrition. This meta-analysis evaluated evidence from prospective cohort studies on the association between fermented food and non-alcoholic beverage consumption and the risk of all-cause and cause-specific mortality in healthy adults.

**Methods:**

A systematic search of three databases identified 50 cohort studies, including more than three million participants, examining associations between fermented food intake and all-cause, cardiovascular disease (CVD), and cancer mortality. Risk of bias was assessed using the Newcastle-Ottawa Scale. Random-effects meta-analyses using the DerSimonian and Laird method, were conducted on fully adjusted risk estimates comparing highest vs. lowest intake categories.

**Results:**

Higher consumption of chocolate, cheese, and fermented milks (including yogurt) was associated with lower all-cause and CVD mortality. Fermented milk consumption also showed a protective association with cancer mortality. Miso and bread consumption showed no significant associations with mortality.

**Conclusion:**

This is the first meta-analysis to comprehensively evaluate the association between fermented food intake and mortality. Findings support a protective role for specific fermented foods consumption, i.e., milks, cheese, and chocolate, against all-cause and CVD mortality, with additional evidence of a protective effect of fermented milk on overall cancer mortality. These associations may underline the role of bioactive peptides, polyphenols, and microbial metabolites that modulate the gut microbiota, improve vascular function, and reduce the risk of certain chronic diseases.

**Systematic review registration:**

https://osf.io/vg7f6, identifier: vg7f6.

## 1 Introduction

Diet quality, including degree of food processing and nutritional composition, is an important determinant of morbidity and mortality risk (1, 2). Fermentation is one of the oldest food processing methods and remains central to human diets worldwide. Beyond improving food preservation and safety, it generates bioactive compounds and microbial metabolites that may exert functional effects on health, thereby linking fermented foods with long-term disease prevention and mortality risk.

Fermented products made from cereals, legumes, vegetables, fruits, nuts, and milk are integral to human diets and contribute significantly to food safety and sustainability. Estimates on the contribution of fermented foods to dietary intakes are generally missing. A recent study in Switzerland concluded that fermented foods and beverages (including alcoholic beverages) account for as much as 27% of daily energy intake, while they also contribute more than 30% of daily intake of calcium, phosphorus, sodium, zinc, vitamins A and B_12_ (3).

Fermented foods are defined as foods produced by desired microbial growth and enzymatic conversion of food raw material components; thus, many of these foods contain microbes and their metabolites, including bioactive peptides, polyphenols and vitamins (4). Due to their diverse composition and influence on the gut microbiota, fermented foods are often considered functional foods, which justifies the growing scientific and public health interest in their relationship with long-term health outcomes.

Nowadays, the popularity of fermented foods is increasing largely due to growing awareness of their potential health benefits. In particular, in view of the emerging scientific evidence on the need for dietary recommendations for a daily intake of microbes (5), traditional fermented foods present a practical and culturally trusted means for obtaining diverse and abundant populations of microbes. Despite the remarkable variability across the various types of fermented foods and the lack of product standardization, positive health outcomes have been observed for several of these products (6). The potential health benefits of the microbial content and the characteristic active components of non-alcoholic fermented foods have been demonstrated by *in vitro* as well as animal studies. Furthermore, controlled human interventions have investigated the effects of specific fermented foods on chronic disease risk factors, immune system function, and other aspects of human health (7). Nevertheless, the interpretation of these findings is subject to an inherent difficulty stemming from the variations observed in commercial fermented foods used in clinical studies.

The impact of consumption of individual fermented foods on all-cause, cardiovascular disease (CVD) and/or overall cancer mortality has been previously evaluated by the meta-analyses. These studies, in their vast majority, dealt with dairy products. Some of the cohort studies included in these meta-analyses aimed at assessing the health impact of one particular kind of fermented food, while others examined broader aspects of diet, such as the impact of full- vs. low-fat dairy products. Regarding yogurt, the latest two metanalyses (8, 9) concluded that higher consumption is associated with both lower all-cause and CVD mortality, but not with cancer mortality (8), while earlier analyses did not detect associations between higher levels of consumption and mortality risks (10–12). For cancer mortality risk, Lu et al. (13) as well as and Jin and Je (14) detected no associations with consumption of yogurt and other types of fermented milks in the entire population of study participants, the latter study however reported a lower mortality risk in women. Regarding cheese, Mazidi et al. (12) concluded that cheese consumption is inversely associated with all-cause mortality but not with CVD mortality, and an updated analysis of previous meta-analyses by Zhang et al. (15) detected an inverse association with both all-cause and CVD mortality. Previously, Guo et al. (11) and Tong et al. (16) had reported neutral relationships with both all-cause and cancer mortality. Focusing on bread intake, Gaesser et al. (17) found no associations with total or site-specific cancer mortality, while Kwok et al. (18), examining consumption of non-white bread in the context of various dietary parameters, analyzed two cohort studies and reported an inverse association with all-cause mortality.

Fermented foods comprise a diverse group of products produced from a wide range of substrates and microbial cultures, and global estimates suggest that more than 5,000 varieties are consumed as staple foods, delicacies, or beverages across different cultural contexts (19). Despite this diversity in raw materials, microbial consortia, and processing traditions, many fermented foods share several defining biological characteristics. They often contain live microorganisms at the time of consumption or retain fermentation-derived metabolites, such as organic acids, peptides, phenolic compounds, and other microbially transformed constituents, that can modulate the gut microbiota and influence host metabolic and immune pathways (4, 20). The ability of fermented foods to interact with the gut microbial ecosystem represents a unifying attribute, extending even to products in which fermentation-derived metabolites and polyphenol-rich compounds exert biological activity independent of viable microbial counts. These shared features constitute a common mechanistic dimension relevant to health outcomes (21). The links between the consumption of fermented foods and mortality, have already been discussed in the context of EFSA's requirements for health claims and were correlated in a narrative manner as part of the PIMENTO project (22, 23). To discern associations between fermented food consumption and mortality risk, we also performed a meta-analysis, in which, for the first time, as many individual fermented foods as the available evidence permits are examined together, hence, illuminating a gap of the existing literature. Considering the central role fermented foods play in eating habits of populations across the world, this meta-analysis highlights the potential benefits of habitual fermented food consumption, particularly in relation to chronic disease prevention.

## 2 Methods and materials

The Meta-Analyses of Observational Studies in Epidemiology (MOOSE) (24) and Preferred Reporting Items for Systemic Reviews and Meta-Analyses (PRISMA) 2020 guidelines (25) were followed. A pre-defined protocol (26), based on Cochrane principles (27), was developed.

### 2.1 Data search

A systematic literature search was performed independently between January 2023 and March 2023 in accordance with study protocol (26). Electronic scientific databases, namely PubMed, Scopus, and Cochrane were searched for human observational studies since 1972 on the association between the consumption of fermented foods and mortality risk. The search strategy included keywords related to: (I) fermented food items (i.e., dairy products, meat and fish, vegetables, fruit and nuts, cereals and legumes); (II) mortality outcomes (death, mortality rate, mortality risk); and (III) observational studies (i.e. cohort study, prospective study, longitudinal study). The query syntax of searching is shown in Supplementary Table S1 in the Supplementary Material 1. In addition, the reference lists of relevant systematic reviews and meta-analyses obtained with this search string were hand-searched to identify any missing prospective studies. A second search was conducted prior to submission of the paper, which included studies published since the initial search in 2023 and up to 31 March 2025. The sensitivity of the systematic search was also verified by back referencing the collected systematic reviews, meta-analyses, and independent studies.

### 2.2 Study selection

A hierarchical approach, i.e., screening of the titles and the abstracts followed by the full-text screening of each publication, was used to search for relevant studies using the CADIMA tool (28). Inclusion and exclusion criteria were in accordance with the PIO framework (population, intervention, outcome) (29). A consistency test was applied to minimize bias in the evaluation among reviewers. The study selection process was conducted through an independent assessment by two reviewers for each publication in two stages: the title/abstract screening, followed by the full-text screening, while any discrepancies were resolved through discussions, and when disagreements persisted, a third reviewer was consulted.

### 2.3 Selection criteria

Eligible populations were healthy adults, thus studies involving individuals with diagnosed pathological conditions (e.g. diabetes, hypertension), food poisoning, pregnant women, or infants, were excluded. Prospective cohort studies who had specifically assess the consumption of one or more fermented foods as part of daily diet, were enrolled. Studies on probiotics, bioactive compounds, fermented alcoholic beverages as well as coffee, as they examine effects of caffeine rather than coffee in its entirety, were excluded. Outcomes of interest were all-cause mortality and cause-specific mortality, expressed in terms such as “death,” “mortality rate,” “mortality risk,” or “risk of fatal outcomes.” If multiple publications included the same study or cohort with identical exposures and outcomes, only the most recent publication was retained.

**Figure 1:**
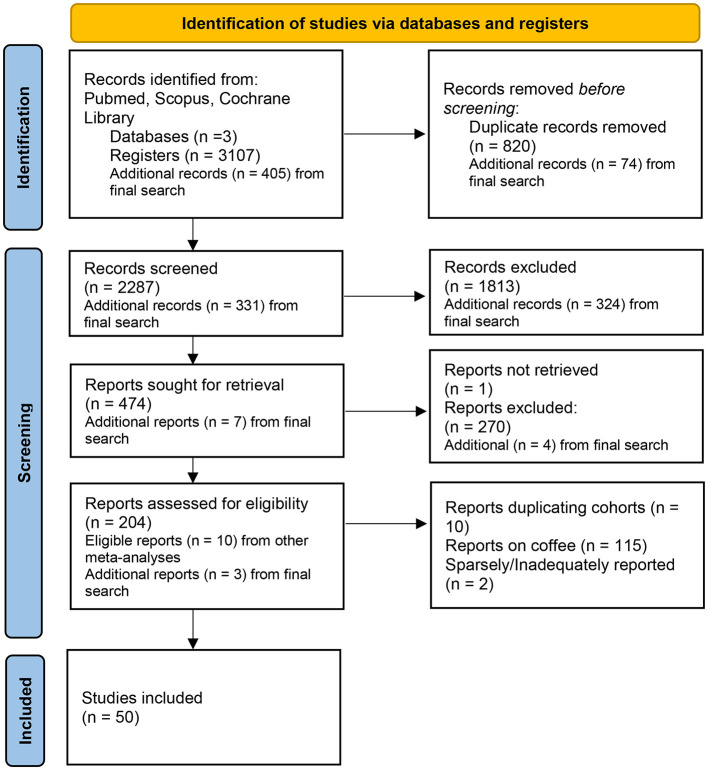
PRISMA flow-chart of the studies' selection process.

All applied inclusion criteria, along with the additional verification of the retained studies, were independently reviewed and confirmed by two additional reviewers. The study selection process is shown on Figure 1. The completed PRISMA checklist is provided in Supplementary Material 2, and details of excluded papers with reasons for exclusion at full-text level are available in Supplementary Material 3.

### 2.4 Data screening, extraction, and quality assessment

Four authors independently reviewed the abstracts for adherence to selection criteria. Any disagreements were addressed with the help of a fifth author. Full texts of selected titles and abstracts were retrieved and reviewed independently for eligibility and completeness. Inter-rater agreement was within the substantial agreement range, i.e., Cohen's kappa = 0.9 (30). Key variables manually extracted from the selected studies included country or countries of the study, total number of participants and events (deaths), type(s) of fermented food assessed and their exposure levels, length of study's follow-up, as well as age and sex of the participants in each study (Supplementary Material 4). Quality and risk of bias for each study were independently assessed by two authors using the Newcastle-Ottawa Scale (NOS) which classifies cohort studies against a nine-point scale based on three criteria: representativeness of the participants selected, comparability of cohorts, and assessment of the outcome (31).

### 2.5 Statistical analysis

Meta-analysis was performed using STATA statistical software (StataCorp. Stata: Release 17. Statistical Software. College Station, TX: StataCorp LLC). Combined estimates from fully adjusted models are reported for the association between the lowest vs. highest categories of fermented foods consumption and the investigated outcomes, which is in line with previous research (25). A log-transformation of the retrieved effect size measures (hazards ratios or relative risks) was first performed. Then, the Residual Maximum Likelihood (REML) random-effects model was used (32). The selection of the random effects model was decided based on the observed heterogeneity level. For studies reporting relative risks, it was assumed that they approximate hazard ratios (HR), considering both as risk ratios (RRs) (33). To calculate the standard error from the reported confidence interval of RRs, the lower and upper limits were converted to the log scale, because ratio measures are symmetric only after log transformation, and then performed the relevant calculations. All combined estimates are presented in forest plots as log-RRs with their 95% confidence intervals (CI). Reverse log-transformation to combined RRs was performed, to the combined log-transformed effect size measures, for better interpretation of the results. If the number of cases in each category was missing, these data were inferred based on the number of total cases and the reported effect size. To evaluate the robustness of the combined estimates, sensitivity analysis was undertaken by eliminating one-study-at-a-time approach to assess the effect of the study's omission on the combined pooled RRs and 95% CIs. Heterogeneity across studies was examined by the Cochran Q, *I*^2^ statistic. *I*^2^ threshold value of 25, 50, and 75% indicate low, moderate, and high heterogeneity, respectively. The source of heterogeneity was investigated using stratified meta-analysis, as per study's years followed up, total number of participants and cases, key confounders controlled, and study's quality. In addition, sensitivity meta-regression testing was applied examining for a significant change in the pooled effect estimates. Publication bias was evaluated visually using a contour-enhanced funnel plot, as well as the Begg adjusted rank correlation test, and the Egger regression asymmetry test (34–36). The trim-and-fill method was also used to detect and adjust the results for any potential publication bias by reporting the probable impact of missing studies on the combined estimates (37). For site-specific cancer outcomes or outcomes with a small number of contributing studies, pooled risk estimates are reported in the text only.

## 3 Results

### 3.1 Study characteristics

Details of the systematic literature search are illustrated in Figure 1. In total, 50 different studies were selected for this meta-analysis examining the risk of various fermented foods on all-cause mortality, as well as on CVD, and cancer specific mortality. Details about the studies are presented in Table 1. In studies where the authors reported results separately for males and females, the data were analyzed and reported accordingly.

**Table 1:** Characteristics of prospective studies included in this meta-analysis on fermented food consumption and all-cause and cause-specific mortality.

**Study reference**	**Fermented food(s) assessed causes of death**	**Cohort—country**	**Start year**	**Population**	**Parti-cipants**	**Total deaths**	**Years FU**	**Adjustments**	**Quality grade**
Bongard et al. (38)	Fermented milks, cheese, bread All causes	Monitoring trends and determinants in CVD (MONICA)—France	1995	Male 45–64 years	960	150	14.8	Age, study-center, income tax, obesity, alcohol consumption, smoking, PA, presence of chronic condition, diet quality score	8
Bonthuis et al. (39)	Fermented milks, cheese All causes, CVD	Nambour Skin Cancer Study—Australia	1992	Male and female, 25–78 years	1,529	177	14.4	Age, sex, BMI, PA, alcohol consumption, school leaving age, pack-years smoking, dietary supplement use, beta-carotene treatment during trial, presence of medical condition, EI, Ca intake	8
Buijsse et al. (75)	Chocolate CVD	Zutphen Elderly Study—Netherlands	1985	Male, 65–84 years	470	152	15	Age, BMI, alcohol intake, PA, smoking, aspirin and anticoagulant use, physician in BP, diet prescription, EI, dietary cholesterol; intake of trans FAs, saturated fat, folic acid, vit C, vit E, β-carotene, K, Na, Ca and Mg	9
Chow et al. (82)	Bread Lung CA	Lutheran Brotherhood Cohort—USA	1966	Male, ≥35 years	17,633	219	20	Age, smoking, industry/occupation	8
Dehghan et al. (40)	Fermented milks, cheese All causes	Prospective Urban Rural Epidemiology (PURE)—Multiple	2003	Male and female, 35–70 years	136,384	6,796	9.1	Age, sex, study center, education, urban vs. rural, smoking, PA, history of diabetes, family history CVD and CA, quintiles of fruit, vegetable, red meat, and starchy-foods intake, EI	8
Ding et al. (65)	Cheese All causes, CVD, CA	Nurses' Health Study—USA	1976	Females, 30–55 years	74,805	25,182	40	Age, BMI, PA, alcohol consumption, family history of CVD and CA, hypertension and hypercholesterolemia status, healthy eating score, EI, smoking, postmenopausal status and hormone use	8
Ding et al. (65)	Cheese All causes, CVD, CA	Health Professionals Study—USA	1986	Males, 40–75	49,602	23,560	30	Age, BMI, PA, alcohol consumption, family history of CVD and CA, hypertension and hypercholesterolemia status, healthy eating score, EI, smoking,	8
Ding et al. (65)	Cheese All causes, CVD, CA	Nurses' Health Study II—USA	1989	Females, 25–42 years	93,348	2,696	27	Age, BMI, PA, family history of CVD and CA, hypertension and hypercholesterolemia status, healthy eating score, EI, smoking, alcohol intake, postmenopausal status and hormone use	8
Farvid et al. (41)	Fermented milks, cheese All causes, CVD, CA	Golestan Study—Iran	2004	Males and females, 36–85 years	42,403	3,291	8	Age, ethnicity, education, marital status, residency, wealth score, BMI, smoking, opium use, alcohol use, systolic BP, PA, family history CA, medication use, EI	8
Fortes et al. (66)	Cheese, bread All causes	Rome Elderly Cohort—Italy	1993	Males and females, ≥65 years	161	53	5	None	7
Ge et al. (42)	Fermented milks, cheese All causes, CVD, CA	JPHC—Japan	1995	Males and females, 40–69 years	93,310	23,758	19.3	Age, study area, BMI, smoking, alcohol use, PA, hypertension medication, history diabetes, green tea and coffee use, EI, EI-adjusted vegetable and fruit intake, fat intake, dairy intake menopausal status and hormone use,	7
Goldbohm et al. (43)	Fermented milks, cheese All causes, CVD	NLCS—Netherlands	1986	Males and females, 55–69 years	120,852	16,136	10	Age, education, smoking, BMI, PA, multivitamin use, alcohol, EI-adjusted mono- and polyunsaturated FAs, vegetable and fruit consumption	8
Guo et al. (44)	Fermented milks, unspecified dairy, cheese All causes	MONICA—Denmark	1982	Males and females, 30–60 years	1,746	660	30	Sex, BMI, alcohol consumption, education, smoking, PA, family history of MI, multivitamin use, serum cholesterol and triacylglycerols, incidence of hypertension, EI	8
Hirayama (79)	Miso GI CA	Hirayama Cohort—Japan	1966	Males and females, ≥ 49 years	265,118	3,913	13	Sex, age, smoking, occupation, residence, marital status, alcohol consumption; rice, meat, fish, milk, pickles, green-yellow vegetables and hot green tea intake	6
Ho et al. (76)	Chocolate CVD	Million Veteran Program—USA	2018	Male and females, 64 ± 12 years	188,447	6,946	3.2	Age, sex, race, BMI, smoking, PA, alcohol consumption	5
Katagiri et al. (78)	Miso CVD	Japan Public Health Center-Based Prospective Study (JPHC) —Japan	1990	Males and females, 45–74 years	92,915	13,303	14.8	Age, geographical area, smoking, frequency of alcohol intake, BMI, PA, history of diabetes, antihypertensives, health check-up, EI, intake of green tea, coffee, fish, meat, fruit and vegetables	8
Khan et al. (57)	Fermented milks, cheese, bread, miso CA, Lung and GI CA	Hokkaido Study—Japan	1984	Male and female, 40–97 years	3,158	244	13.8	Age, health status, health education, health screening, smoking	7
Kojima et al. (59)	Fermented milks, cheese GI CA	Japan Collaborative Cohort Study (JACC)—Japan	1988	Male and females, 40–79	107,824	457	9.9	Age, family history of colorectal cancer, BMI, frequency of alcohol intake, current smoking status, walking time per day, educational level	8
Kurozawa et al. (80)	Miso GI CA	Japan Collaborative Cohort Study (JACC)—Japan	1988	Male and females, 40–59 years	110,792	401	9.9	None	5
Kwok et al. (77)	Chocolate CVD	European Prospective Investigation into Cancer and Nutrition (EPIC)-Norfolk—UK	1993	Males and females, 40–79 years	20,951	1,107	11.3	Sex, age, smoking, BMI, PA, EI, alcohol consumption, diabetes, systolic BP, LDL serum total and HDL cholesterol	8
Lin et al. (45)	Fermented milks all causes, CVD, CA	National Health and Nutrition Examination Survey (NHANES)—USA	1999	Males and females, >18 years	32,625	3,881	8.1	Age, sex, race, BMI, white blood cell count, hemoglobin, platelet count, total bilirubin, creatinine, blood urea nitrogen, history of hypertension, diabetes, asthma, congestive heart failure, CHD, stroke, chronic bronchitis, and CA	9
Lu et al. (46)	Fermented milks, cheese All causes, CVD, CA	Miyagi Cohort—Japan	1990	Males and females, 40–64 years	34,161	6,876	25	Age, education, BMI, smoking, alcohol drinking status, history of hypertension and diabetes, EI, fish, vegetable and fruit intake	9
Mann et al. (67)	Cheese All causes, CVD	Oxford Vegetarian Study (OVS)—UK	1980	Males and females, 16–79 years	10,802	383	13.3	Age, sex, smoking, social class	6
Matsumoto et al. (58)	Ferments milks CA	Jichi Medical School (JMS)—Japan	1992	Males and females, 19–93 years	11,606	255	9.2	Age, sex	5
Mazidi et al. (12)	Fermented milks, cheese All causes, CVD, CA	National Health and Nutrition Examination Survey (NHANES)—USA	1999	Males and females, >20 years	24,474	3,520	6.4	Age, sex, BMI, race, education, marital status, poverty to income ratio, physical activity, smoking, alcohol consumption, history of hypertension and diabetes, EI, intake of carbohydrate, sat. fat, protein, and dietary fiber	9
Mills et al. (70)	Cheese reproductive CA	Agricultural Health Study (AHS)—USA	1960	Females, 30–85 years	16,190	142	20	Age at menarche, age at first pregnancy, age at menopause, percent desirable weight, education, consumption of other animal products	8
Miyagawa et al. (47)	Fermented milks all causes, CVD, CA	Japan Multi-Institutional Collaborative Cohort Study (J-MICC)—Japan	2005	Males and females, 35–69	79,715	3,723	12	Age, sex, study site, history of cardiometabolic diseases, BMI, smoking status, drinking status, physical activity, dietary intake of red meat, fish, vegetables, fruits	9
Nakanishi et al. (48)	Fermented milks all causes, CVD, CA	Yamagata Study—Japan	2009	Male and females, 40–74 years	14,264	265	9	Age, sex, BMI, education, smoking, alcohol consumption, BMI, history of hypertension, and diabetes	7
Ngoan et al. (81)	Miso GI CA	Fukuoka Prefecture Study—Japan	1986	Males and females, 15–96 years	13,250	116	13	Age, sex	5
Ozasa et al. (61)	Fermented milks, cheese Lung CA	Japan Collaborative Cohort Study (JACC)—Japan	1988	Males and females, 40–79 years	98,248	572	7.7	Age, sex, parents' history of lung CA, smoking status, smoking index	8
Paganini-Hill et al. (71)	Chocolate All causes	Leisure World Cohort—USA	1980	Males and females, 44–101 years	13,624	11,386	23	Age, sex, smoking, exercise, BMI, alcohol intake, history of hypertension, angina, heart attack, stroke, diabetes, rheumatoid arthritis, CA	7
Pala et al. (49)	Fermented milks, cheese All causes, CVD, CA	European Prospective Investigation into Cancer and Nutrition (EPIC)—Italy	1993	Males and females, 45–64 years	45,009	2,468	14.9	Age, region, sex, weight, height, waist-to-hip ratio, alcohol consumption, smoking, PA, relative index of inequality, Italian Med/mean index, EI, intake of sugar	8
Park et al. (62)	Fermented milks, cheese reproductive CA	NIH-AARP Diet and Health Study—USA	1995	Males, 50–71 years	293,888	178	6	Age, ethnicity, education, marital status, BMI, PA, smoking, alcohol consumption, history of diabetes, family history of prostate CA, PSA screening, EI, intake of Ca	7
Praagman et al. (50)	Fermented milks, cheese All causes, CVD, CA	European Prospective Investigation into Cancer and Nutrition-NL—Netherlands	1993	Male and females, 20–70 years	34,409	2,436	15	Age, sex, smoking, BMI, PA, education, hypertension, alcohol consumption, EI, E-adjusted intake of fruit and vegetable	8
Praagman et al. (55)	Unspecified dairy, cheese CVD	Rotterdam Study—Netherlands	1990	Males and females, ≥55 years	42,359	532	17.3	Age, sex, BMI, smoking, education alcohol consumption, EI, intake of vegetables, fruit, meat, bread, fish coffee and tea	8
Rebello et al. (83)	Bread CVD	Singapore Chinese Health Study—Singapore	1993	Male and females, 45–74 years	53,469	1,660	15	Age, sex, BMI, dialect group, interview year, smoking, alcohol consumption, PA, education, history of hypertension, HRT, EI, ratio of PUFAs to SFAs, intake of cholesterol and fiber	9
Sakauchi et al. (63)	Fermented milks, cheese reproductive CA	Japan Collaborative Cohort Study (JACC)—Japan	1988	Females, 40–79 years	63,541	77	13.3	Age, BMI, PA, education, menopausal status, number of pregnancies, history of sex hormone use	7
Schmid et al. (51)	Fermented milks all causes, CVD, CA	Nurses' Health Study (NHS) and 1986 Health Professionals—USA	1980	Males and females, 30–59 years	122,626	33,228	32	Sex, Height, BMI, BMI at age 18/21, race, PA, smoking, alcohol consumption, history of hypertension, hypercholesterolemia and diabetes, family history of CA, diabetes, and MI, multivitamin and aspirin use, menopausal status and hormone use, EI, glycemic load, intake of meat, nuts, fruits, vegetables, Ca and fiber	8
Silva et al. (86)	Unspecified dairy CVD	Brazilian Longitudinal Study of Adult Health (ELSA)—Brazil	2008	Males and females, 35–74 years	6,671	42	8	Age, sex, BMI, educational level, PA, smoking, alcohol consumption, diabetes, hypertension and hypercholesterolemia status	8
Sluik et al. (52)	Fermented milks all causes	European Prospective Investigation into Cancer and Nutrition (EPIC)—Multiple countries	1992	Males and females, 45–64 years	258,911	12,135	9.9	Age, sex, region, education, alcohol consumption, PA, smoking, factor loadings for first three dietary patterns derived via factor analysis	8
Soedamah-Muthu et al. (85)	Unspecified dairy All causes	Whitehall II—UK	1997	Males and females, 35–55 years	4,526	237	11.7	Age, ethnicity, employment grade, smoking, BMI, alcohol intake, physical activity, family history of CHD/hypertension; fruit and vegetable, bread, meat, fish, coffee and tea intake	7
Sonestedt et al. (53)	Fermented milks, cheese All causes	Malmö Diet and Cancer Study (MDCS)—Sweden	1991	Males and females, 45–73 years	26,190	7,156	19	Age, sex, diet assessment method, season, BMI, education, physical activity, smoking, alcohol habits, EI, diet (fruit and vegetables, meat, fiber, sugar-sweetened beverages)	9
Sun et al. (72)	Chocolate All causes, CVD	Women's Health Initiative (WHI)—USA	1993	Females, 50–79 years	84,709	25,388	19	Age, BMI, race, ethnicity, education, family income, neighborhood SES, smoking, PA, unopposed estrogen use, estrogen and progesterone use, alcohol, coffee and tea intake, EI, diabetes status, serum cholesterol, family history of heart attack or stroke, Healthy Eating Score	8
Sonestedt et al. (53)	Fermented milks, cheese All causes	Malmö Diet and Cancer Study (MDCS)—Sweden	1991	Males and females, 45–73 years	26,190	7,156	19	Age, sex, diet assessment method, season, BMI, education, physical activity, smoking, alcohol habits, EI, diet (fruit and vegetables, meat, fiber, sugar-sweetened beverages)	9
Sun et al. (72)	Chocolate All causes, CVD	Women's Health Initiative (WHI)—USA	1993	Females, 50–79 years	84,709	25,388	19	Age, BMI, race, ethnicity, education, family income, neighborhood SES, smoking, PA, unopposed estrogen use, estrogen and progesterone use, alcohol, coffee and tea intake, EI, diabetes status, serum cholesterol, family history of heart attack or stroke, Healthy Eating Score	8
Tognon et al. (54)	Fermented milks, cheese All causes	Northern Sweden Health and Disease Study (NSHDS)—Sweden	1986	Males and females, 24–74 years	103,256	6,892	13.7	Age, sex, BMI, screening year, smoking, education, EI	8
Tognon et al. (64)	Cheese All causes	Gothenburg H70 Birth cohort study—Sweden	1971	Male and females, 70 years	1,213	833	13.2	Sex, BMI, birth cohort, smoking, education, marital status, PA, EI	9
Tokui et al. (60)	Fermented milks cheese, miso GI CA	Japan Collaborative Cohort Study (JACC)—Japan	1988	Males and females, 40–79 years	110,792	859	9.9	Age, sex	6
van Aerde et al. (68)	Unspecified dairy, cheese All causes, CVD	Hoorn Study—Netherlands	1989	Male and females, 50–75 years	1,956	403	12.4	Age, sex, BMI, smoking, education, alcohol consumption, PA, EI, intake of meat, fish, bread, vegetables, fruit, coffee and tea	8
Virtanen et al. (69)	Unspecified dairy, cheese All causes	Kuopio Ischaemic Heart Disease Risk Factor Study (KIHD)—Finland	1984	Males, 42–60 years	2,641	1,225	22.3	Age, BMI, examination year, income, education, marital status, PA, smoking, alcohol consumption, diagnosis of T2 diabetes, CVD and CA, hypertension, use of cardiac, hypercholesterolemia, hypertension, or diabetes medications, EI, intake of fiber and saturated, MUFA, PUFA and trans FA	8
Wada et al. (84)	Bread CVD	Takayama Study—Japan	1993	Male and females, >35 years	29,079	1,686	14.1	Sex, smoking status, physical activity, alcohol intake, coffee intake, salt Intake, marital status, education level, BMI, history of diabetes and hypertension, menopausal status	8
Zhang et al. (56)	Fermented milks CVD	Malmö Diet and Cancer Study—Sweden	1991	Males and females, 57.8 years (mean age)	20,499	2,531	21	Age, sex, dietary assessment version, season, leisure-time physical activity, smoking status, alcohol consumption, education, heredity score (including cancer, myocardial infarction, stroke, diabetes), EI, diet quality index	9
Zhao et al. (73)	Chocolate All causes, CVD	Alpha-Tocopherol, Beta-Carotene Cancer Prevention study (ATBC) —Finland	1986	Males, 50–69 years	27,111	22,064	31	Age, BMI, PA, smoking, serum HDL and total cholesterol, intervention assignment, education, alcohol consumption, alternate MedDiet score, systolic and diastolic BP, history of CVD and diabetes, EI	8
Zhong et al. (74)	Chocolate All causes, CVD	Prostate, Lung, Colorectal and Ovarian Cancer study—USA	1993	Males and females, 55–74 years	91,891	19,586	13.5	Age, sex, ethnicity, BMI, PA, smoking, education, marital status, study center, history of hypertension, and diabetes, aspirin use, hormone use, alcohol consumption, EI, consumption of red meat, processed meat, fruit, vegetable, whole grain, dairy, coffee and tea	8

BMI, body mass index; BP, blood pressure; CA, cancer (all types); CHD, coronary heart disease; CVD, cardiovascular disease; FA, fatty acids; EI, energy intake; GI CA, gastrointestinal cancer; HRT, hormone replacement therapy; MI, myocardial infarction; MUFA, monounsaturated fatty acids; PA, physical activity; PUFA, polyunsaturated fatty acids; SES, socioeconomic status.

### 3.2 Fermented milk and yogurt consumption, all-cause and cause-specific mortality

**Figure 2:**
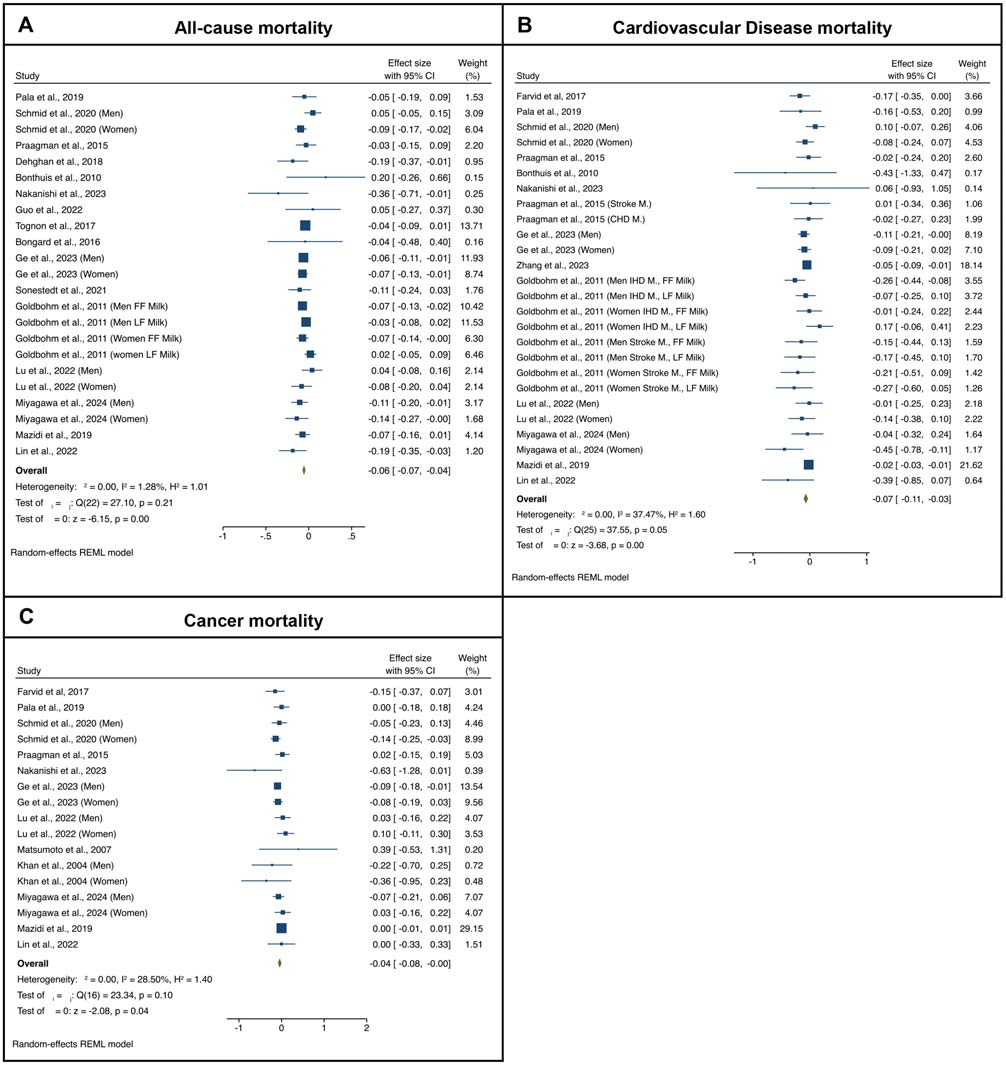
Forest-plots illustrating the association between fermented milk consumption (log-RR of highest quartile to the lowest quartile) and **(A)** all-cause, **(B)** cardiovascular disease (CVD), and **(C)** cancer mortality. Results are presented as pooled log-transformed Risk Ratios and their corresponding 95% confidence intervals.

With respect to fermented milk consumption and all-cause mortality, the meta-analysis includes a total of 1,172,824 participants and 133,548 deaths (12, 38–54). The forest plots from the meta-analyses examining the association between the highest vs. lowest categories of fermented milks consumption and all-cause mortality risk are presented in Figure 2. As it can be seen, higher fermented milk consumption levels were associated with lower risk of all-cause mortality as compared to the lowest consumption levels (pooled RR = 0.941, *p* < 0.001). The exclusion of any single study (i.e., leave-one-out sensitivity analysis) did not change the pooled effect estimate (pooled effect estimates varied between 0.93 and 0.95). No significant publication bias was detected (Egger test *z* = −0.20, *p* = 0.840; Begg test *z* = −0.33, *p* = 0.778). The start year of each study was associated with the pooled effect estimate; specifically, entering newer studies in the meta-analysis seem to reduce the strength of the pooled estimate (*p* = 0.007). None of the rest tested factors showed any significance in influencing the observed pooled effect [i.e., study's duration (*p* = 0.772), and number of participants (*p* = 0.077)].

Regarding CVD outcomes (12, 39, 41–43, 45–51, 55, 56), higher fermented milk consumption levels were associated with lower risk of CVD death as compared to the lowest consumption category (pooled RR = 0.932, *p* < 0.001), but with a considerable level of heterogeneity (*I*^2^ = 37.47%). The exclusion of any single study did not change the pooled effect estimate which varied between 0.91 and 0.93. No significant publication bias was detected (Egger test *z* = −2.51, *p* = 0.012; Begg test *z* = 0.97, *p* = 0.331). None of the tested potential mediators revealed significance in influencing the observed pooled effect [i.e., study's duration (*p* = 0.491), start year (*p* = 0.1541), and number of participants (*p* = 0.060)].

Higher fermented milk consumption levels were also associated with lower overall cancer mortality risk, as compared to the lowest consumption category (pooled RR = 0.940, *p* = 0.04) (12, 41, 42, 45–51, 57, 58). The level of heterogeneity was moderate (*I*^2^ = 28.50%). The exclusion of any single study did not change the pooled RRs which varied between 0.93 and 0.96. No significant publication bias was detected (Egger test *z* = −0.94, *p* = 0.345; Begg test *z* = 0.75, *p* = 0.455). None of the tested potential mediators influenced the observed pooled effect based on the sensitivity analysis [i.e., study's duration (*p* = 0.681), start year (*p* = 0.981), and number of participants (*p* = 0.543)].

In addition, yogurt consumption was examined separately, and a meta-analysis was conducted including only studies that assessed mortality risk in relation to yogurt intake, without considering other types of fermented milk. As the respective database is a subset of the aforementioned yogurt and fermented milks database, the results on the association between yogurt consumption only and mortality risks are presented in Supplementary Figure S1. Overall, combined estimates revealed that higher yogurt consumption was significantly associated with a lower risk of all-cause mortality (pooled RR = 0.933, *p* < 0.001; low heterogeneity) (12, 39–41, 44–52). A weak inverse association that did not reach statistical significance was observed for CVD mortality (pooled RR = 0.951, *p* = 0.06; low heterogeneity) (12, 39, 41, 45–51, 55). No significant association was detected between yogurt consumption and all-cancer mortality (pooled RR = 0.971, *p* = 0.22; low heterogeneity) (12, 41, 45–51, 57, 58) as well as gastrointestinal cancer (pooled RR = 0.923, *p* = 0.45; low heterogeneity) (57–60) and lung cancer mortality (pooled RR = 0.878, *p* = 0.41; low heterogeneity) (57, 58, 61). Reproductive cancer mortality (62, 63) was not meta-analyzed due to the limited available information.

### 3.3 Cheese consumption, all-cause and cause-specific mortality

**Figure 3:**
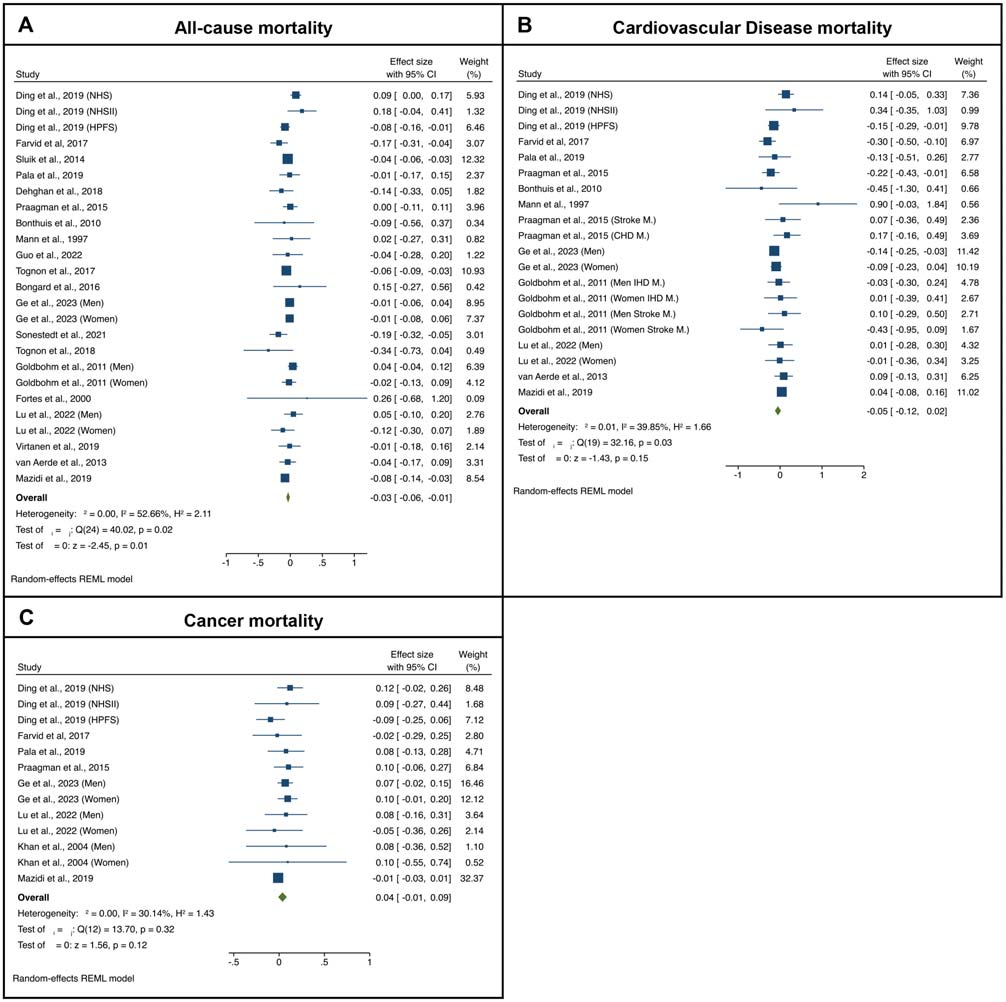
Forest-plots illustrating the association between cheese consumption (highest quartile to the lowest quartile) and **(A)** all-cause, **(B)** cardiovascular disease (CVD), and **(C)** cancer mortality. Results are presented as pooled log-transformed Risk Ratios and their corresponding 95% confidence intervals.

With respect to fermented cheese consumption and all-cause mortality, the meta-analysis includes a total of 1,158,122 participants and 146,786 deaths from 20 studies (12, 38–44, 46, 49, 50, 52–54, 64–69). In Figure 3 the forest plots from the meta-analysis examining the association between the highest vs. lowest categories of consumption and all-cause mortality, as well as cause-specific mortality are presented. Overall combined estimates revealed that higher consumption levels were significantly associated with lower risk of death from any-cause as compared to the lowest consumption levels (pooled RR = 0.970, *p* = 0.01). The moderate *I*^2^, i.e., 52.66%, suggests substantial heterogeneity among studies. The exclusion of any single study (i.e., leave-one-out analysis) did not change the pooled effect estimate (i.e., pooled RRs varied between 0.95 and 0.98). No significant publication bias was detected (Egger test *z* = 0.34, *p* = 0.734; Begg test *z* = 0.19, *p* = 0.851). None of the tested potential mediators revealed any significance in influencing the observed pooled effect through the meta-regression analysis that applied, i.e., study's duration (*p* = 0.438), start year (*p* = 0.332), number of participants (*p* = 0.655).

Regarding CVD (12, 39, 41–43, 46, 49, 50, 55, 65, 67, 68), the meta-analysis revealed that cheese consumption was not associated with CVD mortality risk (*p* = 0.150; Figure 3). The exclusion of any single study did not alter the pooled RRs which varied between 0.86 and 1.04. Additionally, no significant publication bias was detected (Egger test *z* = 1.54, *p* = 0.122; Begg test *z* = 1.14, *p* = 0.155). None of the tested potential mediators revealed was significantly associated with the pooled effect in the meta-regression analysis, i.e., study's duration (*p* = 0.642), start year (*p* = 0.215), and number of participants (*p* = 0.510).

With respect to overall cancer mortality (12, 41, 42, 46, 49, 50, 57, 65), the meta-analysis revealed that cheese consumption was not associated with risk of death as compared to the lowest consumption category (Figure 3). The exclusion of any single study did not alter the pooled effect estimate (pooled RRs varied between 1.05 and 1.08). No significant publication bias was detected (Egger test *z* = −0.35, *p* = 0.728; Begg test *z* = −1.85, *p* = 0.086). None of the tested potential mediators influenced the observed pooled effect based on the meta-regression analysis, i.e., study's duration (*p* = 0.379), start year (*p* = 0.521), and number of participants (*p* = 0.315). However, when focusing on cancer specific mortality, a significant association was observed regarding lung cancer [pooled RR = 0.657, 95% CI (0.417, 0.897)] (57, 61); no significant association was observed regarding gastrointestinal cancers mortality [pooled RR = 1.07, 95% CI (0.791, 1.348)] (57, 59, 60), and reproductive cancers mortality (62, 63, 70), for which no quantitative synthesis was performed due to limited available information.

### 3.4 Chocolate consumption, all-cause and cause-specific mortality

**Figure 4:**
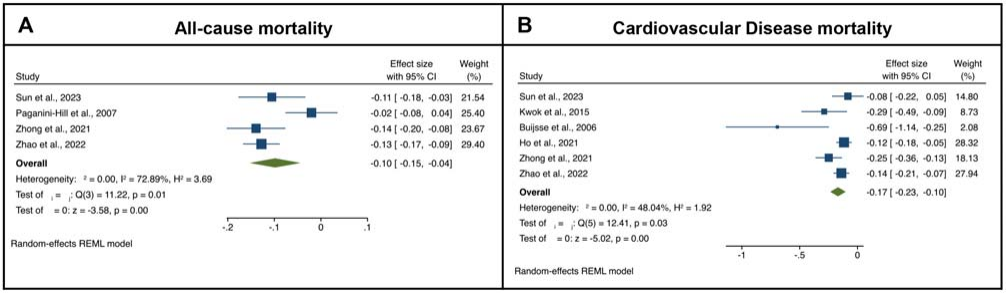
Forest-plots illustrating the association between chocolate products consumption (highest quartile to the lowest quartile) with **(A)** all-cause and **(B)** cardiovascular disease (CVD) mortality. Results are presented as pooled log-transformed Risk Ratios and their corresponding 95% confidence intervals.

Considering chocolate consumption and mortality, the meta-analysis includes four studies (71–74). Higher chocolate consumption levels were associated with lower risk of all-cause mortality as compared to the lowest consumption levels (pooled RR = 0.901, *p* < 0.001), but with a high level of heterogeneity, i.e., *I*^2^ = 72.89% (Figure 4). The exclusion of any single study did not change the pooled effect estimate (pooled RRs varied between 0.88 and 0.92). No significant publication bias was detected (Egger test *z* = 0.41, *p* = 0.678; Begg test *z* = 0.72, *p* = 0.470). Meta-regression analysis revealed that the start year of each study significantly mediated the evaluated association (*p* = 0.025), i.e., more recent cohorts showing weaker effect estimates, whereas none of the other tested mediators had any significance in influencing the observed pooled effect, i.e., study's duration (*p* = 0.772), and number of participants (*p* = 0.098).

Regarding CVD outcomes (72–77), the meta-analysis revealed that higher chocolate consumption levels were associated with lower risk of CVD death as compared to the lowest consumption (pooled RR = 0.843, *p* < 0.001); the level of heterogeneity was moderate, i.e., 48.04% (Figure 4). The exclusion of any single study did not change the pooled effect estimate (i.e., pooled RRs varied between 0.81 and 0.86). In addition, significant publication bias was observed (Egger test *z* = −2.66, *p* = 0.0079; Begg test *z* = −1.72, *p* = 0.180). None of the tested potential mediators revealed significance in influencing the observed pooled effect [i.e., study's duration (*p* = 0.117), start year (*p* = 0.317), and number of participants (*p* = 0.555)].

No studies were retrieved evaluating overall or cancer-specific mortality and chocolate consumption to allow for performing a meta-analysis.

### 3.5 Miso consumption, all-cause and cause-specific mortality

Regarding miso consumption and all-cause mortality, the only study available showed significant protective effect in females but not in males, the dataset combining both sexes reaching significance [RR = 0.922, 95% CI (0.863, 0.980)] (78). Cause-specific mortality showed no significant association as regards CVD outcomes [RR = 0.940, 95% CI (0.840, 1.061)], all cancer mortality [pooled RR = 0.859, 95% CI (0.603, 1.114)], and gastrointestinal cancer mortality [pooled RR = 0.695, 95% CI (0.384, 1.007)] (57, 60, 78–81).

### 3.6 Bread consumption, all-cause and cause-specific mortality

Concerning bread consumption and all-cause mortality, only two studies with only 1,121 participants and 203 deaths in total were found in the literature. No significant association between all-cause mortality with bread consumption was observed [pooled RR = 0.756, 95% CI (0.441, 1.071)] (38, 66). Similarly, no significant association was found regarding CVD [pooled RR = 0.885, 95% CI (0.733, 1.036)], and lung cancer mortality [pooled RR = 0.817, 95% CI (0.419, 1.215)] (57, 82–84).

### 3.7 Unspecified fermented dairy products consumption, all-cause and cause-specific mortality

The association between unspecified fermented dairy products consumption and mortality was evaluated in four studies of a total of 10,869 participants and 2,525 deaths (44, 68, 69, 85). No significant associations were observed with all-cause or CVD mortality (44, 50, 55, 68, 69, 85, 86). The exclusion of any single study did not change the pooled RR estimates which varied between 0.88 and 1.00. No significant publication bias was detected (Egger test *z* = −0.68, *p* = 0.497; Begg test *z* = −1.02, *p* = 0.734). None of the tested potential mediators revealed significance in influencing the observed pooled effect during the meta-regression analysis [i.e., study's duration (*p* = 0.999), start year (*p* = 0.999), and number of participants (*p* = 0.999)]. The retrieved data on unspecified fermented dairy did not suffice for an evaluation of the association with overall or cancer-specific mortality.

## 4 Discussion

In this meta-analysis of more than three million participants from 50 prospective cohort studies, higher intakes of fermented milk products (including yogurt), cheese, and chocolate were associated with lower risks of all-cause and CVD mortality, with additional evidence supporting an inverse association between fermented milk intake and total cancer mortality. In contrast, evidence for other fermented foods such as miso, fermented bread, and certain soy-based products was insufficient.

Notably, this is the first meta-analysis, to our knowledge, to assess the relationship between chocolate consumption and all-cause mortality risk. This broad evidence base enabled a comparative assessment of several distinct fermented food categories within a unified analytical framework. While previous meta-analyses have typically focused on a single food or a narrow group of fermented foods, our work adopts a broader perspective, integrating a wide range of products in one analysis. Given the global prevalence of fermented foods and their cultural, nutritional, and functional diversity, these findings contribute important evidence to ongoing discussions about dietary patterns and chronic disease prevention. Although the available epidemiological data are still limited for several fermented foods, mortality and cause-specific mortality can be considered global indicators of long-term health and integrative markers of diet-related risk.

A coherent epidemiologic rationale for examining multiple fermented food categories arises from several shared biological characteristics. As detailed in our previous systematic review (22), fermentation leads to the formation of organic acids, bioactive peptides, phenolic metabolites, vitamins, and microbially modified substrates that may influence inflammation, oxidative stress, blood pressure, glycemic control, lipid metabolism, and immune function. Many fermented foods also contain viable microorganisms at the time of consumption, and even those with low or no residual viability can deliver fermentation-derived compounds that interact with the gut microbial ecosystem. These mechanistic pathways provide biological plausibility for the associations observed in prospective cohort studies and justify evaluating different fermented food categories within a unified analytical framework, while still analyzing each food type separately to account for heterogeneity.

Mechanisms that may be responsible for the slight, but significant, protective effect may relate to the presence of beneficial compounds as described previously, *e.g*., probiotic bacteria and bioactive peptides with antioxidant anti-hypertensive, and/or immunological properties, or improved protein digestion (20, 87). A large cross-sectional study among older U.S. adults which showed that higher estimated dietary intake of live microbes was linked to a 26% reduced risk of all-cause mortality and a 37% reduced risk of cardiovascular mortality (88) reinforces this notion.

Fermented foods constitute a highly heterogeneous category shaped by both the raw materials used and the microbial consortia driving fermentation. Our earlier systematic review noted that differences in substrates and in processing practices, including pasteurization, roasting, or the use of specific starter cultures, substantially influence microbial viability and the profile of fermentation-derived metabolites at the time of consumption (22). These compositional differences may modulate the generation and bioavailability of bioactive peptides, polyphenols, vitamins, and other fermentation-dependent compounds, which in turn contribute to the observed variability in epidemiological associations across food types. Recognizing this complexity underscores the importance of improving exposure characterization in future cohort studies, particularly with regard to raw material origin, microbial content, and processing conditions.

### 4.1 Fermented milks consumption, all-cause and cause-specific mortality

Fermented milks, including yogurt, are widely consumed across many regions around the world. They have been associated with various potential health benefits due to their nutritional value and probiotic content; however, findings from prospective studies on their association with mortality risk vary. The present meta-analysis revealed that consumption of fermented milks (i.e., yogurt and other types, such as soured milk) was associated with lower all-cause-, lower CVD-, as well as lower overall cancer- mortality risk. These findings are aligned with the findings by most of the previous analyses, albeit not with all of them. Regarding all-cause mortality, a previous meta-analysis of seven cohort studies indicated a *U*-shaped association with fermented milk consumption, albeit, with a substantial heterogeneity (i.e., *I*^2^ = 88%) (89). In another meta-analysis which focused on cancer mortality, fermented milk consumption was found to be associated with 15% lower cancer mortality risk in females, but not in males (14). Also, regarding yogurt consumption specifically, a recent meta-analysis (8) reported that higher intakes of yogurt were significantly associated with 7% lower risk of deaths from all causes (*I*^2^ = 47.3 %) and 11% lower risk for CVD (*I*^2^ = 33.2 %), but not with cancer [RR 0.96; 95 % CI: (0.89, 1.03), *I*^2^ = 26.5 %). Moreover, a dose-response relationship was revealed in this meta-analysis; each additional serving of yogurt consumption per day was significantly associated with a 7% reduced risk of all-cause (*I*^2^ = 63.3%) and 14% reduced risk of CVD mortality (*I*^2^ = 36.6%) (6). In contrast, two other previous meta-analyses showed that yogurt consumption was not associated with all-cause mortality risk (11, 90). Additionally, one metanalysis that examined yogurt consumption in relation to cancer mortality did not find any associations (13).

### 4.2 Cheese consumption, all-cause and cause-specific mortality

Cheese, a product of milk fermentation, represents a significant source of nutrients such as calcium, protein, and bioactive peptides for many people. Although some studies have suggested health benefits, particularly in relation to cardiometabolic outcomes, the overall evidence remains inconclusive, and no consistent association has been observed between cheese consumption and all-cause or cause-specific mortality. In the present analysis, higher consumption of cheese was inversely associated with all-cause as well as CVD mortality; on the other hand, results for its association with cancer mortality are mixed: a higher risk emerged for overall cancer, a lower risk for lung cancer and no relationship with gastrointestinal cancer mortality. Our results are generally in line with those of a recent analysis of previous meta-analyses which showed that cheese consumption was associated with 5% lower risk for all-cause mortality and 7% lower risk for CVD, but not associated with cancer mortality (15). The earlier studies and meta-analyses regarding cheese consumption tend to show neutral effect toward all-cause mortality risk (11, 13, 14, 16), while, one of them (12) that also examined colorectal cancer, reported that higher cheese consumption was related to 22% higher colorectal cancer mortality.

### 4.3 Chocolate consumption, all-cause and cause-specific mortality

Chocolate, derived from fermented cacao beans, is a popular snack consumed across diverse populations globally. While it contains bioactive compounds with potential health benefits, current evidence remains inconclusive, and no clear or consistent association has been established between chocolate consumption and major health outcomes, including mortality (91). An inverse association between increasing levels of chocolate consumption and risk of death from any cause, as well as from CVD, was detected by the present analysis, while, due to lack of studies, associations with cancer mortality could not be evaluated. In line with our findings, a meta-analysis which assessed risk of CVD and CVD mortality simultaneously using 19 prospective studies four of which reported death outcomes (73), found an inverse association with chocolate consumption. Consumption of cocoa and chocolate has been associated with beneficial effects on CVD risk factors, such as flow-mediated dilatation at 90–150 min, decreased blood pressure, as well as enhanced insulin sensitivity (92). These benefits have been mainly attributed to cocoa's flavanols, which become more bioaccessible through fermentation and microbial metabolism.

### 4.4 Miso consumption, all-cause and cause-specific mortality

Miso is traditional Asian seasoning produced through fermentation that is commonly consumed in East Asian regions. It is rich in bioactive components; however, despite its cultural and dietary significance, current evidence does not demonstrate a clear or consistent association between miso consumption and mortality or other major health outcomes. Based on the reports of 11 cohort studies examining stomach, colorectal, or hepatic cancer in Japan, we did not confirm any association between miso consumption and mortality from gastrointestinal cancers. As mentioned, miso is made from soybeans with the addition of salt and rice molds (koji) and, thus, because of its high salt content, it has been linked to increased rates of gastric cancer (93). High concentrations of sodium in the diet have been reported to enhance the carcinogenicity of N-nitroso compounds and of *Helicobacter pylori* infection and thus, to weaken the protective effect of the mucous barrier (94). However, in line with our results, a meta-analysis of 21 cohort studies in Japan, found no association between miso consumption and gastrointestinal cancer risk (95).

### 4.5 Bread consumption, all-cause and cause-specific mortality

Bread is one of the most widely consumed staple foods globally, typically produced through fermentation using bakers' yeast or sourdough cultures. Despite its ubiquity and fermented nature, the health effects of bread consumption remain inconclusive, with current evidence showing no consistent association with mortality or major health outcomes. Results from the present meta-analysis indicate neutral associations of bread consumption with both all-cause and cause-specific mortality and agree with the sole previous meta-analysis which reported null association with cancer mortality (17).

Beyond the epidemiological associations identified in this meta-analysis, the biological plausibility of the health effects of fermented foods is increasingly supported by mechanistic insights. To illustrate this perspective, Table 2 summarizes the major groups of fermented foods investigated here, the representative bioactive compounds identified in the underlying studies, and the proposed mechanisms of action relevant to human health. These pathways, including modulation of the gut microbiota, regulation of vascular and metabolic processes, and antioxidant and anti-inflammatory effects, provide the context for interpreting the associations with mortality risk observed in this meta-analysis.

**Table 2:** Overview of the major groups of widely consumed non-alcoholic fermented foods analyzed in this meta-analysis, representative bioactive compounds reported in the included studies, and proposed mechanisms of action that may explain their observed associations with mortality.

**Fermented food group**	**Key bioactive compounds**	**Mechanisms of action**	**Relevant prospective studies**
Fermented dairy products (fermented milk, cheese)	Bioactive peptides, SCFA, minerals (Ca, P, K), vitamin K2, CLA, lactic acid bacteria	ACE inhibition and blood pressure regulation, lipid modulation, anti-inflammatory effects, improved gut microbiota composition and metabolite production, enhanced calcium bioavailability, vitamin K2-related vascular and metabolic effects, potential cancer-preventive effects	(38, 41–43, 46, 48, 53, 54, 59, 85, 86)
Fermented soy products (miso)	Isoflavones (including aglycones), soy proteins, dietary fiber, PUFA, bioactive peptides	Blood pressure reduction, antioxidant and anti-inflammatory effects, cardiometabolic protection	(60, 78)
Fermented grains (Bread)	Resistant starch, peptides, B-vitamins, minerals (esp. iron, zinc), polyphenols, organic acids; microbial phytase	Improved digestibility, lower glycaemic response, enhanced mineral absorption via phytate degradation; sourdough fermentation improves iron bioavailability	(38, 84)
Cocoa and chocolate	Flavanols (epicatechin, catechin, procyanidins), theobromine	Vascular health via nitric oxide pathway, anti-inflammatory and antioxidant effects, platelet and lipid oxidation modulation	(72, 74–76)

ACE, angiotensin-converting enzyme; SCFA, short-chain fatty acids; Ca, calcium; P, phosphorus; K, potassium; CLA, conjugated linoleic acid; PUFA, polyunsaturated fatty acids.

## 5 Limitations

Our analysis concerns fermented foods and all-cause, as well as cause-specific mortality. While previous meta-analyses have typically focused on a single food or a narrow group of fermented foods, our work adopts a broader perspective, integrating a wide range of products in one analysis. Nonetheless, certain limitations should be acknowledged. The type, preparation method and serving size of fermented foods vary widely, making standardization and comparison across populations particularly challenging. In addition, exposure assessment relied largely on self-reported dietary questionnaires, which are prone to measurement error and may attenuate true associations. The studies selected in the present meta-analysis are observational, prospective cohorts, thus, susceptible to confounding factors such as overall diet quality, recall biases of over/under reporting, and lifestyle factors. Although most cohorts adjusted for major confounders, residual confounding cannot be excluded. Most selected studies did not account for competing risks, thereby altering the observed probability of the event. In the synthesis of the retrieved data we assumed that the hazard ratios and relative risks were approximately risk ratios; this is a formal and common procedure (33), but, it should be acknowledged that although hazard ratios and relative risks may be similar when the proportional hazards assumption holds and event rates are low, but they can differ substantially when hazards vary over time or when the outcome is common. Although the results refer to an effect of fermented food consumption on mortality, this should not be interpreted causally; the reported association reflects only a statistical metric. Substantial heterogeneity was observed in some studies, reflecting meaningful differences in fermentation practices, raw materials and consumption contexts. Fermented dairy, chocolate and soy products differ considerably in microbial starter cultures, salt or fat content, and processing methods, while background diet quality and habitual consumption patterns vary between populations. Importantly, the contribution of live microorganisms likely differs across fermented food categories, depending on microbial viability at the time of consumption. While products such as fermented milks are typically consumed with live cultures, other fermented foods included in this analysis (e.g., bread, chocolate) often contain few or no viable microorganisms due to processing steps such as heating, baking, or roasting. In these cases, observed associations are more plausibly mediated by fermentation-derived metabolites and microbially transformed compounds rather than by direct microbial exposure (22, 87, 96). These factors likely contribute to the variability in effect sizes and may influence both microbial viability and fermentation-derived metabolites.

Several fermented foods, such as fermented deli foods and vegetables, are not included due to lack of relevant data. In particular, fermented meat and fermented vegetable products could not be evaluated quantitatively, as only one prospective cohort study has distinguished these foods as separate dietary exposures in relation to mortality outcomes (50). This reflects a broader limitation of the epidemiological literature, in which fermented meats are typically grouped with other processed meat products, while fermented vegetables are inconsistently defined or inadequately captured in dietary assessment instruments. As highlighted in our previously published systematic review (22), both categories are highly heterogeneous with respect to fermentation practices, microbial composition, salt content, and post-fermentation processing (e.g., curing, smoking, or pasteurization), which may introduce both beneficial and adverse health effects. Consequently, the scarcity of well-characterized exposure data limits comparability across studies and hinders the ability to isolate fermentation-specific associations with mortality.

For cause-specific mortality, not all cohorts implemented competing-risk models, which may introduce additional variability in outcome estimates. Nevertheless, although the effect sizes of some associations are modest, they are consistent with what is typically observed in nutritional epidemiology. Such magnitudes are widely regarded as meaningful, particularly for long-term outcomes such as all-cause and cause-specific mortality, where small relative effects at the individual level can translate into substantial public health impact at the population level (97). Overall, the associations reported here should be interpreted cautiously, as causal inference cannot be established from observational data. It should be also noted that given the very limited number of studies retrieved, and the overall lack of robust, high-quality data on the association between fermented food intake and cancer-specific mortality, we were unable to draw meaningful conclusions or explore these findings in greater depth.

## 6 Conclusions

This meta-analysis is the first to evaluate the evidence on how foods produced through fermentation may confer health protection. Our results are consistent with those of the latest meta-analyses on the protective effects exhibited by fermented dairy products (fermented milks, cheese) and CVD mortality, while the present meta-analysis also showed a protective effect exhibited by fermented milk consumption on cancer mortality. Furthermore, chocolate appeared to be protective against CVD mortality, while miso consumption is of a neutral effect on gastrointestinal cancer mortality. However, it should be noted that the current evidence base is limited by the small number of high-quality, prospective cohort studies and randomized controlled trials and, as a result, the overall certainty of the evidence remains moderate. To strengthen the evidence base, upcoming research should include well-designed cohort studies that examine multiple fermented foods in diverse, representative populations and provide detailed characterization of the specific items studied, including substrate origin, microbial composition, and processing conditions. In addition, clinical studies using standardized fermented foods with clearly defined microbial loads and fermentation parameters (along with randomized trials assessing dose–response effects, microbial viability, and mechanistic biomarkers) will be essential to clarify causality and biological pathways. Cross-cultural comparisons comparing different fermentation traditions and matrices would further help determine the generalizability of observed associations.

## Data Availability

The original contributions presented in the study are included in the article/Supplementary material, further inquiries can be directed to the corresponding author.
