# Supplementary material for: Fermented foods consumption, all-cause, and cause-specific mortality: a meta-analysis of prospective cohort studies
Source: Front Nutr. 2026 Feb 26;13:1714437. doi: 10.3389/fnut.2026.1714437 (PMC12979560; doi:10.3389/fnut.2026.1714437)
Supplement: Supplementary file 1 [file Data_Sheet_1.docx]

Supplementary Material

# Methods

**Table S1.** Search string used in PubMed.

| **No.** | **Query PubMed** |
| --- | --- |
| #1 | "Fermented Foods"[Mesh] OR "Fermentation"[Mesh] OR ("Food"[Mesh] AND ferment*[tiab]) OR ((ferment*[tiab] OR cultur*[tiab] OR leaven*[tiab]) AND (food*[tiab] OR drink*[tiab] OR beverage*[tiab])) OR "Fermented product"[tiab:~6] OR "Fermented products"[tiab:~6] OR "cultured product"[tiab:~6] OR "cultured products"[tiab:~6] OR "product fermentation"[tiab:~6] OR "products fermentation"[tiab:~6] OR "starter culture*"[tiab] OR ((ferment*[tiab] OR culture*[tiab] OR sour*[tiab]) AND (milk[tiab] OR dairy[tiab])) OR buttermilk[tiab] OR sour cream*[tiab] OR cheese*[tiab] OR yoghurt[tiab] OR yogurt[tiab] OR "yoghourt"[tiab] OR "yakult"[tiab] OR "quark"[tiab] OR "kefir"[tiab] OR "lassi"[tiab] OR "kumis"[tiab] OR "koumiss"[tiab] OR "kajmak"[tiab] OR "airag"[tiab] OR "ayran"[tiab] OR "calpis"[tiab] OR "borhani"[tiab] OR "chal"[tiab] OR "doogh"[tiab] OR kvass[tiab] OR skyr[tiab] OR amasi[tiab] OR bouza[tiab] OR butter*[tiab] OR chal[tiab] OR filmjolk[tiab] OR kishk[tiab] OR labne*[tiab] OR ((Ferment*[tiab] OR cultur*[tiab] OR cured[tiab]) AND (meat*[tiab] OR fish*[tiab] OR seafood*[tiab] OR shellfish[tiab] OR sausage*[tiab])) OR "salami"[tiab] OR "pepperoni"[tiab] OR peperoni[tiab] OR "chorizo"[tiab] OR "cervelat"[tiab] OR "mettwurst"[tiab] OR "summer sausage"[tiab] OR "sucuk"[tiab] OR "dried meat"[tiab] OR "dried sausage"[tiab] OR "dry sausage"[tiab] OR "fish sauce"[tiab] OR "shrimp paste"[tiab] OR "shrimp sauce"[tiab] OR "oyster sauce"[tiab] OR "prosciutto"[tiab] OR "pancetta"[tiab] OR "saucisson"[tiab] OR sucuk[tiab] OR ((Ferment*[tiab] OR cultur*[tiab]) AND (fruit*[tiab] OR vegetable*[tiab] OR coconut*[tiab] OR almond*[tiab] OR hazelnut*[tiab] OR nut[tiab] OR cucumber*[tiab] OR lemon*[tiab] OR citrus[tiab] OR cabbage*[tiab] OR cauliflower*[tiab] OR pepper*[tiab] OR carrot*[tiab] OR olive*[tiab] OR onion*[tiab] OR sago[tiab])) OR "sauerkraut"[tiab] OR "table olive"[tiab] OR pickle*[tiab] OR "kimchi"[tiab] OR "paocai"[tiab] OR torshi[tiab] OR ((Ferment*[tiab] OR cultur*[tiab]) AND (tea[tiab] OR teas[tiab] OR juice*[tiab])) OR "beer"[tiab] OR "wine"[tiab] OR "cider*"[tiab] OR shochu[tiab] OR "kombucha*"[tiab] OR "pulque"[tiab] OR puer[tiab] OR pu’er[tiab] OR pu-er[tiab] OR pu-erh[tiab] OR "pu erh"[tiab] OR "fuzhuan"[tiab] OR "dark tea*"[tiab] OR "yellow tea*"[tiab] OR coffee[tiab] OR shalgam[tiab] OR hardaliye[tiab] OR ((Ferment*[tiab] OR cultur*[tiab]) AND (soy[tiab] OR soya[tiab] OR bean*[tiab] OR pea[tiab] OR peas[tiab] OR lentil*[tiab] OR chickpea*[tiab] OR legume*[tiab] OR pulse*[tiab] OR (poi[tiab])) OR "soy sauce"[tiab] OR "soya sauce"[tiab] OR "soybean paste"[tiab] OR "miso"[tiab] OR "tempeh"[tiab] OR tempe[tiab] OR "natto"[tiab] OR "doenjang"[tiab] OR "doubanjiang"[tiab] OR douchi[tiab] OR "gochujang"[tiab] OR cheonggukjang[tiab] OR tsukemono[tiab] OR garri[tiab] OR ((Ferment*[tiab] OR cultur*[tiab] OR leaven*[tiab]) AND (cereal*[tiab] OR grain*[tiab] OR wheat*[tiab] OR oat[tiab] OR oats[tiab] OR rice*[tiab] OR millet*[tiab] OR sorghum*[tiab] OR maize*[tiab] OR rye[tiab] OR barley*[tiab] OR chia[tiab] OR oilseed*[tiab] OR teff[tiab])) OR "bread"[tiab] OR "sourdough"[tiab] OR "crispbread"[tiab] OR "boza"[tiab] OR "ogi"[tiab] OR dosa[tiab] OR "tarhana"[tiab] OR "buckwheat"[tiab] OR "spelt"[tiab] OR "einkorn"[tiab] OR "quinoa"[tiab] OR "amaranth"[tiab] OR "tef"[tiab] OR "bushera"[tiab] OR chica[tiab] OR chicha[tiab] OR choujiu[tiab] OR injera[tiab] OR mahewu[tiab] OR ogiri[tiab] OR pozol[tiab] OR ugba[tiab]) OR ((Ferment*[tiab] OR cultur*[tiab]) AND ("condiment*"[tiab] OR relish*[tiab] OR horseradish[tiab] OR "dressing*"[tiab] OR "seasoning*"[tiab] OR "sauce*"[tiab] OR cocoa*[tiab] OR tuber[tiab] OR "acetic acid"[tiab])) OR "chocolate*"[tiab] OR "vinegar*"[tiab] OR "tabasco"[tiab] OR "sriracha"[tiab] OR "Worcestershire"[tiab] OR "Worcester"[tiab] |
| #2 | "Diet"[Mesh] OR "Life Style"[Mesh] OR "Eating"[Mesh] OR "Feeding Behavior"[Mesh] OR ((food[tiab] OR macronutrient*[tiab] OR eating[tiab]) AND (intake*[tiab] OR habit*[tiab] OR behavior*[tiab] OR pattern*[tiab])) OR diet*[tiab] OR intake[tiab] OR ingestion[tiab] OR suppl*[tiab] OR consumption[tiab] OR meal*[tiab] OR nutrient*[tiab] OR nutrit*[tiab] |
| #3 | "Mortality"[Mesh] OR "Death"[Mesh] OR "mortality"[Subheading] OR ((Mortalit*[tiab] OR death*[tiab] OR fatal*[tiab] OR survival[tiab]) AND ("Risk Factors"[Mesh] OR factor*[tiab] OR risk*[tiab] OR rate*[tiab] OR hazard ratio*[tiab])) |
| #4 | #1 AND #2 AND #3 |
| #5 | "Diet Surveys"[Mesh] OR "Cohort Studies"[Mesh] OR cohort*[Tiab] OR prospective[Tiab] OR longitudinal[Tiab] |
| #6 | "Epidemiologic Methods"[Mesh:NoExp] OR "Epidemiologic Studies"[Mesh] OR "Observational Studies as Topic"[Mesh] OR "Clinical Studies as Topic"[Mesh] OR "Single-Case Studies as Topic"[Mesh] OR "Organizational Case Studies"[Mesh] OR observational study[Publication Type] OR validation study[Publication Type] OR clinical study[Publication Type] OR case reports[Publication Type] OR "observational study"[tiab:~3] OR "observational studies"[tiab:~3] OR "observational design"[tiab:~3] OR "observational analysis"[tiab:~3] OR "observational analyses"[tiab:~3] OR ((cohort*[tiab] OR prospective[tiab] OR follow-up[tiab] OR longitudinal[tiab] OR long-term[tiab] OR retrospective[tiab]) AND (study[tiab] OR studies[tiab] OR design[tiab] OR analysis[tiab] OR analyses[tiab] OR data[tiab] OR review[tiab])) OR case control*[tiab] OR case comparison*[tiab] OR case-referent[tiab] OR "population study"[tiab:~3] OR "population studies"[tiab:~3] OR "population analysis"[tiab:~3] OR "population analyses"[tiab:~3] OR "descriptive study"[tiab:~3] OR "descriptive studies"[tiab:~3] OR "descriptive design"[tiab:~3] OR "descriptive analysis"[tiab:~3] OR "descriptive analyses"[tiab:~3] OR "multidimensional study"[tiab:~3] OR "multidimensional studies"[tiab:~3] OR "multidimensional design"[tiab:~3] OR "multidimensional analysis"[tiab:~3] OR "multidimensional analyses"[tiab:~3] OR "cross-sectional study"[tiab:~3] OR "cross-sectional studies"[tiab:~3] OR "cross-sectional design"[tiab:~3] OR "cross-sectional analysis"[tiab:~3] OR "cross-sectional analyses"[tiab:~3] OR "cross-sectional research"[tiab:~3] OR "cross-sectional survey"[tiab:~3] OR "cross-sectional findings"[tiab:~3] OR natural experiment*[tiab] OR quasi experiment*[tiab] OR "nonexperimental study"[tiab:~3] OR "nonexperimental studies"[tiab:~3] OR "nonexperimental design"[tiab:~3] OR "nonexperimental analysis"[tiab:~3] OR "nonexperimental analyses"[tiab:~3] OR "prevalence study"[tiab:~3] OR "prevalence studies"[tiab:~3] OR "prevalence analysis"[tiab:~3] OR "prevalence analyses"[tiab:~3] OR case series[tiab] OR "case report"[tiab:~3] OR "case reports"[tiab:~3] OR "case study"[tiab:~3] OR "case studies"[tiab:~3] OR "case histories"[tiab:~3] |
| #7 | "systematic review" |
| #8 | #5 OR #6 OR #7 |
| #9 | #4 AND #8 |
| #10 | #9 NOT (("Child"[Mesh] OR "Infant"[Mesh] OR "Adolescent"[Mesh]) NOT "Adult"[Mesh]) |
| #11 | #10 NOT (("Animals"[Mesh] OR "Animal Experimentation"[Mesh] OR "Models, Animal"[Mesh] OR "Vertebrates"[Mesh]) NOT ("Humans"[Mesh] OR "Human Experimentation"[Mesh])) |
| #12 | #11 NOT ("Breast Feeding"[Majr] OR "Milk, Human"[Majr]) |
| #13 | #12 AND (English[Filter]) |
| #14 | #13 AND (("1970/01/01"[Date - Publication] : "2023/08/31"[Date - Publication])) |

# Results

**2.1 Metanalyses on yogurt and its association with mortality risk**

***2.1.1 Yogurt (only) consumption, all-cause and cause-specific mortality***

The meta-analysis evaluating yogurt consumption included studies that specifically examined yogurt intake, comprising 828,256 participants and 79,456 deaths (1–13). ***Figure S1*** presents the forest plots from the random-effects meta-analysis comparing the highest versus lowest categories of yogurt consumption for all-cause and cause-specific mortality. The combined estimate indicated that higher yogurt consumption was associated with a significantly lower risk of all-cause mortality (pooled RR = 0.933, p<0.001), with low heterogeneity (I² = 25.50%). Sensitivity analyses using a leave-one-study-out approach confirmed that no individual study materially influenced the pooled association.

In regard to CVD mortality (1,3,5–12,14), the random-effects model showed a non-significant inverse association (pooled RR = 0.951, p=0.06) and low heterogeneity (I² = 17.92%). The leave-one-out analyses indicated stable findings across studies, and none of the evaluated moderators meaningfully influenced the pooled effect.

For overall cancer mortality (3,5–12,15,16), yogurt consumption was not significantly associated with risk (pooled RR = 0.971, p = 0.22; I² = 17.86%). Similarly, no significant association was detected between yogurt consumption and gastrointestinal cancer (pooled RR = 0.923, p=0.45; low heterogeneity) (15–18) and lung cancer mortality (pooled RR = 0.878, p=0.41; low heterogeneity) (15,16,19). Reproductive cancer mortality (20,21) was not meta-analyzed due to the limited available information.

**
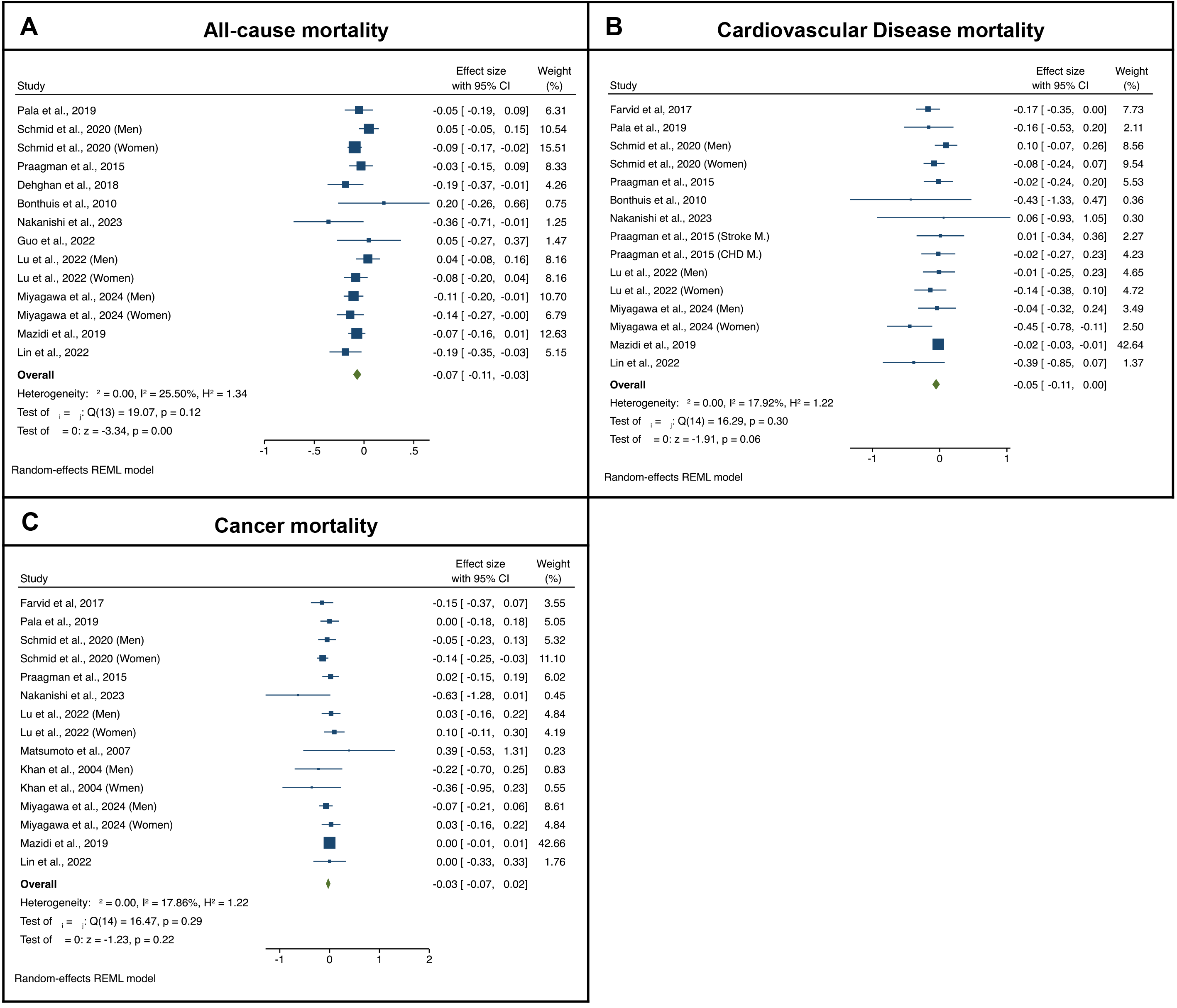
**

**Figure S1.** Forest-plots illustrating the association between yogurt consumption (highest quartile to the lowest quartile) and (A) all-cause, (B) cardiovascular disease (CVD), and (C) cancer mortality. Results are presented as pooled log-transformed Risk Ratios and their corresponding 95% confidence intervals.

# References

1. Bonthuis M, Hughes MCB, Ibiebele TI, Green AC, Van Der Pols JC. Dairy consumption and patterns of mortality of Australian adults. Eur J Clin Nutr. 2010 June;64(6):569–77.

2. Dehghan M, Mente A, Rangarajan S, Sheridan P, Mohan V, Iqbal R, et al. Association of dairy intake with cardiovascular disease and mortality in 21 countries from five continents (PURE): a prospective cohort study. The Lancet. 2018 Nov;392(10161):2288–97.

3. Farvid MS, Malekshah AF, Pourshams A, Poustchi H, Sepanlou SG, Sharafkhah M, et al. Dairy food intake and all-cause, cardiovascular disease, and cancer mortality. Am J Epidemiol. 2017 Apr 15;185(8):697–711.

4. Guo J, Givens DI, Heitmann BL. Association between dairy consumption and cardiovascular disease events, bone fracture and all-cause mortality. Huang HK, editor. PLoS ONE. 2022 Sept 9;17(9):e0271168.

5. Lin P, Gui X, Liang Z, Wang T. Association of yogurt and dietary supplements containing probiotic consumption with all-cause and cause-specific mortality in US adults: a population-based cohort study. Front Nutr. 2022 Feb 7;9:803076.

6. Lu Y, Sugawara Y, Matsuyama S, Fukao A, Tsuji I. Association of dairy intake with all-cause, cancer, and cardiovascular disease mortality in Japanese adults: a 25-year population-based cohort. Eur J Nutr. 2022 Apr;61(3):1285–97.

7. Mazidi M, Mikhailidis DP, Sattar N, Howard G, Graham I, Banach M. Consumption of dairy product and its association with total and cause specific mortality – a population-based cohort study and meta-analysis. Clin Nutr. 2019 Dec;38(6):2833–45.

8. Miyagawa N, Takashima N, Harada A, Kadota A, Kondo K, Miura K, et al. Dairy intake and all-cause, cancer, and cardiovascular disease mortality risk in a large Japanese population: a 12-year follow-up of the J-MICC study. JAT. 2024;65049.

9. Nakanishi A, Homma E, Osaki T, Sho R, Souri M, Sato H, et al. Association between milk and yogurt intake and mortality: a community-based cohort study (Yamagata study). BMC Nutr. 2021 Dec;7(1):33.

10. Pala V, Sieri S, Chiodini P, Masala G, Palli D, Mattiello A, et al. Associations of dairy product consumption with mortality in the European Prospective Investigation into Cancer and Nutrition (EPIC)–Italy cohort. Am J Clin Nutr. 2019 Nov;110(5):1220–30.

11. Praagman J, Dalmeijer GW, Van Der Schouw YT, Soedamah-Muthu SS, Monique Verschuren WM, Bas Bueno-de-Mesquita H, et al. The relationship between fermented food intake and mortality risk in the European Prospective Investigation into Cancer and Nutrition-Netherlands cohort. Br J Nutr. 2015 Feb 14;113(3):498–506.

12. Schmid D, Song M, Zhang X, Willett WC, Vaidya R, Giovannucci EL, et al. Yogurt consumption in relation to mortality from cardiovascular disease, cancer, and all causes: a prospective investigation in 2 cohorts of US women and men. Am J Clin Nutr. 2020 Mar;111(3):689–97.

13. Sluik D, Boeing H, Li K, Kaaks R, Johnsen NF, Tjønneland A, et al. Lifestyle factors and mortality risk in individuals with diabetes mellitus: are the associations different from those in individuals without diabetes? Diabetologia. 2014 Jan;57(1):63–72.

14. Praagman J, Franco OH, Ikram MA, Soedamah-Muthu SS, Engberink MF, Van Rooij FJA, et al. Dairy products and the risk of stroke and coronary heart disease: the Rotterdam study. Eur J Nutr. 2015 Sept;54(6):981–90.

15. Matsumoto M, Ishikawa S, Nakamura Y, Kayaba K, Kajii E. Consumption of dairy products and cancer risks. J Epidemiol. 2007;17(2):38–44.

16. Khan M, Goto R, Kobayashi K, Suzumura S, Nagata Y, Sonoda T, et al. Dietary habits and cancer mortality among middle aged and older Japanese living in Hokkaido, Japan by cancer site and sex. APJCP. 2004;5:58–65.

17. Kojima M, Wakai K, Tamakoshi K, Tokudome S, Toyoshima H, Watanabe Y, et al. Diet and colorectal cancer mortality: results from the Japan collaborative cohort study. Nutr Cancer. 2004 Sept;50(1):23–32.

18. Tokui N, Yoshimura T, Fujino Y, Mizoue T, Hoshiyama Y, Yatsuya H, et al. Dietary habits and stomach cancer risk in the JACC study. J Epidemiol. 2005;15(Supplement_II):S98–108.

19. Ozasa K, Watanabe Y, Ito Y, Suzuki K, Tamakoshi A, Seki N, et al. Dietary habits and risk of lung cancer death in a large‐scale cohort study (JACC study) in Japan by sex and smoking habit. Japanese Journal of Cancer Research. 2001 Dec;92(12):1259–69.

20. Park Y, Mitrou PN, Kipnis V, Hollenbeck A, Schatzkin A, Leitzmann MF. Calcium, dairy foods, and risk of incident and fatal prostate cancer: the NIH-AARP diet and health study. Am J Epidemiol. 2007 Aug 28;166(11):1270–9.

21. Sakauchi F, Khan MMH, Mori M, Kubo T, Fujino Y, Suzuki S, et al. Dietary habits and risk of ovarian cancer death in a large-scale cohort study (JACCstudy) in Japan. Nutr Cancer. 2007 June 8;57(2):138–45.
