# Supplementary material for: Fermented foods consumption, all-cause, and cause-specific mortality: a meta-analysis of prospective cohort studies
Source: Front Nutr. 2026 Feb 26;13:1714437. doi: 10.3389/fnut.2026.1714437 (PMC12979560; doi:10.3389/fnut.2026.1714437)
Supplement: Supplementary file 3 [file Data_Sheet_3.pdf]

Supplementary Material 3 Details of excluded papers with reasons for exclusion.

Title	Author	Publication year	DOI	Reason for exclusion				
				**Popula-tion**	**Exposure**	**Outcome**	**Study design**	**Other reasons**
Dietary carbohydrate intake and mortality: a prospective cohort study and meta-analysis.	Seidelmann, Sara B.; Claggett, Brian; Cheng, Susan; Henglin, Mir; Shah, Amil; Steffen, Lyn M.; Folsom, Aaron R.; Rimm, Eric B.; Willett, Walter C.; Solomon, Scott D.	2018	10.1016/S2468-2667(18)30135-X		✓			
Tea Consumption and All-Cause and Cause-Specific Mortality in the UK Biobank : A Prospective Cohort Study.	Inoue-Choi, Maki; Ramirez, Yesenia; Cornelis, Marilyn C.; Berrington de González, Amy; Freedman, Neal D.; Loftfield, Erikka	2022	10.7326/M22-0041		✓			
Red meat intake and risk of coronary heart disease among US men: prospective cohort study.	Al-Shaar, Laila; Satija, Ambika; Wang, Dong D.; Rimm, Eric B.; Smith-Warner, Stephanie A.; Stampfer, Meir J.; Hu, Frank B.; Willett, Walter C.	2020	10.1136/bmj.m4141			✓		
Egg and cholesterol consumption and mortality from cardiovascular and different causes in the United States: A population-based cohort study.	Zhuang, Pan; Wu, Fei; Mao, Lei; Zhu, Fanghuan; Zhang, Yiju; Chen, Xiaoqian; Jiao, Jingjing; Zhang, Yu	2021	10.1371/journal.pmed.1003508		✓			
Dietary protein intake and all-cause and cause-specific mortality: results from the Rotterdam Study and a meta-analysis of prospective cohort studies.	Chen, Zhangling; Glisic, Marija; Song, Mingyang; Aliahmad, Hamid A.; Zhang, Xiaofang; Moumdjian, Alice C.; Gonzalez-Jaramillo, Valentina; van der Schaft, Niels; Bramer, Wichor M.; Ikram, Mohammad Arfan; Voortman, Trudy	2020	10.1007/s10654-020-00607-6		✓			
Association of dairy intake with cardiovascular disease and mortality in 21 countries from five continents (PURE): a prospective cohort study.	Dehghan, Mahshid; Mente, Andrew; Rangarajan, Sumathy; Sheridan, Patrick; Mohan, Viswanathan; Iqbal, Romaina; Gupta, Rajeev; Lear, Scott; Wentzel-Viljoen, Edelweiss; Avezum, Alvaro; Lopez-Jaramillo, Patricio; Mony, Prem; Varma, Ravi Prasad; Kumar, Rajesh; Chifamba, Jephat; Alhabib, Khalid F.; Mohammadifard, Noushin; Oguz, Aytekin; Lanas, Fernando; Rozanska, Dorota; Bostrom, Kristina Bengtsson; Yusoff, Khalid; Tsolkile, Lungiswa P.; Dans, Antonio; Yusufali, AfzalHussein; Orlandini, Andres; Poirier, Paul; Khatib, Rasha; Hu, Bo; Wei, Li; Yin, Lu; Deeraili, Ai; Yeates, Karen; Yusuf, Rita; Ismail, Noorhassim; Mozaffarian, Dariush; Teo, Koon; Anand, Sonia S.; Yusuf, Salim	2018	10.1016/S0140-6736(18)31812-9		✓			

Causal relationship from coffee consumption to diseases and mortality: a review of observational and Mendelian randomization studies including cardiometabolic diseases, cancer, gallstones and other diseases.	Nordestgaard, Ask T.	2022	10.1007/s00394-021-02650-9				✓	
Intake of Flavonoids and Flavonoid-Rich Foods and Mortality Risk Among Individuals With Parkinson Disease: A Prospective Cohort Study.	Zhang, Xinyuan; Molsberry, Samantha A.; Yeh, Tian-Shin; Cassidy, Aedin; Schwarzschild, Michael A.; Ascherio, Alberto; Gao, Xiang	2022	10.1212/WNL.000000000000013275	✓	✓			
Dietary phytoestrogens and total and cause-specific mortality: results from 2 prospective cohort studies.	Chen, Zhangling; Qian, Frank; Hu, Yang; Voortman, Trudy; Li, Yanping; Rimm, Eric B.; Sun, Qi	2023	10.1016/j.ajcnut.2022.10.019		✓			
Health and sustainability outcomes of vegetarian dietary patterns: a revisit of the EPIC-Oxford and the Adventist Health Study-2 cohorts.	Segovia-Siapco, Gina; Sabaté, Joan	2019	10.1038/s41430-018-0310-z		✓			
Diet and overall survival in elderly people.	Trichopoulou, A.; Kouris-Blazos, A.; Wahlqvist, M. L.; Gnardellis, C.; Lagiou, P.; Polychronopoulos, E.; Vassilakou, T.; Lipworth, L.; Trichopoulos, D.	1995	10.1136/bmj.311.7018.1457		✓			
Associations between dietary patterns and the incidence of total and fatal cardiovascular disease and all-cause mortality in 116,806 individuals from the UK Biobank: a prospective cohort study.	Gao, Min; Jebb, Susan A.; Aveyard, Paul; Ambrosini, Gina L.; Perez-Cornago, Aurora; Carter, Jennifer; Sun, Xinying; Piernas, Carmen	2021	10.1186/s12916-021-01958-x		✓			
Plant-Based Diets and All-cause and Cardiovascular Mortality in a Nationwide Cohort in Spain: The ENRICA Study.	Delgado-Velandia, M.; Maroto-Rodríguez, J.; Ortolá, R.; García-Esquinas, E.; Rodríguez-Artalejo, F.; Sotos-Prieto, M.	2022	10.1016/j.mayocp.2022.06.008		✓			
Dairy products, calcium, and prostate cancer risk: a systematic review and meta-analysis of cohort studies.	Aune, Dagfinn; Navarro Rosenblatt, Deborah A.; Chan, Doris S. M.; Vieira, Ana Rita; Vieira, Rui; Greenwood, Darren C.; Vatten, Lars J.; Norat, Teresa	2015	10.3945/ajcn.113.067157			✓		

The Isocaloric Substitution of Plant-Based and Animal-Based Protein in Relation to Aging-Related Health Outcomes: A Systematic Review.	Zheng, Jiali; Zhu, Tianren; Yang, Guanghuan; Zhao, Longgang; Li, Fangyu; Park, Yong-Moon; Tabung, Fred K.; Steck, Susan E.; Li, Xiaoguang; Wang, Hui	2022	10.3390/nu14020272		✓			
Coffee reduces the risk of death after acute myocardial infarction: a meta-analysis.	Brown, Oliver I.; Allgar, Victoria; Wong, Kenneth Y.-K.	2016	10.1097/MCA.000000000000000397	✓				
Japanese diet and survival time: The Ohsaki Cohort 1994 study.	Abe, Saho; Zhang, Shu; Tomata, Yasutake; Tsuduki, Tsuyoshi; Sugawara, Yumi; Tsuji, Ichiro	2020	10.1016/j.clnu.2019.02.010		✓			
Dietary Soy Consumption and Cardiovascular Mortality among Chinese People with Type 2 Diabetes.	Wang, Xiaowen; Lv, Jun; Yu, Canqing; Li, Liming; Hu, Yonghua; Qin, Li-Qiang; Dong, Jia-Yi	2021	10.3390/nu13082513	✓	✓			
Highly salted food and mountain herbs elevate the risk for stomach cancer death in a rural area of Japan.	Kurosawa, Michiko; Kikuchi, Shogo; Xu, Jun; Inaba, Yutaka	2006	10.1111/j.1440-1746.2006.04290.x		✓			
Prospective Associations between Single Foods, Alzheimer's Dementia and Memory Decline in the Elderly.	Fischer, Karina; Melo van Lent, Debora; Wolfsgruber, Steffen; Weinhold, Leonie; Kleineidam, Luca; Bickel, Horst; Scherer, Martin; Eisele, Marion; van den Bussche, Hendrik; Wiese, Birgitt; König, Hans-Helmut; Weyerer, Siegfried; Pentzek, Michael; Röhr, Susanne; Maier, Wolfgang; Jessen, Frank; Schmid, Matthias; Riedel-Heller, Steffi G.; Wagner, Michael	2018	10.3390/nu10070852	✓		✓		
Aging, food, culture and health.	Wahlqvist, M. L.; Kouris-Blazos, A.; Hsa-Hage, B. H.	1997			✓			
The Southern European Atlantic Diet and all-cause mortality in older adults.	Carballo-Casla, Adrián; Ortolá, Rosario; García-Esquinas, Esther; Oliveira, Andreia; Sotos-Prieto, Mercedes; Lopes, Carla; Lopez-Garcia, Esther; Rodríguez-Artalejo, Fernando	2021	10.1186/s12916-021-01911-y		✓			
Safety of coffee consumption after myocardial infarction: A systematic review and meta-analysis.	Ribeiro, Eduardo M.; Alves, Mariana; Costa, João; Ferreira, Joaquim J.; Pinto, Fausto J.; Caldeira, Daniel	2020	10.1016/j.numecd.2020.07.016	✓				
Polyphenol intake and mortality: A nationwide cohort study in the adult population of Spain.	María Mérida, Diana; Vitelli-Storelli, Facundo; Moreno-Franco, Belén; Rodríguez-Ayala, Montserrat; López-García, Esther; Banegas, José R.; Rodríguez-Artalejo, Fernando; Guallar-Castillón, Pilar	2023	10.1016/j.clnu.2023.05.020		✓			

Association of flavonoid-rich foods and flavonoids with risk of all-cause mortality.	Ivey, Kerry L.; Jensen, Majken K.; Hodgson, Jonathan M.; Eliassen, A. Heather; Cassidy, Aedín; Rimm, Eric B.	2017	10.1017/S0007114517001325		✓			
Dairy consumption and mortality after myocardial infarction: a prospective analysis in the Alpha Omega Cohort.	Cruijsen, Esther; Jacobo Cejudo, Maria G.; Küpers, Leanne K.; Busstra, Maria C.; Geleijnse, Johanna M.	2021	10.1093/ajcn/nqab026	✓				
Dietary patterns related to total mortality and cancer mortality in the United States.	Entwistle, Marcela R.; Schweizer, Donald; Cisneros, Ricardo	2021	10.1007/s10552-021-01478-2		✓			
Dairy fat and risk of cardiovascular disease in 3 cohorts of US adults.	Chen, Mu; Li, Yanping; Sun, Qi; Pan, An; Manson, JoAnn E.; Rexrode, Kathryn M.; Willett, Walter C.; Rimm, Eric B.; Hu, Frank B.	2016	10.3945/ajcn.116.134460		✓	✓		
Nutrient-rich foods, cardiovascular diseases and all-cause mortality: the Rotterdam study.	Streppel, M. T.; Sluik, D.; van Yperen, J. F.; Geelen, A.; Hofman, A.; Franco, O. H.; Witteman, J. C. M.; Feskens, E. J. M.	2014	10.1038/ejcn.2014.35		✓			
Mediterranean diet and mortality in Switzerland: an alpine paradox?	Vormund, Kerstin; Braun, Julia; Rohrmann, Sabine; Bopp, Matthias; Ballmer, Peter; Faeh, David	2015	10.1007/s00394-014-0695-y		✓			
Chocolate intake and heart disease and stroke in the Women's Health Initiative: a prospective analysis.	Greenberg, James A.; Manson, JoAnn E.; Neuhouser, Marian L.; Tinker, Lesley; Eaton, Charles; Johnson, Karen C.; Shikany, James M.	2018	10.1093/ajcn/nqy073			✓		
Rye bread consumption in early life and reduced risk of advanced prostate cancer.	Torfadottir, Johanna E.; Valdimarsdottir, Unnur A.; Mucci, Lorelei; Stampfer, Meir; Kasperzyk, Julie L.; Fall, Katja; Tryggvadottir, Laufey; Aspelund, Thor; Olafsson, Orn; Harris, Tamara B.; Jonsson, Eirikur; Tulinius, Hrafn; Adami, Hans-Olov; Gudnason, Vilmundur; Steingrimsdottir, Laufey	2012	10.1007/s10552-012-9965-2			✓		
Red meat consumption and mortality: results from 2 prospective cohort studies.	Pan, An; Sun, Qi; Bernstein, Adam M.; Schulze, Matthias B.; Manson, JoAnn E.; Stampfer, Meir J.; Willett, Walter C.; Hu, Frank B.	2012	10.1001/archinternmed.2011.2287		✓			
Change in habitual intakes of flavonoid-rich foods and mortality in US males and females.	Bondonno, Nicola P.; Liu, Yan Lydia; Zheng, Yan; Ivey, Kerry; Willett, Walter C.; Stampfer, Meir J.; Rimm, Eric B.; Cassidy, Aedín	2023	10.1186/s12916-023-02873-z		✓			
Study on soy isoflavone consumption and risk of breast cancer and survival.	Kang, Hong-Bin; Zhang, Ya-Feng; Yang, Jin-Dun; Lu, Kuan-Liang	2012	10.7314/apjcp.2012.13.3.995	✓	✓			

Total polyphenol intake, polyphenol subtypes and incidence of cardiovascular disease: The SUN cohort study.	Mendonça, R. D.; Carvalho, N. C.; Martin-Moreno, J. M.; Pimenta, A. M.; Lopes, A. C. S.; Gea, A.; Martinez-Gonzalez, M. A.; Bes-Rastrollo, M.	2019	10.1016/j.numecd.2018.09.012		✓	✓		
Dietary patterns and mortality in a Chinese population.	Odegaard, Andrew O.; Koh, Woon-Puay; Yuan, Jian-Min; Gross, Myron D.; Pereira, Mark A.	2014	10.3945/ajcn.114.086124		✓			
Prospective investigation of major dietary patterns and risk of cardiovascular mortality in Bangladesh.	Chen, Yu; McClintock, Tyler R.; Segers, Stephanie; Parvez, Faruque; Islam, Tariqul; Ahmed, Alauddin; Rakibuz-Zaman, Muhammad; Hasan, Rabiul; Sarwar, Golam; Ahsan, Habibul	2013	10.1016/j.ijcard.2012.04.041		✓			
Dietary patterns and the risk of CVD and all-cause mortality in older British men.	Atkins, Janice L.; Whincup, Peter H.; Morris, Richard W.; Lennon, Lucy T.; Papacosta, Olia; Wannamethee, S. Goya	2016	10.1017/S0007114516003147		✓			
Egg and egg-sourced cholesterol consumption in relation to mortality: Findings from population-based nationwide cohort.	Zhuang, Pan; Jiao, Jingjing; Wu, Fei; Mao, Lei; Zhang, Yu	2020	10.1016/j.clnu.2020.03.019		✓			
Soy and isoflavone consumption and subsequent risk of prostate cancer mortality: the Japan Public Health Center-based Prospective Study.	Sawada, Norie; Iwasaki, Motoki; Yamaji, Taiki; Shimazu, Taichi; Inoue, Manami; Tsugane, Shoichiro	2020	10.1093/ije/dyaa177			✓		
Dietary soy intake is not associated with risk of cardiovascular disease mortality in Singapore Chinese adults.	Talaei, Mohammad; Koh, Woon-Puay; van Dam, Rob M.; Yuan, Jian-Min; Pan, An	2014	10.3945/jn.114.190454		✓			
Calcium and vitamin D intake and mortality: results from the Canadian Multicentre Osteoporosis Study (CaMos).	Langsetmo, Lisa; Berger, Claudie; Kreiger, Nancy; Kovacs, Christopher S.; Hanley, David A.; Jamal, Sophie A.; Whiting, Susan J.; Genest, Jacques; Morin, Suzanne N.; Hodsman, Anthony; Prior, Jerilynn C.; Lentle, Brian; Patel, Millan S.; Brown, Jacques P.; Anastasiades, Tassos; Towheed, Tanveer; Josse, Robert G.; Papaioannou, Alexandra; Adachi, Jonathan D.; Leslie, William D.; Davison, K. Shawn; Goltzman, David	2013	10.1210/jc.2013-1516		✓			
Association of Major Dietary Protein Sources With All-Cause and Cause-Specific Mortality: Prospective Cohort Study.	Sun, Yangbo; Liu, Buyun; Snetselaar, Linda G.; Wallace, Robert B.; Shadyab, Aladdin H.; Kroenke, Candyce H.; Haring, Bernhard; Howard, Barbara V.; Shikany, James M.; Valdiviezo, Carolina; Bao, Wei	2021	10.1161/JAHA.119.015553		✓			

Traditional Dietary Patterns and Risk of Mortality in a Longitudinal Cohort of the Salus in Apulia Study.	Zupo, Roberta; Sardone, Rodolfo; Donghia, Rossella; Castellana, Fabio; Lampignano, Luisa; Bortone, Ilaria; Misciagna, Giovanni; De Pergola, Giovanni; Panza, Francesco; Lozupone, Madia; Passantino, Andrea; Veronese, Nicola; Guerra, Vito; Boeing, Heiner; Giannelli, Gianluigi	2020	10.3390/nu12041070		✓			
Caffeinated coffee consumption and mortality after acute myocardial infarction.	Mukamal, Kenneth J.; Maclure, Malcolm; Muller, James E.; Sherwood, Jane B.; Mittleman, Murray A.	2004	10.1016/j.ahj.2003.12.038	✓				
A prospective study of coffee drinking and suicide in women.	Kawachi, I.; Willett, W. C.; Colditz, G. A.; Stampfer, M. J.; Speizer, F. E.	1996	10.1001/archinte.156.5.521			✓		
Arginine intake and risk of coronary heart disease mortality in elderly men.	Oomen, C. M.; van Erk, M. J.; Feskens, E. J.; Kok, F. J.; Kromhout, D.	2000	10.1161/01.atv.20.9.2134		✓			
Flavonoid intake and cardiovascular disease mortality: a prospective study in postmenopausal women.	Mink, Pamela J.; Scrafford, Carolyn G.; Barraj, Leila M.; Harnack, Lisa; Hong, Ching-Ping; Nettleton, Jennifer A.; Jacobs, David R. Jr	2007	10.1093/ajcn/85.3.895		✓			
Life-Style Habits in a High-Risk Area for Upper Gastrointestinal Cancers: a Population-Based Study from Shanxi, China.	Cheng, Yi-Kun; Yao, Shang-Man; Xu, Yi-Ran; Niu, Run-Gui	2016			✓	✓	✓	
Diet and survival of elderly Greeks: a link to the past.	Trichopoulou, A.; Kouris-Blazos, A.; Vassilakou, T.; Gnardellis, C.; Polychronopoulos, E.; Venizelos, M.; Lagiou, P.; Wahlqvist, M. L.; Trichopoulos, D.	1995	10.1093/ajcn/61.6.1346S		✓	✓		
Association Between Caffeine Intake and All-Cause and Cause-Specific Mortality: A Population-Based Prospective Cohort Study.	Tsujimoto, Tetsuro; Kajio, Hiroshi; Sugiyama, Takehiro	2017	10.1016/j.mayocp.2017.03.010		✓			
Association of soy food with cardiovascular outcomes and all-cause mortality in a Chinese population: a nationwide prospective cohort study.	Xue, Ting; Wen, Junping; Wan, Qin; Qin, Guijun; Yan, Li; Wang, Guixia; Qin, Yingfen; Luo, Zuojie; Tang, Xulei; Huo, Yanan; Hu, Ruying; Ye, Zhen; Shi, Lixin; Gao, Zhengnan; Su, Qing; Mu, Yiming; Zhao, Jiajun; Chen, Lulu; Zeng, Tianshu; Yu, Xuefeng; Li, Qiang; Shen, Feixia; Chen, Li; Zhang, Yinfei; Wang, Youmin; Deng, Huacong; Liu, Chao; Wu, Shengli; Yang, Tao; Li, Mian; Xu, Yu; Xu, Min; Wang, Tiange; Zhao, Zhiyun; Lu, Jieli; Bi, Yufang; Wang, Weiqing; Chen, Gang; Ning, Guang	2022	10.1007/s00394-021-02724-8		✓			

Diet synergies and mortality--a population-based case-control study of 32,462 Hong Kong Chinese older adults.	Schooling, C. Mary; Ho, Sai Yin; Leung, Gabriel M.; Thomas, G. Neil; McGhee, Sarah M.; Mak, Kwok Hang; Lam, Tai Hing	2006	10.1093/ije/dyi296		✓			
Retrospective cohort study of risk-factors for esophageal cancer in Linxian, People's Republic of China.	Yu, Y.; Taylor, P. R.; Li, J. Y.; Dawsey, S. M.; Wang, G. Q.; Guo, W. D.; Wang, W.; Liu, B. Q.; Blot, W. J.; Shen, Q.	1993	10.1007/BF00051313			✓	✓	
Effect of soy isoflavones on breast cancer recurrence and death for patients receiving adjuvant endocrine therapy.	Kang, Xinmei; Zhang, Qingyuan; Wang, Shuhuai; Huang, Xu; Jin, Shi	2010	10.1503/cmaj.091298	✓	✓			
Low-fat and high-fat dairy products are differently related to blood lipids and cardiovascular risk score.	Huo Yung Kai, Samantha; Bongard, Vanina; Simon, Chantal; Ruidavets, Jean-Bernard; Arveiler, Dominique; Dallongeville, Jean; Wagner, Aline; Amouyel, Philippe; Ferrières, Jean	2014	10.1177/2047487313503283		✓	✓	✓	
Theoretical substitutions between dairy products and all-cause and cause-specific mortality. Results from the Danish diet, cancer and health cohort.	Laursen, Anne Sofie D.; Thomsen, Anne L.; Beck, Anne; Overvad, Kim; Jakobsen, Marianne U.	2022	10.1017/S0007114521002464		✓		✓	
A traditional Sami diet score as a determinant of mortality in a general northern Swedish population.	Nilsson, Lena Maria; Winkvist, Anna; Brustad, Magritt; Jansson, Jan-Håkan; Johansson, Ingegerd; Lenner, Per; Lindahl, Bernt; Van Guelpen, Bethany	2012	10.3402/ijch.v71i0.18537		✓			
The effects of soy consumption before diagnosis on breast cancer survival: the Multiethnic Cohort Study.	Conroy, Shannon M.; Maskarinec, Gertraud; Park, Song-Yi; Wilkens, Lynne R.; Henderson, Brian E.; Kolonel, Laurence N.	2013	10.1080/01635581.2013.776694		✓			
Adherence to the healthy Nordic food index and total and cause-specific mortality among Swedish women.	Roswall, Nina; Sandin, Sven; Löf, Marie; Skeie, Guri; Olsen, Anja; Adami, Hans-Olov; Weiderpass, Elisabete	2015	10.1007/s10654-015-0021-x		✓			
Intake of coffee and tea and risk of ovarian cancer: a prospective cohort study.	Silvera, Stephanie A. N.; Jain, Meera; Howe, Geoffrey R.; Miller, Anthony B.; Rohan, Thomas E.	2007	10.1080/01635580701307945			✓		
Soy and Soy Products Intake, All-Cause Mortality, and Cause-Specific Mortality in Japan: The Jichi Medical School Cohort Study.	Yamasaki, Kyoko; Kayaba, Kazunori; Ishikawa, Shizukiyo	2015	10.1177/1010539514539545		✓			
Alcohol consumption and risk for coronary heart disease in men with healthy lifestyles.	Mukamal, Kenneth J.; Chiuve, Stephanie E.; Rimm, Eric B.	2006	10.1001/archinte.166.19.2145		✓	✓		

Dietary intake of flavonoids and oesophageal and gastric cancer: incidence and survival in the United States of America (USA).	Petrick, J. L.; Steck, S. E.; Bradshaw, P. T.; Trivers, K. F.; Abrahamson, P. E.; Engel, L. S.; He, K.; Chow, W.-H.; Mayne, S. T.; Risch, H. A.; Vaughan, T. L.; Gammon, M. D.	2015	10.1038/bjc.2015.25		✓			
Does diet matter for survival in long-lived cultures?	Wahlqvist, Mark L.; Darmadi-Blackberry, Irene; Kouris-Blazos, Antigone; Jolley, Damien; Steen, Bertil; Lukito, Widjaja; Horie, Yoshimitsu	2005			✓			
The Japanese food score and risk of all-cause, CVD and cancer mortality: the Japan Collaborative Cohort Study.	Okada, Emiko; Nakamura, Koshi; Ukawa, Shigekazu; Wakai, Kenji; Date, Chigusa; Iso, Hiroyasu; Tamakoshi, Akiko	2018	10.1017/S000711451800154X		✓			
Health-related behaviours as predictors of mortality and morbidity in Australian Aborigines.	Burke, V.; Zhao, Y.; Lee, A. H.; Hunter, E.; Spargo, R. M.; Gracey, M.; Smith, R. M.; Beilin, L. J.; Puddey, I. B.	2007	10.1016/j.ypmed.2006.09.008		✓			
Prospective study of educational background and stomach cancer in Japan.	Fujino, Yoshihisa; Tamakoshi, Akiko; Ohno, Yoshiyuki; Mizoue, Tetsuya; Tokui, Noritaka; Yoshimura, Takesumi	2002	10.1006/pmed.2002.1066		✓			
Association of Fermented Products with Risk of Cancer Recurrence and Mortality among Breast Cancer Survivors: A Prospective Cohort Study.	Yang, Jubin; Chung, Minsung; Park, Yongsoon	2023	10.1080/01635581.2023.2186259	✓				
Quantifying the benefits of Mediterranean diet in terms of survival.	Bellavia, Andrea; Tektonidis, Thanasis G.; Orsini, Nicola; Wolk, Alicja; Larsson, Susanna C.	2016	10.1007/s10654-016-0127-9		✓			
Intake of fermented and non-fermented dairy products and risk of incident CHD: the Kuopio Ischaemic Heart Disease Risk Factor Study.	Koskinen, Timo T.; Virtanen, Heli E. K.; Voutilainen, Sari; Tuomainen, Tomi-Pekka; Mursu, Jaakko; Virtanen, Jyrki K.	2018	10.1017/S0007114518002830		✓	✓		
Adherence to the 2015 Dietary Guidelines for Americans and mortality risk in a Mediterranean cohort: The SUN project.	Fresán, Ujué; Sabaté, Joan; Martínez-Gonzalez, Miguel A.; Segovia-Siapco, Gina; de la Fuente-Arrillaga, Carmen; Bes-Rastrollo, Maira	2019	10.1016/j.ypmed.2018.11.015		✓			
The Healthy Taiwanese Eating Approach is inversely associated with all-cause and cause-specific mortality: A prospective study on the Nutrition and Health Survey in Taiwan, 1993-1996.	Chuang, Shao-Yuan; Chang, Hsing-Yi; Fang, Hsin-Ling; Lee, Shu-Chen; Hsu, Yueh-Ying; Yeh, Wen-Ting; Liu, Wen-Ling; Pan, Wen-Harn	2021	10.1371/journal.pone.0251189		✓			

Coffee and tea consumption are associated with a lower incidence of chronic liver disease in the United States.	Ruhl, Constance E.; Everhart, James E.	2005	10.1053/j.gastro.2005.08.056			✓		
Diet and amyotrophic lateral sclerosis.	Morozova, Natalia; Weisskopf, Marc G.; McCullough, Marjorie L.; Munger, Kassandra L.; Calle, Eugenia E.; Thun, Michael J.; Ascherio, Alberto	2008	10.1097/EDE.0b013e3181632c5d		✓		✓	
Moderate coffee consumption is associated with lower risk of mortality in prior Acute Coronary Syndrome patients: a prospective analysis in the ERICO cohort.	Miranda, Andreia Machado; Goulart, Alessandra Carvalho; Benseñor, Isabela Martins; Lotufo, Paulo Andrade; Marchioni, Dirce Maria	2021	10.1080/09637486.2020.1862069	✓				
Factor analysis in the identification of dietary patterns and their predictive role in morbid and fatal events.	Menotti, Alessandro; Alberti-Fidanza, Adalberta; Fidanza, Flaminio; Lanti, Mariapaola; Fruttini, Daniela	2012	10.1017/S1368980011003235		✓			
Dietary habits and pancreatic cancer risk in a cohort of middle-aged and elderly Japanese.	Lin, Yingsong; Kikuchi, Shogo; Tamakoshi, Akiko; Yagyu, Kiyoko; Obata, Yuki; Inaba, Yutaka; Kurosawa, Michiko; Kawamura, Takashi; Motohashi, Yutaka; Ishibashi, Teruo	2006	10.1207/s15327914nc5601_6		✓			
Protein and amino acid intakes in relation to prostate cancer risk and mortality-A prospective study in the European Prospective Investigation into Cancer and Nutrition.	Schmidt, Julie A.; Huybrechts, Inge; Overvad, Kim; Eriksen, Anne Kirstine; Tjønneland, Anne; Kaaks, Rudolf; Katzke, Verena; Schulze, Matthias B.; Pala, Valeria; Sacerdote, Carlotta; Tumino, Rosario; Bueno-de-Mesquita, Bas; Sánchez, Maria-Jose; Huerta, José M.; Barricarte, Aurelio; Amiano, Pilar; Agudo, Antonio; Bjartell, Anders; Stocks, Tanja; Thysell, Elin; Wennberg, Maria; Weiderpass, Elisabete; Travis, Ruth C.; Key, Timothy J.; Perez-Cornago, Aurora	2023	10.1002/cam4.5289		✓			
The associations of dietary patterns with all-cause mortality and other lifestyle factors in the elderly: An age-specific prospective cohort study.	Zhao, Wenjing; Ukawa, Shigekazu; Okada, Emiko; Wakai, Kenji; Kawamura, Takashi; Ando, Masahiko; Tamakoshi, Akiko	2019	10.1016/j.clnu.2018.01.018		✓			
Low Protein Intake Irrespective of Source is Associated with Higher Mortality Among Older Community-dwelling Men.	Langsetmo, L.; Harrison, S.; Jonnalagadda, S.; Pereira, S. L.; Shikany, J. M.; Farsijani, S.; Lane, N. E.; Cauley, J. A.; Stone, K.; Cawthon, P. M.	2020	10.1007/s12603-020-1422-4		✓			
Geographical distribution of cerebrovascular disease mortality and food intakes in Japan.	Omura, T.; Hisamatsu, S.; Takizawa, Y.; Minowa, M.; Yanagawa, H.; Shigematsu, I.	1987	10.1016/0277-9536(87)90212-7				✓	

The association of obesity-related dietary patterns and main food groups derived by reduced-rank regression with cardiovascular diseases incidence and all-cause mortality: findings from 116,711 adults.	Maimaitiyiming, Maiwulamujiang; Yang, Hongxi; Li, Huiping; Xu, Chenjie; Li, Shu; Zhou, Lihui; Zhang, Xinyu; Wang, Yaogang	2023	10.1007/s00394-023-03177-x		✓			
Dietary patterns and mortality in Danish men and women: a prospective observational study.	Osler, M.; Heitmann, B. L.; Gerdes, L. U.; Jørgensen, L. M.; Schroll, M.	2001	10.1079/bjn2000240		✓			
Coffee consumption and risk of cardiovascular events after acute myocardial infarction: results from the GISSI (Gruppo Italiano per lo Studio della Sopravvivenza nell'Infarto miocardico)-Prevenzione trial.	Silletta, Maria Giuseppina; Marfisi, RosaMaria; Levantesi, Giacomo; Boccanelli, Alessandro; Chieffo, Carmelo; Franzosi, MariaGrazia; Geraci, Enrico; Maggioni, Aldo Pietro; Nicolosi, Gianluigi; Schweiger, Carlo; Tavazzi, Luigi; Tognoni, Gianni; Marchioli, Roberto	2007	10.1161/CIRCULATIONAHA.107.712976	✓				
Red meat, processed meat, and other dietary protein sources and risk of overall and cause-specific mortality in The Netherlands Cohort Study.	van den Brandt, Piet A.	2019	10.1007/s10654-019-00483-9		✓			
Whole-grain products and whole-grain types are associated with lower all-cause and cause-specific mortality in the Scandinavian HELGA cohort.	Johnsen, Nina F.; Frederiksen, Kirsten; Christensen, Jane; Skeie, Guri; Lund, Eiliv; Landberg, Rikard; Johansson, Ingegerd; Nilsson, Lena M.; Halkjær, Jytte; Olsen, Anja; Overvad, Kim; Tjønneland, Anne	2015	10.1017/S0007114515001701		✓			
Association of dietary insulinemic and inflammatory potential with risk of liver cancer and chronic liver disease mortality in postmenopausal women: a prospective cohort study.	Zhang, Xinyuan; Zhao, Longgang; Christopher, Cami N.; Tabung, Fred K.; Bao, Wei; Garcia, David O.; Shadyab, Aladdin H.; Saquib, Nazmus; Neuhouser, Marian L.; Tinker, Lesley F.; Zhang, Xuehong	2023	10.1016/j.ajcnut.2023.07.009		✓			
The Japanese Dietary Pattern Is Associated with Longer Disability-Free Survival Time in the General Elderly Population in the Ohsaki Cohort 2006 Study.	Zhang, Shu; Tomata, Yasutake; Sugawara, Yumi; Tsuduki, Tsuyoshi; Tsuji, Ichiro	2019	10.1093/jn/nxz051		✓			
Dietary patterns and 15-y risks of major coronary events, diabetes, and mortality.	Brunner, Eric J.; Mosdøl, Annhild; Witte, Daniel R.; Martikainen, Pekka; Stafford, Mai; Shipley, Martin J.; Marmot, Michael G.	2008	10.1093/ajcn/87.5.1414		✓			

Vegetarianism, dietary fiber, and mortality.	Burr, M. L.; Sweetnam, P. M.	1982	10.1093/ajcn/36.5.873		✓			
Reduced mortality among whole grain bread eaters in men and women in the Norwegian County Study.	Jacobs, D. R. Jr; Meyer, H. E.; Solvoll, K.	2001	10.1038/sj.ejcn.1601133		✓			
Carbohydrate nutrition and inflammatory disease mortality in older adults.	Buyken, Anette E.; Flood, Victoria; Empson, Marianne; Rochtchina, Elena; Barclay, Alan W.; Brand-Miller, Jennie; Mitchell, Paul	2010	10.3945/ajcn.2010.29390		✓			
Coffee and coronary heart disease.	Myers, M. G.; Basinski, A.	1992	10.1001/archinte.152.9.1767				✓	
Associations between diet and cancer, ischemic heart disease, and all-cause mortality in non-Hispanic white California Seventh-day Adventists.	Fraser, G. E.	1999	10.1093/ajcn/70.3.532s		✓	✓		
A food pattern predicting prospective weight change is associated with risk of fatal but not with nonfatal cardiovascular disease.	Drogan, Dagmar; Hoffmann, Kurt; Schulz, Mandy; Bergmann, Manuela M.; Boeing, Heiner; Weikert, Cornelia	2007	10.1093/jn/137.8.1961	✓	✓	✓	✓	
Dietary patterns and all-cause, cancer, and cardiovascular disease mortality in Japanese men and women: The Japan public health center-based prospective study.	Nanri, Akiko; Mizoue, Tetsuya; Shimazu, Taichi; Ishihara, Junko; Takachi, Ribeka; Noda, Mitsuhiko; Iso, Hiroyasu; Sasazuki, Shizuka; Sawada, Norie; Tsugane, Shoichiro	2017	10.1371/journal.pone.0174848		✓			
Intakes of 4 dietary lignans and cause-specific and all-cause mortality in the Zutphen Elderly Study.	Milder, Ivon E. J.; Feskens, Edith J. M.; Arts, Ilja C. W.; Bueno-de-Mesquita, H. Bas; Hollman, Peter C. H.; Kromhout, Daan	2006	10.1093/ajcn/84.1.400		✓			
Dietary patterns, subclinical inflammation, incident coronary heart disease and mortality in middle-aged men from the MONICA/KORA Augsburg cohort study.	Meyer, J.; Döring, A.; Herder, C.; Roden, M.; Koenig, W.; Thorand, B.	2011	10.1038/ejcn.2011.37	✓	✓	✓	✓	
Food intake patterns and risk of coronary heart disease: a prospective cohort study examining the use of traditional scoring techniques.	Osler, M.; Helms Andreasen, A.; Heitmann, B.; Høidrup, S.; Gerdes, U.; Mørch Jørgensen, L.; Schroll, M.	2002	10.1038/sj.ejcn.1601360		✓			

Healthy aspects of the Nordic diet are related to lower total mortality.	Olsen, Anja; Egeberg, Rikke; Halkjær, Jytte; Christensen, Jane; Overvad, Kim; Tjønneland, Anne	2011	10.3945/jn.110.131375		✓			
Lifestyle and mortality among Norwegian men.	Rotevatn, S.; Akslen, L. A.; Bjelke, E.	1989	10.1016/0091-7435(89)90003-0				✓	
Food choices and coronary heart disease: a population based cohort study of rural Swedish men with 12 years of follow-up.	Holmberg, Sara; Thelin, Anders; Stiernström, Eva-Lena	2009	10.3390/ijerph6102626		✓			
Effect of food intake pattern on all-cause mortality in the community elderly: a 7-year longitudinal study.	Kumagai, S.; Shibata, H.; Watanabe, S.; Suzuki, T.; Haga, H.	1999			✓			
Dietary vitamin K intake in relation to cancer incidence and mortality: results from the Heidelberg cohort of the European Prospective Investigation into Cancer and Nutrition (EPIC-Heidelberg).	Nimptsch, Katharina; Rohrmann, Sabine; Kaaks, Rudolf; Linseisen, Jakob	2010	10.3945/ajcn.2009.28691		✓			
Dietary patterns and survival in older Dutch women.	Waijers, Patricia M. C. M.; Ocké, Marga C.; van Rossum, Caroline T. M.; Peeters, Petra H. M.; Bamia, Christina; Chloptsios, Yiannis; van der Schouw, Yvonne T.; Slimani, Nadia; Bueno-de-Mesquita, H. Bas	2006	10.1093/ajcn/83.5.1170		✓			
Determinants of All-Cause Mortality and Incidence of Cardiovascular Disease (2009 to 2013) in Older Adults: The Ikaria Study of the Blue Zones.	Chrysohoou, Christina; Pitsavos, Christos; Lazaros, George; Skoumas, John; Tousoulis, Dimitris; Stefanadis, Christodoulos	2016	10.1177/0003319715603185		✓			
Dietary habits and risk of urothelial cancer death in a large-scale cohort study (JACC Study) in Japan.	Sakauchi, Fumio; Mori, Mitsuru; Washio, Masakazu; Watanabe, Yoshiyuki; Ozasa, Kotaro; Hayashi, Kyohei; Miki, Tsuneharu; Nakao, Masahiro; Mikami, Kazuya; Ito, Yoshinori; Wakai, Kenji; Tamakoshi, Akiko	2004	10.1207/s15327914nc5001_5			✓		
Food intake patterns, self rated health and mortality in Danish men and women. A prospective observational study.	Osler, M.; Heitmann, B. L.; Høidrup, S.; Jørgensen, L. M.; Schroll, M.	2001	10.1136/jech.55.6.399		✓			
Cancer and total mortality among active Mormons.	Enstrom, J. E.	1978	10.1002/1097-0142(197810)42:4<1943::aid-cncr2820420437>3.0.co;2-l		✓			

Dietary habits and mortality in 11,000 vegetarians and health conscious people: results of a 17 year follow up.	Key, T. J.; Thorogood, M.; Appleby, P. N.; Burr, M. L.	1996	10.1136/bmj.313.7060.775		✓			
Patterns of artificial sweetener use and weight change in an American Cancer Society prospective study.	Stellman, S. D.; Garfinkel, L.	1988	10.1016/s0195-6663(88)80051-5		✓			
Dietary pattern and 20 year mortality in elderly men in Finland, Italy, and The Netherlands: longitudinal cohort study.	Huijbregts, P.; Feskens, E.; Räsänen, L.; Fidanza, F.; Nissinen, A.; Menotti, A.; Kromhout, D.	1997	10.1136/bmj.315.7099.13		✓			
The relation between dietary flavonol intake and coronary heart disease mortality: a meta-analysis of prospective cohort studies.	Huxley, R. R.; Neil, H. A. W.	2003	10.1038/sj.ejcn.1601624		✓			
Comparison of two statistical approaches to predict all-cause mortality by dietary patterns in German elderly subjects.	Hoffmann, Kurt; Boeing, Heiner; Boffetta, Paolo; Nagel, Gabriele; Orfanos, Philippos; Ferrari, Pietro; Bamia, Christina	2005	10.1079/bjn20051399		✓			
Walking four times weekly for at least 15 min is associated with longevity in a cohort of very elderly people.	Fortes, Cristina; Mastroeni, Simona; Sperati, Alessandra; Pacifici, Roberta; Zuccaro, PierGiorgio; Francesco, Forastiere; Agabiti, Nerina; Piras, Giovanna; Amleto, D'Amicis; Ebrahim, Shah	2013	10.1016/j.maturitas.2012.12.001		✓			
Vitamin E and coronary heart disease: the European paradox.	Bellizzi, M. C.; Franklin, M. F.; Duthie, G. G.; James, W. P.	1994			✓	✓		
What was the lifestyle of people who died by cardiovascular diseases?	Kern, Josipa; Ivankovic, Davor; Sogoric, Selma; Vuletic, Silvije	2004		✓				
Green tea (Camellia sinensis) for the prevention of cancer.	Filippini, Tommaso; Malavolti, Marcella; Borrelli, Francesca; Izzo, Angelo A.; Fairweather-Tait, Susan J.; Horneber, Markus; Vinceti, Marco	2020	10.1002/14651858.CD005004.pub3		✓			
The Mediterranean diet in relation to mortality and CVD: a Danish cohort study.	Tognon, Gianluca; Lissner, Lauren; Sæbye, Ditte; Walker, Karen Z.; Heitmann, Berit L.	2014	10.1017/S0007114513001931		✓			
Associations of dietary protein with disease and mortality in a prospective study of postmenopausal women.	Kelemen, Linda E.; Kushi, Lawrence H.; Jacobs, David R. Jr; Cerhan, James R.	2005	10.1093/aje/kwi038		✓			

Role of smoking and diet in the cross-cultural variation in lung-cancer mortality: the Seven Countries Study. Seven Countries Study Research Group.	Mulder, I.; Jansen, M. C.; Smit, H. A.; Jacobs, D. R. Jr; Menotti, A.; Nissinen, A.; Fidanza, F.; Kromhout, D.	2000	10.1002/1097-0215(20001115)88:4<665::aid-ijc23>3.0.co;2-q		✓			
A dietary pattern rich in olive oil and raw vegetables is associated with lower mortality in Italian elderly subjects.	Masala, Giovanna; Ceroti, Marco; Pala, Valeria; Krogh, Vittorio; Vineis, Paolo; Sacerdote, Carlotta; Saieva, Calogero; Salvini, Simonetta; Sieri, Sabina; Berrino, Franco; Panico, Salvatore; Mattiello, Amalia; Tumino, Rosario; Giurdanella, Maria C.; Bamia, Christina; Trichopoulou, Antonia; Riboli, Elio; Palli, Domenico	2007	10.1017/S0007114507704981		✓			
Diet, tobacco use, and fatal prostate cancer: results from the Lutheran Brotherhood Cohort Study.	Hsing, A. W.; McLaughlin, J. K.; Schuman, L. M.; Bjelke, E.; Gridley, G.; Wacholder, S.; Chien, H. T.; Blot, W. J.	1990			✓			
High nutrition risk related to dietary intake is associated with an increased risk of hospitalisation and mortality for older Māori: LiLACS NZ.	North, Sylvia M.; Wham, Carol A.; Teh, Ruth; Moyes, Simon A.; Rolleston, Anna; Kerse, Ngaire	2018	10.1111/1753-6405.12793		✓			
Serum adiponectin multimer complexes and liver cancer risk in a large cohort study in Japan.	Kotani, Kazuhiko; Wakai, Kenji; Shibata, Akira; Fujita, Yuki; Ogimoto, Itsuro; Naito, Mariko; Kurozawa, Yoichi; Suzuki, Hiroshi; Yoshimura, Takesumi; Tamakoshi, Akiko	2009		✓		✓	✓	
Major dietary protein sources and risk of coronary heart disease in women.	Bernstein, Adam M.; Sun, Qi; Hu, Frank B.; Stampfer, Meir J.; Manson, JoAnn E.; Willett, Walter C.	2010	10.1161/CIRCULATIONAHA.109.915165		✓			
Coffee consumption and the incidence of coronary heart disease.	LaCroix, A. Z.; Mead, L. A.; Liang, K. Y.; Thomas, C. B.; Pearson, T. A.	1986	10.1056/NEJM198610163151601			✓		
Coffee and alcohol consumption as triggering factors for sudden cardiac death: case-crossover study.	Selb Semerl, Jozica; Selb, Kristina	2004		✓			✓	
Impact of Soy Foods on the Development of Breast Cancer and the Prognosis of Breast Cancer Patients.	Messina, Mark	2016	10.1159/000444735		✓		✓	
Processed meat: the real villain?	Rohrmann, Sabine; Linseisen, Jakob	2016	10.1017/S0029665115004255		✓			

Age at first birth, dietary practices and breast cancer mortality in various Italian regions.	La Vecchia, C.; Pampallona, S.	1986	10.1159/000226094		✓			
Coffee, coronary heart disease and mortality in middle-aged Swedish men: findings from the Primary Prevention Study.	Rosengren, A.; Wilhelmsen, L.	1991	10.1111/j.1365-2796.1991.tb00407.x				✓	
Gastric cancer in Italy.	Cipriani, F.; Buiatti, E.; Palli, D.	1991	10.1007/bf01297098				✓	
Potential health hazards of eating red meat.	Wolk, A.	2017	10.1111/joim.12543		✓			
Public health impact of daily life triggers of sudden cardiac death: A systematic review and comparative risk assessment.	Čulić, Viktor; AlTurki, Ahmed; Proietti, Riccardo	2021	10.1016/j.resuscitation.2021.02.036				✓	
Risk factors for small intestine cancer.	Chow, W. H.; Linet, M. S.; McLaughlin, J. K.; Hsing, A. W.; Chien, H. T.; Blot, W. J.	1993	10.1007/BF00053158	✓	✓		✓	
Association of all-cause mortality with sugar intake from different sources in the prospective cohort of UK Biobank participants.	Kaiser, Anna; Schaefer, Sylva M.; Behrendt, Inken; Eichner, Gerrit; Fasshauer, Mathias	2023	10.1017/S0007114522003233		✓			
Is Butter Back? A Systematic Review and Meta-Analysis of Butter Consumption and Risk of Cardiovascular Disease, Diabetes, and Total Mortality.	Pimpin, Laura; Wu, Jason H. Y.; Haskelberg, Hila; Del Gobbo, Liana; Mozaffarian, Dariush	2016	10.1371/journal.pone.0158118		✓			
Protein and legume intake and prostate cancer mortality in Puerto Rican men.	Smit, Ellen; Garcia-Palmieri, Mario R.; Figueroa, Nayda R.; McGee, Daniel L.; Messina, Mark; Freudenheim, Jo L.; Crespo, Carlos J.	2007	10.1080/01635580701328206		✓			
Optimal dairy intake is predicated on total, cardiovascular, and stroke mortalities in a Taiwanese cohort.	Huang, Lin-Yuan; Wahlqvist, Mark L.; Huang, Yi-Chen; Lee, Meei-Shyuan	2014	10.1080/07315724.2013.875328	✓	✓			
The Associations of Fruit and Vegetable Intakes with Burden of Diseases: A Systematic Review of Meta-Analyses.	Yip, Cynthia Sau Chun; Chan, Wendy; Fielding, Richard	2019	10.1016/j.jand.2018.11.007		✓			

'Mediterranean' dietary pattern for the primary prevention of cardiovascular disease.	Rees, Karen; Hartley, Louise; Flowers, Nadine; Clarke, Aileen; Hooper, Lee; Thorogood, Margaret; Stranges, Saverio	2013	10.1002/14651858.CD009825.pub2		✓	✓	✓	
Coffee consumption and incidence of heart failure in women.	Levitan, Emily B.; Ahmed, Hanna N.; Mittleman, Murray A.; Wolk, Alicja	2011	10.1161/CIRCHEARTFAILURE.111.960898			✓		
Impact of Coffee Consumption on Physiological Markers of Cardiovascular Risk: A Systematic Review.	Daneschvar, Homayoun L.; Smetana, Gerald W.; Brindamour, Luke; Bain, Paul A.; Mukamal, Kenneth J.	2021	10.1016/j.amjmed.2020.09.036			✓	✓	
Coffee consumption and risk of colorectal cancer.	Bidel, S.; Hu, G.; Jousilahti, P.; Antikainen, R.; Pukkala, E.; Hakulinen, T.; Tuomilehto, J.	2010	10.1038/ejcn.2010.103			✓		
Dietary factors and stomach cancer mortality.	Ngoan, L. T.; Mizoue, T.; Fujino, Y.; Tokui, N.; Yoshimura, T.	2002	10.1038/sj.bjc.6600415			✓		
Risk factors for major gastrointestinal bleeding in the general population in Finland.	Vora, Pareen; Herrera, Ronald; Pietila, Arto; Mansmann, Ulrich; Brobert, Gunnar; Peltonen, Markku; Salomaa, Veikko	2022	10.3748/wjg.v28.i18.2008			✓		
Change in the association between coffee intake and ischemic heart disease in an international ecological study from 1990 to 2018.	Shirai, Yoshiro; Imai, Tomoko; Sezaki, Ayako; Miyamoto, Keiko; Kawase, Fumiya; Abe, Chisato; Sanada, Masayo; Inden, Ayaka; Kato, Takumi; Suzuki-Sugihara, Norie; Shimokata, Hiroshi	2022	10.1038/s41598-022-15611-x				✓	
Association of a diabetes risk score with risk of myocardial infarction, stroke, specific types of cancer, and mortality: a prospective study in the European Prospective Investigation into Cancer and Nutrition (EPIC)-Potsdam cohort.	Heidemann, Christin; Boeing, Heiner; Pischon, Tobias; Nöthlings, Ute; Joost, Hans-Georg; Schulze, Matthias B.	2009	10.1007/s10654-009-9338-7		✓			
The association between lifestyle factors and Parkinson's disease progression and mortality.	Paul, Kimberly C.; Chuang, Yu-Hsuan; Shih, I.-Fan; Keener, Adrienne; Bordelon, Yvette; Bronstein, Jeff M.; Ritz, Beate	2019	10.1002/mds.27577	✓				
Inverse association of tea and flavonoid intakes with incident myocardial infarction: the Rotterdam Study.	Geleijnse, Johanna M.; Launer, Lenore J.; Van der Kuip, Deirdre A. M.; Hofman, Albert; Witteman, Jacqueline C. M.	2002	10.1093/ajcn/75.5.880		✓			

Cancer mortality correlation studies. II. Regional associations of mortalities with the consumptions of foods and other commodities.	Schrauzer, G. N.	1976	10.1016/0306-9877(76)90060-8		✓			
Serum cholesterol in young men and subsequent cardiovascular disease.	Klag, M. J.; Ford, D. E.; Mead, L. A.; He, J.; Whelton, P. K.; Liang, K. Y.; Levine, D. M.	1993	10.1056/NEJM199302043280504		✓			
BMI Is a Risk Factor for Colorectal Cancer Mortality.	Shaukat, Aasma; Dostal, Allison; Menk, Jeremiah; Church, Timothy R.	2017	10.1007/s10620-017-4682-z		✓			
Mediterranean-style diet for the primary and secondary prevention of cardiovascular disease.	Rees, Karen; Takeda, Andrea; Martin, Nicole; Ellis, Leila; Wijesekara, Dilini; Vepa, Abhinav; Das, Archik; Hartley, Louise; Stranges, Saverio	2019	10.1002/14651858.CD009825.pub3		✓			
The Influence of Nutritional and Lifestyle Factors on Glioma Incidence.	Bielecka, Joanna; Markiewicz-Żukowska, Renata	2020	10.3390/nu12061812			✓	✓	
Male longevity in Sardinia, a review of historical sources supporting a causal link with dietary factors.	Pes, G. M.; Tolu, F.; Dore, M. P.; Sechi, G. P.; Errigo, A.; Canelada, A.; Poulain, M.	2015	10.1038/ejcn.2014.230			✓		
Substitution of sugar-sweetened beverages for other beverages and the risk of developing coronary heart disease: Results from the Harvard Pooling Project of Diet and Coronary Disease.	Keller, Amélie; O'Reilly, Eilis J.; Malik, Vasanti; Buring, Julie E.; Andersen, Ingelise; Steffen, Lyn; Robien, Kim; Männistö, Satu; Rimm, Eric B.; Willett, Walter; Heitmann, Berit Lilienthal	2020	10.1016/j.ypmed.2019.105970			✓		
Coffee consumption, health benefits and side effects: a narrative review and update for dietitians and nutritionists.	Barrea, Luigi; Pugliese, Gabriella; Frias-Toral, Evelyn; El Ghoch, Marwan; Castellucci, Bianca; Chapela, Sebastián Pablo; Carignano, María de Los Angeles; Laudisio, Daniela; Savastano, Silvia; Colao, Annamaria; Muscogiuri, Giovanna	2023	10.1080/10408398.2021.1963207				✓	
Systematic Review on Polyphenol Intake and Health Outcomes: Is there Sufficient Evidence to Define a Health-Promoting Polyphenol-Rich Dietary Pattern?	Del Bo', Cristian; Bernardi, Stefano; Marino, Mirko; Porrini, Marisa; Tucci, Massimiliano; Guglielmetti, Simone; Cherubini, Antonio; Carrieri, Barbara; Kirkup, Benjamin; Kroon, Paul; Zamora-Ros, Raul; Liberona, Nicole Hidalgo; Andres-Lacueva, Cristina; Riso, Patrizia	2019	10.3390/nu11061355		✓			
Coffee consumption is not associated with prevalent subclinical cardiovascular disease (CVD) or the risk of CVD events, in nonalcoholic fatty liver disease: results	Simon, Tracey G.; Trejo, Maria Esther Perez; Zeb, Irfan; Frazier-Wood, Alexis C.; McClelland, Robyn L.; Chung, Raymond T.; Budoff, Matthew J.	2017	10.1016/j.metabol.2017.06.007	✓		✓		

from the multi-ethnic study of atherosclerosis.								
Antioxidant flavonols and ischemic heart disease in a Welsh population of men: the Caerphilly Study.	Hertog, M. G.; Sweetnam, P. M.; Fehily, A. M.; Elwood, P. C.; Kromhout, D.	1997	10.1093/ajcn/65.5.1489		✓			
Association of chocolate consumption with neurological and cardiovascular outcomes in atrial fibrillation: data from two Swiss atrial fibrillation cohort studies (Swiss-AF and BEAT-AF).	Stauber, Annina; Müller, Andreas; Rommers, Nikki; Aeschbacher, Stefanie; Rodondi, Nicolas; Bonati, Leo H.; Beer, Juerg H.; Jeger, Raban V.; Kurz, David J.; Liedtke, Claudia; Ammann, Peter; Di Valentino, Marcello; Chocano, Patricia; Kobza, Richard; Kühne, Michael; Conen, David; Osswald, Stefan; Bernheim, Alain M.	2023	10.57187/smw.2023.40109	✓				
Coffee Consumption and Cardiovascular Disease: A Condensed Review of Epidemiological Evidence and Mechanisms.	Rodríguez-Artalejo, Fernando; López-García, Esther	2018	10.1021/acs.jafc.7b04506				✓	
The effect of coffee on blood lipids and blood pressure. Results from a Norwegian cross-sectional study, men and women, 40-42 years.	Stensvold, I.; Tverdal, A.; Foss, O. P.	1989	10.1016/0895-4356(89)90101-7			✓	✓	
Dairy and Cardiovascular Disease: A Review of Recent Observational Research.	Rice, Beth H.	2014	10.1007/s13668-014-0076-4				✓	
Chocolate intake and incidence of heart failure: Findings from the Cohort of Swedish Men.	Steinhaus, Daniel A.; Mostofsky, Elizabeth; Levitan, Emily B.; Dorans, Kirsten S.; Håkansson, Niclas; Wolk, Alicja; Mittleman, Murray A.	2017	10.1016/j.ahj.2016.10.002			✓		
Coffee consumption and risk of stroke in women.	Larsson, Susanna C.; Virtamo, Jarmo; Wolk, Alicja	2011	10.1161/STROKEAHA.110.603787			✓		
Chocolate intake and incidence of heart failure: a population-based prospective study of middle-aged and elderly women.	Mostofsky, Elizabeth; Levitan, Emily B.; Wolk, Alicja; Mittleman, Murray A.	2010	10.1161/CIRCHEARTFAILURE.110.944025			✓		
Health Effects of Coffee: Mechanism Unraveled?	Kolb, Hubert; Kempf, Kerstin; Martin, Stephan	2020	10.3390/nu12061842				✓	

Factors associated with reaching 90 years of age: a study of men born in 1913 in Gothenburg, Sweden.	Wilhelmsen, L.; Svärdsudd, K.; Eriksson, H.; Rosengren, A.; Hansson, P.-O.; Welin, C.; Odén, A.; Welin, L.	2011	10.1111/j.1365-2796.2010.02331.x			✓		
Association between dietary nutrient composition and the incidence of cirrhosis or liver cancer in the United States population.	Ioannou, George N.; Morrow, Olivia B.; Connole, Marah L.; Lee, Sum P.	2009	10.1002/hep.22941		✓	✓		
Coffee intake in midlife and risk of dementia and its neuropathologic correlates.	Gelber, Rebecca P.; Petrovitch, Helen; Masaki, Kamal H.; Ross, G. Webster; White, Lon R.	2011	10.3233/JAD-2010-101428			✓	✓	
Factor analysis of digestive cancer mortality and food consumption in 65 Chinese counties.	Zhuo, X. G.; Watanabe, S.	1999	10.2188/jea.9.275			✓		
A large scale cohort study on cancer risks by diet--with special reference to the risk reducing effects of green-yellow vegetable consumption.	Hirayama, T.	1985		✓	✓	✓	✓	
Diet and Alzheimer's disease risk factors or prevention: the current evidence.	Solfrizzi, Vincenzo; Panza, Francesco; Frisardi, Vincenza; Seripa, Davide; Logroscino, Giancarlo; Imbimbo, Bruno P.; Pilotto, Alberto	2011	10.1586/ern.11.56		✓		✓	
Coffee, alcohol and risk of coronary heart disease among Japanese men living in Hawaii.	Yano, K.; Rhoads, G. G.; Kagan, A.	1977	10.1056/NEJM197708252970801			✓		
Fish sauce and gastric cancer:an ecological study in Fujian Province,China.	Cai, Lin; Yu, Shun-Zhang; Ye, Wei-Min; Yi, Ying-Nan	2000	10.3748/wjg.v6.i5.671		✓			
Coffee consumption and risk of prostate cancer: an up-to-date meta-analysis.	Zhong, S.; Chen, W.; Yu, X.; Chen, Z.; Hu, Q.; Zhao, J.	2014	10.1038/ejcn.2013.256			✓		
Smoking habits, sales of fat and antihypertensives fail to explain the high coronary mortality in cold regions of Sweden.	Gyllerup, S.; Lanke, J.; Lindholm, L. H.; Schersten, B.	1991	10.1177/003693309103600602		✓			
Coffee consumption and urologic cancer risk: a meta-analysis of cohort studies.	Huang, Tian-bao; Guo, Zhui-feng; Zhang, Xiao-long; Zhang, Xiao-peng; Liu, Huan; Geng, Jiang; Yao, Xu-dong; Zheng, Jun-hua	2014	10.1007/s11255-014-0699-9			✓		

Thrombosis preventive potential of chicory coffee consumption: a clinical study.	Schumacher, Edit; Vigh, Eva; Molnár, Valéria; Kenyeres, Péter; Fehér, Gergely; Késmárky, Gábor; Tóth, Kálmán; Garai, János	2011	10.1002/ptr.3481				✓	
Coffee and tea on cardiovascular disease (CVD) prevention.	Chieng, David; Kistler, Peter M.	2022	10.1016/j.tcm.2021.08.004				✓	
Saturated Fats and Health: A Reassessment and Proposal for Food-Based Recommendations: JACC State-of-the-Art Review.	Astrup, Arne; Magkos, Faidon; Bier, Dennis M.; Brenna, J. Thomas; de Oliveira Otto, Marcia C.; Hill, James O.; King, Janet C.; Mente, Andrew; Ordovas, Jose M.; Volek, Jeff S.; Yusuf, Salim; Krauss, Ronald M.	2020	10.1016/j.jacc.2020.05.077				✓	
Red Yeast Rice Plus Berberine: Practical Strategy for Promoting Vascular and Metabolic Health.	McCarty, Mark F.; O'Keefe, James H.; DiNicolantonio, James J.	2015				✓	✓	
Navigating the maize between red meat and oncomirs.	Thompson, Patricia A.	2014	10.1158/1940-6207.CAPR-14-0196	✓	✓	✓	✓	
Association Between Water Intake and Mortality Risk-Evidence From a National Prospective Study.	Zhou, Hao-Long; Wei, Mu-Hong; Cui, Yuan; Di, Dong-Sheng; Song, Wen-Jing; Zhang, Ru-Yi; Liu, Jun-An; Wang, Qi	2022	10.3389/fnut.2022.822119		✓			
The association between coffee consumption and risk of incident depression and anxiety: Exploring the benefits of moderate intake.	Min, Jiahao; Cao, Zhi; Cui, Linlin; Li, Feimeng; Lu, Zuolin; Hou, Yabing; Yang, Hongxi; Wang, Xiaohe; Xu, Chenjie	2023	10.1016/j.psychres.2023.115307			✓		
Mortality reduction among persons with type 2 diabetes: (-)-Epicatechin as add-on therapy to metformin?	Moreno-Ulloa, Aldo; Moreno-Ulloa, Javier	2016	10.1016/j.mehy.2016.04.018		✓	✓	✓	
Chocolate and prevention of cardiovascular disease: a systematic review.	Ding, Eric L.; Hutfless, Susan M.; Ding, Xin; Girotra, Saket	2006	10.1186/1743-7075-3-2			✓	✓	
Coffee consumption and cardiovascular health: getting to the heart of the matter.	Rebello, Salome A.; van Dam, Rob M.	2013	10.1007/s11886-013-0403-1			✓	✓	
Cooking oil/fat consumption and deaths from cardiometabolic diseases and other causes: prospective analysis of 521,120 individuals.	Zhang, Yu; Zhuang, Pan; Wu, Fei; He, Wei; Mao, Lei; Jia, Wei; Zhang, Yiju; Chen, Xiaoqian; Jiao, Jingjing	2021	10.1186/s12916-021-01961-2		✓			

Recent advances in clinical practice: colorectal cancer chemoprevention in the average-risk population.	Chapelle, Nicolas; Martel, Myriam; Toes-Zoutendijk, Esther; Barkun, Alan N.; Bardou, Marc	2020	10.1136/gutjnl-2020-320990			✓		
Major cardiovascular disease (CVD) risk factors in midlife and extreme longevity.	Urtamo, Annele; Jyväkorpi, Satu K.; Kautiainen, Hannu; Pitkälä, Kaisu H.; Strandberg, Timo E.	2020	10.1007/s40520-019-01364-7		✓			
Cereal grains and coronary heart disease.	Truswell, A. S.	2002	10.1038/sj.ejcn.1601283				✓	
Effects of high flavanol dark chocolate on cardiovascular function and platelet aggregation.	Rull, Gurvinder; Mohd-Zain, Zetty N.; Shiel, Julian; Lundberg, Martina H.; Collier, David J.; Johnston, Atholl; Warner, Timothy D.; Corder, Roger	2015	10.1016/j.vph.2015.02.010			✓	✓	
Effects of low habitual cocoa intake on blood pressure and bioactive nitric oxide: a randomized controlled trial.	Taubert, Dirk; Roesen, Renate; Lehmann, Clara; Jung, Norma; Schömig, Edgar	2007	10.1001/jama.298.1.49			✓	✓	
Caffeine and coffee: effects on health and cardiovascular disease.	Chou, T. M.; Benowitz, N. L.	1994	10.1016/1367-8280(94)90130-9			✓	✓	
Coffee intake and death from coronary heart disease. Coffee may have both short and long term effects.	Marchioli, R.; Di Mascio, R.; Marfisi, R. M.; Vitullo, F.; Tognoni, G.	1996	10.1136/bmj.312.7045.1539			✓		
Cancer protective properties of cocoa: a review of the epidemiologic evidence.	Maskarinec, Gertraud	2009	10.1080/01635580902825662			✓	✓	
Lifestyle and chronic kidney disease: A machine learning modeling study.	Luo, Wenjin; Gong, Lilin; Chen, Xiangjun; Gao, Rufei; Peng, Bin; Wang, Yue; Luo, Ting; Yang, Yi; Kang, Bing; Peng, Chuan; Ma, Linqiang; Mei, Mei; Liu, Zhiping; Li, Qifu; Yang, Shumin; Wang, Zhihong; Hu, Jinbo	2022	10.3389/fnut.2022.918576			✓		
The PURE study and the enigmatic aspects of the diet: is it possible that an high saturated fat consumption would not be harmful?	Poli, Andrea	2020	10.1093/eurheartj/suaa074				✓	
Prevention of Cardiovascular Disease With Med or Veg Diets	NCT02641834,	2015		✓	✓	✓	✓	
Milk, yogurt, cheese and bone: friends or foes?	Iuliano, S	2020	10.1007/s00198-020-05692-7	✓	✓	✓	✓	

Milk and Soured Milk (filmjölk) Intervention on Oxidative Stress and Inflammation	NCT03821116,	2019		✓	✓	✓	✓	
The scale of the evidence base on the health effects of conventional yogurt consumption: findings of a scoping review	Glanville, JM; Brown, S; Shamir, R; Szajewska, H; Eales, JF	2015	10.3389/fphar.2015.00246			✓		
Dark chocolate intake buffers stress reactivity in humans	Wirtz, PH; von Känel, R; Meister, RE; Arpagaus, A; Treichler, S; Kuebler, U; Huber, S; Ehlert, U	2014	10.1016/j.jacc.2014.02.580			✓	✓	
Age-dependent Effects of Flavanols on Vascular Status	NCT01639781,	2012		✓	✓	✓	✓	
Effects of Phenolic Acids on Endothelial Function	NCT01772784,	2012		✓	✓	✓	✓	
Cocoa Ileostomy Study	NCT03765606,	2018		✓	✓	✓	✓	
Association of Sugar-Sweetened, Artificially Sweetened, and Unsweetened Coffee Consumption with All-Cause and Cause-Specific Mortality	Liu, D.; Li, Z.-H.; Shen, D.; Zhang, P.-D.; Song, W.-Q.; Zhang, W.-T.; Huang, Q.-M.; Chen, P.-L.; Zhang, X.-R.; Mao, C.	2023	10.7326/L22-0368	✓	✓	✓	✓	
Meal timing of dietary total antioxidant capacity and its association with all-cause, CVD and cancer mortality: the US national health and nutrition examination survey, 1999–2018	Wang, P.; Jiang, X.; Tan, Q.; Du, S.; Shi, D.	2023	10.1186/s12966-023-01487-1		✓			
We Are What We Eat: The Association Between Ultra-Processed Food Consumption, Colon Cancer Risk and All-Cause Mortality	Arvanitakis, M.	2023	10.1053/j.gastro.2022.11.046		✓		✓	
Association between dietary intake of acrylamide and increased risk of mortality in women: Evidence from the E3N prospective cohort	Marques, C.; Frenoy, P.; Elbaz, A.; Laouali, N.; Shah, S.; Severi, G.; Mancini, F.R.	2024	10.1016/j.scitotenv.2023.167514		✓			
Which components of the Mediterranean diet are associated with dementia? A UK Biobank cohort study	Dobreva, I.; Marston, L.; Mukadam, N.	2022	10.1007/s11357-022-00615-2	✓	✓	✓	✓	

Comparison between the impact of fermented and unfermented soy intake on the risk of liver cancer: the JPHC Study	Abe, S.K.; Sawada, N.; Ishihara, J.; Takachi, R.; Mori, N.; Yamaji, T.; Shimazu, T.; Goto, A.; Iwasaki, M.; Inoue, M.; Tsugane, S.	2021	10.1007/s00394-020-02335-9		✓			
Combined associations of body mass index and adherence to a Mediterranean-like diet with all-cause and cardiovascular mortality: A cohort study	Michaëlsson, K.; Baron, J.A.; Byberg, L.; Höijer, J.; Larsson, S.C.; Svennblad, B.; Melhus, H.; Wolk, A.; Lemming, E.W.	2020	10.1371/journal.pmed.1003331		✓			
A systematic comprehensive longitudinal evaluation of dietary factors associated with acute myocardial infarction and fatal coronary heart disease	Milanlouei, S.; Menichetti, G.; Li, Y.; Loscalzo, J.; Willett, W.C.; Barabási, A.-L.	2020	10.1038/s41467-020-19888-2		✓	✓	✓	
Should we all go pesco-vegetarian?	Estruch, R.; Sacanella, E.; Ros, E.	2021	10.1093/eurheartj/ehaa1088	✓	✓	✓	✓	
Can probiotics, prebiotics and synbiotics improve functional outcomes for older people: a systematic review	Coutts, L.; Ibrahim, K.; Tan, Q.Y.; Lim, S.E.R.; Cox, N.J.; Roberts, H.C.	2020	10.1007/s41999-020-00396-x	✓	✓	✓	✓	
Whole-grain intake in the mediterranean diet and a low protein to carbohydrates ratio can help to reduce mortality from cardiovascular disease, slow down the progression of aging, and to improve lifespan: A review	Capurso, C.	2021	10.3390/nu13082540	✓	✓			
Dairy consumption and incidence of breast cancer in the 'seguimiento universidad de navarra' (Sun) project	Aguilera-Buenosvinos, I.; Fernandez-Lazaro, C.I.; Romanos-Nanclares, A.; Gea, A.; Sánchez-Bayona, R.; Martín-Moreno, J.M.; Martínez-González, M.Á.; Toledo, E.	2021	10.3390/nu13020687			✓		
Post-Diagnostic Dietary and Lifestyle Factors and Prostate Cancer Recurrence, Progression, and Mortality	Langlais, C.S.; Graff, R.E.; Van Blarigan, E.L.; Palmer, N.R.; Washington, S.L.; Chan, J.M.; Kenfield, S.A.	2021	10.1007/s11912-021-01017-x	✓	✓	✓	✓	
Traditional Eastern European diet and mortality: prospective evidence from the HAPIEE study	Stefler, D.; Brett, D.; Sarkadi-Nagy, E.; Kopczynska, E.; Detchev, S.; Bati, A.; Scrob, M.; Koenker, D.; Aleksov, B.; Douarin, E.; Simonova, G.; Malyutina, S.; Kubinova, R.; Pajak, A.; Ruiz, M.; Peasey, A.; Pikhart, H.; Bobak, M.	2021	10.1007/s00394-020-02319-9		✓			
Dietary factors modulating colorectal carcinogenesis	Vernia, F.; Longo, S.; Stefanelli, G.; Viscido, A.; Latella, G.	2021	10.3390/nu13010143	✓	✓	✓		

Dietary intake of total polyphenols and the risk of all-cause and specific-cause mortality in Japanese adults: the Takayama study	Taguchi, C.; Kishimoto, Y.; Fukushima, Y.; Kondo, K.; Yamakawa, M.; Wada, K.; Nagata, C.	2020	10.1007/s00394-019-02136-9		✓			
Dietary saturated fat and heart disease: A narrative review	Heileson, J.L.	2020	10.1093/nutrit/nuz091	✓	✓			
Mediterranean diet and health status: Active ingredients and pharmacological mechanisms	Schwingshackl, L.; Morze, J.; Hoffmann, G.	2020	10.1111/bph.14778	✓	✓	✓	✓	
The association between dietary fibre deficiency and high-income lifestyle-associated diseases: Burkitt's hypothesis revisited	O'Keefe, S.J.	2019	10.1016/S2468-1253(19)30257-2	✓	✓	✓	✓	
Mediterranean adequacy index: Features and applications	Menotti, A.; Puddu, P.E.	2020	10.1016/B978-0-12-818649-7.00012-6		✓		✓	
A Clinician's Guide for Trending Cardiovascular Nutrition Controversies: Part II	Freeman, A.M.; Morris, P.B.; Aspry, K.; Gordon, N.F.; Barnard, N.D.; Esselstyn, C.B.; Ros, E.; Devries, S.; O'Keefe, J.; Miller, M.; Ornish, D.; Williams, K.A.; Batts, T.; Ostfeld, R.J.; Litwin, S.; Aggarwal, M.; Werner, A.; Allen, K.; White, B.; Kris-Etherton, P.	2018	10.1016/j.jacc.2018.05.030	✓	✓	✓	✓	
Preventing Lethal Prostate Cancer with Diet, Supplements, and Rx: Heart Healthy Continues to Be Prostate Healthy and "First Do No Harm" Part I	Moyad, M.A.	2018	10.1007/s11934-018-0846-4	✓	✓	✓	✓	
Recent Science and Clinical Application of Nutrition to Coronary Heart Disease	Houston, M.; Minich, D.; Sinatra, S.T.; Kahn, J.K.; Guarneri, M.	2018	10.1080/07315724.2017.1381053	✓	✓	✓	✓	
Full dairy ahead!	Stower, H.	2019	10.1038/s41591-018-0331-0	✓	✓	✓	✓	
Fermented food and non-communicable chronic diseases: A review	Gille, D.; Schmid, A.; Walther, B.; Vergères, G.	2018	10.3390/nu10040448	✓		✓		
Functional Food and Cardiovascular Disease Prevention and Treatment: A Review	Asgary, S.; Rastqar, A.; Keshvari, M.	2018	10.1080/07315724.2017.1410867	✓		✓		

Higher milk intake increases fracture risk: confounding or true association?	Sahni, S.; Soedamah-Muthu, S.S.; Weaver, C.M.	2017	10.1007/s00198-017-4088-y				✓	
Dairy Consumption and Cardiometabolic Diseases: Systematic Review and Updated Meta-Analyses of Prospective Cohort Studies	Soedamah-Muthu, S.S.; de Goede, J.	2018	10.1007/s13668-018-0253-y		✓	✓		
Peripheral Arterial Disease and Cardiovascular Risk	Chrysohoou, C.; Esposito, K.; Giugliano, D.; Panagiotakos, D.B.	2015	10.1177/0003319714556651	✓	✓	✓	✓	
Will it be cheese, bologna, or peanut butter?	Willett, W.C.	2017	10.1007/s10654-017-0257-8	✓	✓	✓	✓	
Health Benefits of Fiber Fermentation	Dahl, W.J.; Agro, N.C.; Eliasson, Å.M.; Mialki, K.L.; Olivera, J.D.; Rusch, C.T.; Young, C.N.	2017	10.1080/07315724.2016.1188737	✓	✓	✓	✓	
Dietary Patterns and Risk of Esophageal Cancer Mortality: The Japan Collaborative Cohort Study	Okada, E.; Nakamura, K.; Ukawa, S.; Sakata, K.; Date, C.; Iso, H.; Tamakoshi, A.	2016	10.1080/01635581.2016.1192202		✓			
Do dietary patterns in older age influence the development of cancer and cardiovascular disease: A longitudinal study of ageing	Nobbs, H.M.; Yaxley, A.; Thomas, J.; Delaney, C.; Koczwara, B.; Luszcz, M.; Miller, M.	2016	10.1016/j.clnu.2015.04.003		✓			
Is There a Dose–Response Relationship between Tea Consumption and All-Cause, CVD, and Cancer Mortality?	Yan, Y.; Sui, X.; Yao, B.; Lavie, C.J.; Blair, S.N.	2017	10.1080/07315724.2016.1261054		✓			
Does a Mediterranean-Type Diet Reduce Cancer Risk?	Schwingshackl, L.; Hoffmann, G.	2016	10.1007/s13668-015-0141-7	✓	✓	✓		
Chocolate intake reduces risk of cardiovascular disease: Evidence from 10 observational studies	Zhang, Z.; Xu, G.; Liu, X.	2013	10.1016/j.ijcard.2012.12.036			✓		
The JPHC study: Design and some findings on the typical Japanese diet	Tsugane, S.; Sawada, N.	2014	10.1093/jjco/hyu096			✓		
Can daily coffee consumption reduce liver disease-related mortality?	Ng, V.; Saab, S.	2013	10.1016/j.cgh.2013.05.042	✓		✓	✓	

Dietary Patterns in a Circumpolar Context: A Cultural Approach to the Interpretation of Three Studies on Mediterranean, Traditional Sami, and Low-Carbohydrate Dietary Pattern Scores in Northernmost Sweden	Nilsson, L.M.	2015	10.1016/B978-0-12-407849-9.00051-8		✓		✓	
Vegetable intake, but not fruit intake, is associated with a reduction in the risk of cancer incidence and mortality in middle-aged Korean Men	Choi, Y.; Lee, J.E.; Bae, J.-M.; Li, Z.-M.; Kim, D.-H.; Lee, M.-S.; Ahn, Y.-O.; Shin, M.-H.	2015	10.3945/jn.114.209437		✓			
Study of functional foods consumption patterns among decedents dying due to various causes of death	Singh, R.B.; Visen, P.; Sharma, D.; Sharma, S.; Mondol, R.; Sharma, J.P.; Sharma, M.; Tokunaga, M.; Takahashi, T.; Mishra, S.; Sharma, A.; Jain, M.; Marinho, F.; Pal, R.	2014	10.2174/1876396001508010016	✓	✓		✓	
Dairy consumption and the risk of 15-year cardiovascular disease mortality in a cohort of older Australians	Louie, J.C.Y.; Flood, V.M.; Burlutsky, G.; Rangan, A.M.; Gill, T.P.; Mitchell, P.	2013	10.3390/nu5020441		✓			
Development of the mediterranean soup for enteral nutrition and for prevention of cardiovascular diseases	Singh, R.B.; Choudhury, J.; de Meester, F.; Wilson, D.W.	2012	10.2174/1876396001205010090	✓	✓			
Higher adherence to French dietary guidelines and chronic diseases in the prospective SU.VI.MAX cohort	Kesse-Guyot, E.; Touvier, M.; Henegar, A.; Czernichow, S.; Galan, P.; Hercberg, S.; Castetbon, K.	2011	10.1038/ejcn.2011.61		✓	✓		
Importance of sleep disorders in assessing the association between coffee consumption and all-cause mortality	Card, A.J.	2013	10.1016/j.mayocp.2013.10.005	✓	✓	✓	✓	
The benefits of milk and dairy consumption: Addressing misconceptions about dairy and vascular disease	Elwood, P.C.; Pickering, J.E.; Livingstone, K.; Givens, I.	2013					✓	
The consumption of milk and dairy foods and the incidence of vascular disease and diabetes: An overview of the evidence	Elwood, P.C.; Pickering, J.E.; Ian Givens, D.; Gallacher, J.E.	2010	10.1007/s11745-010-3412-5		✓			
Another reason to drink your morning cup of coffee: Commentary	Keyhani, S.	2008					✓	

Nutrition, physical activity, and cardiovascular disease: An update	Ignarro, L.J.; Balestrieri, M.L.; Napoli, C.	2007	10.1016/j.cardiores.2006.06.030	✓	✓	✓	✓	
Dairy products as essential contributors of (micro-) nutrients in reference food patterns: An outline for elderly people	van Staveren, W.A.; Steijns, J.M.; de Groot, L.C.P.G.M.	2008	10.1080/07315724.2008.10719753	✓	✓	✓	✓	
Coffee drinking and hepatocellular carcinoma: An update	Bravi, F.; Bosetti, C.; Tavani, A.; La Vecchia, C.	2009	10.1002/hep.23272	✓		✓	✓	
A prospective study of variety of healthy foods and mortality in women	Michels, K.B.; Wolk, A.	2002	10.1093/ije/31.4.847		✓	✓		
Dietary patterns among older Europeans: The EPIC-Elderly study	Bamia, C.; Orfanos, P.; Ferrari, P.; Overvad, K.; Hundborg, H.H.; Tjønneland, A.; Olsen, A.; Kesse, E.; Boutron-Ruault, M.-C.; Clavel-Chapelon, F.; Nagel, G.; Boffetta, P.; Boeing, H.; Hoffmann, K.; Trichopoulos, D.; Baibas, N.; Psaltopoulou, T.; Norat, T.; Slimani, N.; Palli, D.; Krogh, V.; Panico, S.; Tumino, R.; Sacerdote, C.; Bueno-de-Mesquita, H.B.; Ocké, M.C.; Peeters, P.H.; van Rossum, C.T.; Quirós, J.-R.; Sánchez, M.-J.; Navarro, C.; Barricarte, A.; Dorronsoro, M.; Berglund, G.; Wirfält, E.; Hallmans, G.; Johansson, I.; Bingham, S.; Khaw, K.-T.; Spencer, E.A.; Roddam, A.W.; Riboli, E.; Trichopoulou, A.	2005	10.1079/BJN20051456		✓	✓		
Coffee consumption and risk of colorectal cancer in a population based prospective cohort of Swedish women	Terry, P.; Bergkvist, L.; Holmberg, L.; Wolk, A.	2001	10.1136/gut.49.1.87			✓		
Screening the average risk population for colorectal cancer: The Israeli experience 1985-97	Niv, Y.	2003	10.1046/j.1463-1318.2003.00467.x			✓		
Changes in food selection in northern Sweden - the MONICA surveys 1986 and 1990	Johansson, I.; Hallmans, G.	1994						Fulltext not available
Cancer patterns and lifestyle among Japanese immigrants and their descendants in the city of São Paulo, Brazil	Tsugane, S.; Hamada, G.S.; Karita, K.; Tsubono, Y.; Laurenti, R.	1996		✓	✓		✓	
A 20-year Follow-up Study of the Relation Between Life Style and Stroke in the Oki	Fukuzawa, Y.; Kishimoto, T.; Tada, M.	1992	10.2188/jea.2.35	✓	✓	✓	✓	

Islands, Shimane Prefecture, Japan								
Frequency and Effects of Food Intake, Cigarette Smoking an Alcohol Consumption Among the Residents in Hisayama, Kyushu Island, Japan II. Relationship to Total Mortality, Development of Cerebrovascular Diseases and Malignant Neoplasma During 15 year Follow-up Period	Hirota, Y.; Takeshita, S.	1986	10.3143/geriatrics.23.163					Paper in Japanese
