# Supplementary material for: Fermented foods consumption, all-cause, and cause-specific mortality: a meta-analysis of prospective cohort studies
Source: Front Nutr. 2026 Feb 26;13:1714437. doi: 10.3389/fnut.2026.1714437 (PMC12979560; doi:10.3389/fnut.2026.1714437)
Supplement: Supplementary file 4 [file Data_Sheet_4.docx]

**Supplementary Material 4** Extracted Cohort Data on Fermented Food and Mortality for Meta-analysis

Adapted and adjusted from Paveljšek et al. (1).

| **Study & subgroup details** | **Region** | **Start year** | **Cohort** | **Age range at entry** | **Sex** | **Dietary assessm.** | **Exposure levels** | **No. of subjects** | **No. of deaths** | **Years of FU** | **HR (95% CI)** | **p-value** | **NOS** | **Adjustments** | **Ref.** |
| --- | --- | --- | --- | --- | --- | --- | --- | --- | --- | --- | --- | --- | --- | --- | --- |
| **Fermented dairy** | | | | | | | | | | | | | | | |
| **Fermented dairy / All-causes** | | | | | | | | | | | | | | | |
| Soedamah-Muthu et al. 2013 | UK | 1997 | Whitehall II cohort | 35–55 | Male & female | FFQ | T3: 105 g/day (energy-adj.)  T1: 17 g/day (ref.)* | 4526 | 237 | 11.7 | 0.65 (0.47-0.9) | 0.01 | 7 | Age, ethnicity, employment grade, smoking, BMI, alcohol intake, physical activity, family history of CHD/hypertension, fruit and vegetable, bread, meat, fish, coffee and tea intake | (2) |
| van Aerde et al. 2013 | Netherlands | 1989 | The Hoorn Study | 50–75 | Male & female | FFQ | Q4: ≥14.7 g/day Q1: ≤6.0 g/day (ref.)* | 1956 | 403 | 12.4 | 0.98 (0.87-1.11) | 0.77 | 8 | Age, sex, BMI, smoking, educational level, total energy intake, alcohol consumption, physical activity, intake of meat, fish, bread, vegetables, fruit, coffee, tea | (3) |
| Virtanen et al. 2019 | Finland | 1984 | KIHD | 42–60 | Male | 4-day food record | Q4: 437 g/day (energy-adj.)  Q1: 3 g/day (ref.)* | 2641 | 1225 | 22.3 | 1.04 (0.89-1.21) | 0.53 | 8 | Age, examination year, energy intake, income, education years, marital status, leisure-time physical activity, pack-years of smoking, alcohol intake, BMI, diagnosis of type 2 diabetes, cardiovascular disease, cancer, or hypertension or use of cardiac, hypercholesterolemia, hypertension, or diabetes medications, intakes of fiber and saturated, monounsaturated, polyunsaturated, trans fatty acids | (4) |
| Guo et al. 2022 | Denmark | 1982 | MONICA | 30–60 | Male & female | 7-day weighed food record | Q4: 249.7 g/week  Q1: 12.6 g/week** | 1746 | 660 | 30 | 0.99 (0.78-1.26) | 0.7 | 8 | Sex, BMI, food energy intake, alcohol consumption, education, smoking, physical activity, family history of myocardial infarction, multivitamin use, serum total cholesterol, serum triaclyglycerols, incidence of hypertension | (5) |
| **Fermented dairy / CVD** | | | | | | | | | | | | | | | |
| van Aerde et al. 2013 | Netherlands | 1989 | The Hoorn Study | 50–75 | Male & female | FFQ | Q4: ≥14.7 g/day Q1: ≤6.0 g/day (ref.)* | 1956 | 116 | 12.4 | 1.01 (0.8-1.27) | 0.97 | 8 | Age, sex, BMI, smoking, educational level, total energy intake, alcohol consumption, physical activity, intake of meat, fish, bread, vegetables, fruit, coffee, tea | (3) |
| Praagman et al. 2015 (Stroke M.) | Netherlands | 1990 | Rotterdam Study | ≥ 55 | Male & female | SFFQ | T3: >100 g/day  T1: <50 g/day (ref.)* | 4235 | 182 | 17.3 | 0.85 (0.59-1.22) | 0.85 | 8 | Age, sex, total energy intake, BMI, smoking, education level, alcohol intake, intakes of vegetables, fruit, meat, bread, fish coffee, tea | (6) |
| Praagman et al. 2015 (CHD M.) | Netherlands | 1990 | Rotterdam Study | ≥ 55 | Male & female | SFFQ | T3: >100 g/day  T1: <50 g/day (ref.)* | 4235 | 350 | 17.3 | 0.92 (0.71-1.19) | 0.55 | 8 | Age, sex, total energy intake, BMI, smoking, education level, alcohol intake, intakes of vegetables, fruit, meat, bread, fish coffee, tea | (6) |
| Silva et al. 2022 | Brazil | 2008 | ELSA-Brasil | 35–74 | Male & female | FFQ | Males  Q4: ≥ 361.5 g/day (energy-adj.)  Q1: ≤ 102.8 g/day (ref.)  Females  Q4: ≥ 479.1 g/day (energy-adj.)  Q1: ≤ 187.3 g/day (ref.)* | 6671 | 42 | 8 | 1.34 (0.53-3.36) |  | 8 | Age, sex, educational level, physical activity, smoking status, alcohol consumption, BMI, diabetes, hypertension, hypercholesterolemia | (7) |

| **Study & subgroup details** | | **Region** | | | | **Start year** | | | **Cohort** | | **Age range at entry** | | | **Sex** | | | **Dietary assessm.** | | | **Exposure levels** | | | **No. of subjects** | | | **No. of deaths** | | | **Years of FU** | | | **HR (95% CI)** | | | **p-value** | | | **NOS** | | | **Adjustments** | | | **Ref.** |
| --- | --- | --- | --- | --- | --- | --- | --- | --- | --- | --- | --- | --- | --- | --- | --- | --- | --- | --- | --- | --- | --- | --- | --- | --- | --- | --- | --- | --- | --- | --- | --- | --- | --- | --- | --- | --- | --- | --- | --- | --- | --- | --- | --- | --- |
| **Fermented milks** | | | | | | | | | | | | | | | | | | | | | | | | | | | | | | | | | | | | | | | | | | | | |
| **Fermented milks / All-causes** | | | | | | | | | | | | | | | | | | | | | | | | | | | | | | | | | | | | | | | | | | | | |
| Bonthuis et al. 2010 | | Australia | | | | 1992 | | | Nambour Skin Cancer Study | | 25–78 | | | Male & female | | | FFQ | | | Q3: 76 g/day Q1: 0 g/day (ref.)* | | | 1529 | | | 177 | | | 14.4 | | | 1.22 (0.77-1.93) | | | 0.36 | | | 8 | | | Age, sex, total energy intake, BMI, alcohol intake, school leaving age, physical activity level, pack-years of smoking, dietary supplement use, beta-carotene treatment during trial, presence of any medical condition, dietary calcium | | | (8) |
| Goldbohm et al. 2011 (Female LF Milk) | | Netherlands | | | | 1986 | | | NLCS | | 55–69 | | | Male & female (data shown for females) | | | FFQ | | | Q3/C3: 192 g/day  Q1/C1: 0 g/day (ref.)* | | | 62573 | | | 5478 | | | 10 | | | 1.02 (0.95-1.09) | | | 0.265 | | | 8 | | | Age, education, smoking, physical activity, BMI, multivitamin use, alcohol, energy, energy-adjusted mono- and polyunsaturated fat intakes, vegetable and fruit consumption | | | (9) |
| Goldbohm et al. 2011 (Female FF Milk) | | Netherlands | | | | 1986 | | | NLCS | | 55–69 | | | Male & female (data shown for females) | | | FFQ | | | Q2/C2: 53 g/day  Q1/C1: 0 g/day (ref.)* | | | 62573 | | | 5478 | | | 10 | | | 0.93 (0.87-1.0) | | |  | | | 8 | | | Age, education, smoking, physical activity, BMI, multivitamin use, alcohol, energy, energy-adjusted mono- and polyunsaturated fat intakes, vegetable and fruit consumption | | | (9) |
| Goldbohm et al. 2011 (Male LF Milk) | | Netherlands | | | | 1986 | | | NLCS | | 55–69 | | | Male & female (data shown for males) | | | FFQ | | | Q3/C3: 146 g/day  Q1/C1: 0 g/day (ref.)* | | | 58279 | | | 10658 | | | 10 | | | 0.97 (0.93-1.03) | | | 0.893 | | | 8 | | | Age, education, smoking, physical activity, BMI, multivitamin use, alcohol, energy, energy-adjusted mono- and polyunsaturated fat intakes, vegetable and fruit consumption | | | (9) |
| Goldbohm et al. 2011 (Male FF Milk) | | Netherlands | | | | 1986 | | | NLCS | | 55–69 | | | Male & female (data shown for males) | | | FFQ | | | Q2/C2: 53 g/day  Q1/C1: 0 g/day (ref.)* | | | 58279 | | | 10658 | | | 10 | | | 0.93 (0.88-0.98) | | |  | | | 8 | | | Age, education, smoking, physical activity, BMI, multivitamin use, alcohol, energy, energy-adjusted mono- and polyunsaturated fat intakes, vegetable and fruit consumption | | | (9) |
| Sluik et al. 2014 | | Multiple | | | | 1992 | | | EPIC | | 45–64 | | | Male & female | | | FFQ | | | Q3: 71 g/day (energy-adj.)  Q1: 3 g/day (ref.)* | | | 258911 | | | 12135 | | | 9.9 | | | 1 (1.0-1.0) | | | 0.21 | | | 8 | | | Age, region, sex, educational attainment, alcohol consumption, physical activity, smoking status and smoking intensity, factor loadings for the first three dietary patterns derived from factor analysis on 26 food groups | | | (10) |
| Praagman et al. 2015 | | Netherlands | | | | 1993 | | | EPIC-NL | | 20–70 | | | Male & female | | | FFQ | | | Q4: 144.5 g/day (energy-adj.)  Q1: 3.8 g/day (ref.)* | | | 34409 | | | 2436 | | | 15 | | | 0.97 (0.86-1.09) | | | 0.9 | | | 8 | | | Age, sex, smoking habit, BMI, physical activity, education level, hypertension at baseline, intakes of alcohol and energy-adjusted intakes of fruit and vegetables, total energy intake | | | (11) |
| Bongard et al. 2016 | | France | | | | 1995 | | | MONICA | | 45–64 | | | Male | | | 3-day food record | | | Q4: 1188 g/day (energy-adj.)  Q1: 0 g/day (ref.)* | | | 960 | | | 150 | | | 14.8 | | | 0.96 (0.62-1.49) | | | 0.35 | | | 8 | | | Center, age, payment of income tax, obesity, alcohol consumption, smoking habits, physical activity, presence of a serious chronic condition, diet quality score | | | (12) |
| Tognon et al. 2017 | | Sweden | | | | 1986 | | | NSHDS | | 24–74 | | | Male & female | | | FFQ | | | Q4: ≥2.5 times/day (energy-adj.)  Q1: <1 times/week (ref.) | | | 103256 | | | 6892 | | | 13.7 | | | 0.96 (0.92-1.01) | | |  | | | 8 | | | Age, sex, BMI, screening year, smoking, education, energy intake | | | (13) |
| Farvid et al. 2017 | | Iran | | | | 2004 | | | Golestan Study | | 36–85 | | | Male & female | | | FFQ | | | Q5: 0.9 servings/day  Q1: 0.1 servings/day (ref.)** | | | 42403 | | | 3291 | | | 8 | | | 0.89 (0.89-1.0) | | | 0.03 | | | 8 | | | Age, ethnicity, education, marital status, residency, smoking, opium use, alcohol use, BMI, systolic blood pressure, occupational physical activity, family history of cancer, wealth score, medication use, energy intake | | | (14) |
| Dehghan et al. 2018 | | Multiple | | | | 2003 | | | PURE | | 35–70 | | | Male & female | | | FFQ | | | Q4: 1.5 servings/day Q1: 0 servings/day (ref.)** | | | 136384 | | | 6796 | | | 9.1 | | | 0.83 (0.69-0.99) | | | 0.0051 | | | 8 | | | Age, sex, education, urban or rural location, smoking, physical activity, history of diabetes, family history of cardiovascular disease, family history of cancer, quintiles of fruit, vegetable, red meat, starchy foods intake, total energy intake, centre was included as a random effect to account for clustering by location | | | (15) |
| Pala et al. 2019 | | Italy | | | | 1993 | | | EPIC-Italy | | 45–64 | | | Male & female | | | FFQ | | | Q4: >120 g/day  Q1 : 0 g/day (ref.) | | | 45009 | | | 2468 | | | 14.9 | | | 0.95 (0.82-1.09) | | | 0.14 | | | 8 | | | Region, sex, age, energy intake, weight, height, waist-to-hip ratio, alcohol consumption, smoking status, physical activity, relative index of inequality, Italian Mediterranean Index, intake of sugar | | | (16) |
| Mazidi et al. 2019 | | USA | | | | 1999 | | | NHANES | | > 20 | | | Male & female | | | 24-h recall | | | Q4: 3.08 cup eq servings/d Q1: 0.25 cup eq/day (ref.)* | | | 24474 | | | 3520 | | | 6.4 | | | 0.93 (0.85-1.01) | | | 0.523 | | | 9 | | | Age, sex, race, education, marital status, poverty to income ratio, total energy intake, physical activity, smoking, alcohol consumption, carbohydrates, saturated fat, protein, dietary fiber, BMI, hypertension, diabetes | | | (17) |
| Schmid et al. 2020 (Female) | | USA | | | | 1980 | | | NHS | | 30–59 | | | Male & female (data shown for females) | | | SFFQ | | | >4 servings/week  Never (ref.) | | | 82348 | | | 20831 | | | 32 | | | 0.91 (0.85-0.98) | | | 0.34 | | | 8 | | | Height, BMI, BMI at age 18 (females) or 21 (males), race, physical activity, smoking status, history of hypertension, history of hypercholesterolemia, history of diabetes, family history of cancer, family history of diabetes, family history of myocardial infarction, current multivitamin use, regular aspirin use, menopausal status and hormone use (only for females), total caloric intake, alcohol consumption, glycemic load, intakes of unprocessed red meat, processed meat, nuts, fruits, vegetables, total calcium, total fiber | | | (18) |
| Schmid et al. 2020 (Male) | | USA | | | | 1980 | | | NHS | | 40–79 | | | Male & female (data shown for males) | | | SFFQ | | | >4 servings/week  Never (ref.) | | | 40278 | | | 12397 | | | 26 | | | 1.05 (0.95-1.16) | | | 0.7 | | | 8 | | | Height, BMI, BMI at age 18 (females) or 21 (males), race, physical activity, smoking status, history of hypertension, history of hypercholesterolemia, history of diabetes, family history of cancer, family history of diabetes, family history of myocardial infarction, current multivitamin use, regular aspirin use, menopausal status and hormone use (only for females), total caloric intake, alcohol consumption, glycemic load, intakes of unprocessed red meat, processed meat, nuts, fruits, vegetables, total calcium, total fiber | | | (18) |
| Nakanishi et al. 2021 | | Japan | | | | 2009 | | | Yamagata Study | | 40–74 | | | Male & female | | | FFQ | | | High (>1 times/day) None (<1 times/month) (ref.) | | | 14264 | | | 265 | | | 9 | | | 0.7 (0.49-0.99) | | | 0.04 | | | 7 | | | Age, sex, smoking status, alcohol consumption, BMI, hypertension, diabetes, education | | | (19) |
| Sonestedt et al. 2021 | | Sweden | | | | 1991 | | | MDCS | | 45–73 | | | Male & female | | | SFFQ, food record, and interview | | | C5: > 300 g/day C1: 0 g/day (ref.) | | | 26190 | | | 7156 | | | 19 | | | 0.9 (0.79-1.03) | | | 0.009 | | | 9 | | | Age, sex, diet assessment method, season, energy, BMI, education, physical activity, smoking, alcohol habits, diet (fruit and vegetables, meat, fiber, sugar-sweetened beverages) | | | (20) |
| Lin et al. 2022 | | USA | | | | 1999 | | | NHANES | | > 18 | | | Male & female | | | 24-h recall | | | NR | | | 32625 | | | 3881 | | | 8.1 | | | 0.83 (0.71-0.98) | | | 0.035 | | | 9 | | | Age, sex, race, BMI, white blood cell count, hemoglobin, platelet count, total bilirubin, creatinine, blood urea nitrogen, hypertension, diabetes, asthma congestive heart failure, coronary heart disease, stroke, chronic bronchitis, and cancer | | | (21) |
| Lu et al. 2022 (Male) | | Japan | | | | 1990 | | | Miyagi Cohort | | 40–64 | | | Male & female (data shown for males) | | | FFQ | | | 3 times/week or almost daily  Almost never (ref.) | | | 16565 | | | 4354 | | | 25 | | | 1.04 (0.92-1.17) | | | 0.253 | | | 9 | | | Age (continuous), education level, BMI, smoking status, alcohol drinking status, history of hypertension, history of diabetes, energy intake, fish intake, vegetable and fruit intake | | | (22) |
| Lu et al. 2022 (Female) | | Japan | | | | 1990 | | | Miyagi Cohort | | 40–64 | | | Male & female (data shown for females) | | | FFQ | | | 3 times/week or almost daily  Almost never (ref.) | | | 17596 | | | 2522 | | | 25 | | | 0.92 (0.81-1.03) | | | 0.146 | | | 9 | | | Age (continuous), education level, BMI, smoking status, alcohol drinking status, history of hypertension, history of diabetes, energy intake, fish intake, vegetable and fruit intake | | | (22) |
| Guo et al. 2022 | | Denmark | | | | 1982 | | | MONICA | | 30–60 | | | Male & female | | | 7-day weighed food record | | | Q4: 194.7 g/week  Q1: 0 g/week** | | | 1746 | | | 660 | | | 30 | | | 1.05 (0.76-1.45) | | | 0.95 | | | 8 | | | Sex, BMI, food energy intake, alcohol consumption, education, smoking, physical activity, family history of myocardial infarction, multivitamin use, serum total cholesterol, serum triaclyglycerols, incidence of hypertension | | | (5) |
| Ge et al. 2023 (Male) | | Japan | | | | 1995 | | | JPHC | | 40–69 | | | Male & female (data shown for males) | | | FFQ | | | Q4: 76.6 g/day (energy-adj.)  Q1: 0 g/day (ref.)* | | | 43117 | | | 14211 | | | 19.3 | | | 0.94 (0.9-0.995) | | | 0.02 | | | 7 | | | Age, study area, smoking status, alcohol frequency, BMI, physical activity, hypertension with medication, self, reported diabetes, green tea, coffee, energy-adjusted consumption of vegetables and fruits, total energy and total fat, menopausal status (only for females), exogenous hormone use (only for females), dairy intake | | | (23) |
| Ge et al. 2023 (Female) | | Japan | | | | 1995 | | | JPHC | | 40–69 | | | Male & female (data shown for females) | | | FFQ | | | Q4: 100.8 g/day (energy-adj.)  Q1: 0 g/day (ref.)* | | | 50193 | | | 9547 | | | 19.3 | | | 0.93 (0.88-0.99) | | | 0.15 | | | 7 | | | Age, study area, smoking status, alcohol frequency, BMI, physical activity, hypertension with medication, self, reported diabetes, green tea, coffee, energy-adjusted consumption of vegetables and fruits, total energy and total fat, menopausal status (only for females), exogenous hormone use (only for females), dairy intake | | | (23) |
| Miyagawa et al. 2024 (Female) | | Japan | | | | 2005 | | | J-MICC | | 35–69 | | | Male & female (data shown for females) | | | FFQ | | | T3: 61.6 g/day (energy-adj.)  T1: 5.5 g/day (ref.)* | | | 45597 | | | 1344 | | | 12 | | | 0.87 (0.76-0.997) | | | 0.046 | | | 9 | | | Age, study site, history of cardiometabolic diseases, BMI, smoking status, drinking status, physical activity, dietary intake of red meat, fish, vegetables, fruits | | | (24) |
| Miyagawa et al. 2024 (Male) | | Japan | | | | 2005 | | | J-MICC | | 35–69 | | | Male & female (data shown for males) | | | FFQ | | | T3: 40.2 g/day (energy-adj.)  T1: 0 g/day (ref.)* | | | 34118 | | | 2379 | | | 12 | | | 0.9 (0.82-0.999) | | | 0.034 | | | 9 | | | Age, study site, history of cardiometabolic diseases, BMI, smoking status, drinking status, physical activity, dietary intake of red meat, fish, vegetables, fruits | | | (24) |
| **Fermented milks / CVD** | | | | | | | | | | | | | | | | | | | | | | | | | | | | | | | | | | | | | | | | | | | | |
| Bonthuis et al. 2010 | | Australia | | | | 1992 | | | Nambour Skin Cancer Study | | 25–78 | | | Male & female | | | FFQ | | | Q3: 76 g/day Q1: 0 g/day (ref.)* | | | 1529 | | | 61 | | | 14.4 | | | 0.65 (0.26-1.58) | | | 0.52 | | | 8 | | | Age, sex, total energy intake, BMI, alcohol intake, school leaving age, physical activity level, pack-years of smoking, dietary supplement use, beta-carotene treatment during trial, presence of any medical condition, dietary calcium | | | (8) |
| Goldbohm et al. 2011 (Male IHD M., FF Milk) | | Netherlands | | | | 1986 | | | NLCS | | 55–69 | | | Male & female (data shown for males) | | | FFQ | | | Q2/C2: 53 g/day  Q1/C1: 0 g/day (ref.)* | | | 58279 | | | 1997 | | | 10 | | | 0.77 (0.64-0.92) | | |  | | | 8 | | | Age, education, smoking, physical activity, BMI, multivitamin use, alcohol, energy, energy-adjusted mono- and polyunsaturated fat intakes, vegetable and fruit consumption | | | (9) |
| Goldbohm et al. 2011 (Female Stroke M., LF Milk) | | Netherlands | | | | 1986 | | | NLCS | | 55–69 | | | Male & female (data shown for females) | | | FFQ | | | Q3/C3: 192 g/day  Q1/C1: 0 g/day (ref.)* | | | 62573 | | | 322 | | | 10 | | | 0.76 (0.55-1.05) | | | 0.032 | | | 8 | | | Age, education, smoking, physical activity, BMI, multivitamin use, alcohol, energy, energy-adjusted mono- and polyunsaturated fat intakes, vegetable and fruit consumption | | | (9) |
| Goldbohm et al. 2011 (Female Stroke M., FF Milk) | | Netherlands | | | | 1986 | | | NLCS | | 55–69 | | | Male & female (data shown for females) | | | FFQ | | | Q2/C2: 53 g/day  Q1/C1: 0 g/day (ref.)* | | | 62573 | | | 322 | | | 10 | | | 0.81 (0.6-1.1) | | |  | | | 8 | | | Age, education, smoking, physical activity, BMI, multivitamin use, alcohol, energy, energy-adjusted mono- and polyunsaturated fat intakes, vegetable and fruit consumption | | | (9) |
| Goldbohm et al. 2011 (Male Stroke M., LF Milk) | | Netherlands | | | | 1986 | | | NLCS | | 55–69 | | | Male & female (data shown for males) | | | FFQ | | | Q3/C3: 146 g/day  Q1/C1: 0 g/day (ref.)* | | | 58279 | | | 520 | | | 10 | | | 0.84 (0.64-1.11) | | | 0.382 | | | 8 | | | Age, education, smoking, physical activity, BMI, multivitamin use, alcohol, energy, energy-adjusted mono- and polyunsaturated fat intakes, vegetable and fruit consumption | | | (9) |
| Goldbohm et al. 2011 (Male Stroke M., FF Milk) | | Netherlands | | | | 1986 | | | NLCS | | 55–69 | | | Male & female (data shown for males) | | | FFQ | | | Q2/C2: 53 g/day  Q1/C1: 0 g/day (ref.)* | | | 58279 | | | 520 | | | 10 | | | 0.86 (0.65-1.15) | | |  | | | 8 | | | Age, education, smoking, physical activity, BMI, multivitamin use, alcohol, energy, energy-adjusted mono- and polyunsaturated fat intakes, vegetable and fruit consumption | | | (9) |
| Goldbohm et al. 2011 (Female IHD M., LF Milk) | | Netherlands | | | | 1986 | | | NLCS | | 55–69 | | | Male & female (data shown for females) | | | FFQ | | | Q3/C3: 192 g/day  Q1/C1: 0 g/day (ref.)* | | | 62573 | | | 692 | | | 10 | | | 1.19 (0.94-1.51) | | | 0.056 | | | 8 | | | Age, education, smoking, physical activity, BMI, multivitamin use, alcohol, energy, energy-adjusted mono- and polyunsaturated fat intakes, vegetable and fruit consumption | | | (9) |
| Goldbohm et al. 2011 (Female IHD M., FF Milk) | | Netherlands | | | | 1986 | | | NLCS | | 55–69 | | | Male & female (data shown for females) | | | FFQ | | | Q2/C2: 53 g/day  Q1/C1: 0 g/day (ref.)* | | | 62573 | | | 692 | | | 10 | | | 0.99 (0.79-1.24) | | |  | | | 8 | | | Age, education, smoking, physical activity, BMI, multivitamin use, alcohol, energy, energy-adjusted mono- and polyunsaturated fat intakes, vegetable and fruit consumption | | | (9) |
| Goldbohm et al. 2011 (Male IHD M., LF Milk) | | Netherlands | | | | 1986 | | | NLCS | | 55–69 | | | Male & female (data shown for males) | | | FFQ | | | Q3/C3: 146 g/day  Q1/C1: 0 g/day (ref.)* | | | 58279 | | | 1997 | | | 10 | | | 0.93 (0.78-1.11) | | | 0.961 | | | 8 | | | Age, education, smoking, physical activity, BMI, multivitamin use, alcohol, energy, energy-adjusted mono- and polyunsaturated fat intakes, vegetable and fruit consumption | | | (9) |
| Praagman et al. 2015 | | Netherlands | | | | 1993 | | | EPIC-NL | | 20–70 | | | Male & female | | | FFQ | | | Q4: 144.5 g/day (energy-adj.)  Q1: 3.8 g/day (ref.)* | | | 34409 | | | 727 | | | 15 | | | 0.98 (0.79-1.22) | | | 0.9 | | | 8 | | | Age, sex, smoking habit, BMI, physical activity, education level, hypertension at baseline, intakes of alcohol and energy-adjusted intakes of fruit and vegetables, total energy intake | | | (11) |
| Praagman et al. 2015 (Stroke M.) | | Netherlands | | | | 1990 | | | Rotterdam Study | | ≥ 55 | | | Male & female | | | SFFQ | | | T3: >100 g/day  T1: <50 g/day (ref.)* | | | 4235 | | | 182 | | | 17.3 | | | 1.01 (0.71-1.44) | | | 0.93 | | | 8 | | | Age, sex, total energy intake, BMI, smoking, education level, alcohol intake, intakes of vegetables, fruit, meat, bread, fish coffee, tea | | | (6) |
| Praagman et al. 2015 (CHD M.) | | Netherlands | | | | 1990 | | | Rotterdam Study | | ≥ 55 | | | Male & female | | | SFFQ | | | T3: >100 g/day  T1: <50 g/day (ref.)* | | | 4235 | | | 350 | | | 17.3 | | | 0.98 (0.76-1.26) | | | 0.84 | | | 8 | | | Age, sex, total energy intake, BMI, smoking, education level, alcohol intake, intakes of vegetables, fruit, meat, bread, fish coffee, tea | | | (6) |
| Farvid et al. 2017 | | Iran | | | | 2004 | | | Golestan Study | | 36–85 | | | Male & female | | | FFQ | | | Q5: 0.9 servings/day  Q1: 0.1 servings/day (ref.)** | | | 42403 | | | 1467 | | | 8 | | | 0.84 (0.7-1.0) | | | 0.03 | | | 8 | | | Age, ethnicity, education, marital status, residency, smoking, opium use, alcohol use, BMI, systolic blood pressure, occupational physical activity, family history of cancer, wealth score, medication use, energy intake | | | (14) |
| Pala et al. 2019 | | Italy | | | | 1993 | | | EPIC-Italy | | 45–64 | | | Male & female | | | FFQ | | | Q4: >120 g/day  Q1 : 0 g/day (ref.) | | | 45009 | | | 249 | | | 14.9 | | | 0.85 (0.59-1.23) | | | 0.15 | | | 8 | | | Region, sex, age, energy intake, weight, height, waist-to-hip ratio, alcohol consumption, smoking status, physical activity, relative index of inequality, Italian Mediterranean Index, intake of sugar | | | (16) |
| Mazidi et al. 2019 | | USA | | | | 1999 | | | NHANES | | > 20 | | | Male & female | | | 24-h recall | | | Q4: 3.08 cup eq servings/d Q1: 0.25 cup eq/day (ref.)* | | | 24474 | | | 709 | | | 6.4 | | | 0.98 (0.97-0.99) | | | 0.125 | | | 9 | | | Age, sex, race, education, marital status, poverty to income ratio, total energy intake, physical activity, smoking, alcohol consumption, carbohydrates, saturated fat, protein, dietary fiber, BMI, hypertension, diabetes | | | (17) |
| Schmid et al. 2020 (Female) | | USA | | | | 1980 | | | NHS | | 30–59 | | | Male & female (data shown for females) | | | SFFQ | | | >4 servings/week  Never (ref.) | | | 82348 | | | 4207 | | | 31.5 | | | 0.92 (0.79-1.08) | | | 0.41 | | | 8 | | | Height, BMI, BMI at age 18 (females) or 21 (males), race, physical activity, smoking status, history of hypertension, history of hypercholesterolemia, history of diabetes, family history of cancer, family history of diabetes, family history of myocardial infarction, current multivitamin use, regular aspirin use, menopausal status and hormone use (only for females), total caloric intake, alcohol consumption, glycemic load, intakes of unprocessed red meat, processed meat, nuts, fruits, vegetables, total calcium, total fiber | | | (18) |
| Schmid et al. 2020 (Male) | | USA | | | | 1980 | | | NHS | | 40–79 | | | Male & female (data shown for males) | | | SFFQ | | | >4 servings/week  Never (ref.) | | | 40278 | | | 3733 | | | 25 | | | 1.1 (0.93-1.3) | | | 0.42 | | | 8 | | | Height, BMI, BMI at age 18 (females) or 21 (males), race, physical activity, smoking status, history of hypertension, history of hypercholesterolemia, history of diabetes, family history of cancer, family history of diabetes, family history of myocardial infarction, current multivitamin use, regular aspirin use, menopausal status and hormone use (only for females), total caloric intake, alcohol consumption, glycemic load, intakes of unprocessed red meat, processed meat, nuts, fruits, vegetables, total calcium, total fiber | | | (18) |
| Nakanishi et al. 2021 | | Japan | | | | 2009 | | | Yamagata Study | | 40–74 | | | Male & female | | | FFQ | | | High (>1 times/day) None (<1 times/month) (ref.) | | | 14264 | | | 40 | | | 9 | | | 1.06 (0.39-2.84) | | | 0.91 | | | 7 | | | Age, sex, smoking status, alcohol consumption, BMI, hypertension, diabetes, education | | | (19) |
| Lin et al. 2022 | | USA | | | | 1999 | | | NHANES | | > 18 | | | Male & female | | | 24-h recall | | | NR | | | 32625 | | | 651 | | | 8.1 | | | 0.68 (0.43-1.08) | | | 0.109 | | | 9 | | | Age, sex, race, BMI, white blood cell count, hemoglobin, platelet count, total bilirubin, creatinine, blood urea nitrogen, hypertension, diabetes, asthma congestive heart failure, coronary heart disease, stroke, chronic bronchitis, and cancer | | | (21) |
| Lu et al. 2022 (Male) | | Japan | | | | 1990 | | | Miyagi Cohort | | 40–64 | | | Male & female (data shown for males) | | | FFQ | | | 3 times/week or almost daily  Almost never (ref.) | | | 16565 | | | 1048 | | | 25 | | | 0.99 (0.78-1.26) | | | 0.488 | | | 9 | | | Age (continuous), education level, BMI, smoking status, alcohol drinking status, history of hypertension, history of diabetes, energy intake, fish intake, vegetable and fruit intake | | | (22) |
| Lu et al. 2022 (Female) | | Japan | | | | 1990 | | | Miyagi Cohort | | 40–64 | | | Male & female (data shown for females) | | | FFQ | | | 3 times/week or almost daily  Almost never (ref.) | | | 17596 | | | 645 | | | 25 | | | 0.87 (0.69-1.11) | | | 0.221 | | | 9 | | | Age (continuous), education level, BMI, smoking status, alcohol drinking status, history of hypertension, history of diabetes, energy intake, fish intake, vegetable and fruit intake | | | (22) |
| Zhang et al. 2023 | | Swedish | | | | 1991 | | | MDCS | | 41–73 | | | Male & female | | | FFQ | | | Per 100 g/day increase | | | 20499 | | | 2531 | | | 21 | | | 0.95 (0.92-0.99) | | | 0.02 | | | 9 | | | Age, sex, dietary assessment version, season, total energy intake, leisure-time physical activity, smoking status, alcohol consumption, educational level, heredity score (including cancer, myocardial infarction, stroke, diabetes), diet quality index | | | (25) |
| Ge et al. 2023 (Female) | | Japan | | | | 1995 | | | JPHC | | 40–69 | | | Male & female (data shown for females) | | | FFQ | | | Q4: 100.8 g/day (energy-adj.)  Q1: 0 g/day (ref.)* | | | 50193 | | | 2582 | | | 19.3 | | | 0.91 (0.81-1.02) | | | 0.32 | | | 7 | | | Age, study area, smoking status, alcohol frequency, BMI, physical activity, hypertension with medication, self, reported diabetes, green tea, coffee, energy-adjusted consumption of vegetables and fruits, total energy and total fat, menopausal status (only for females), exogenous hormone use (only for females), dairy intake | | | (23) |
| Ge et al. 2023 (Male) | | Japan | | | | 1995 | | | JPHC | | 40–69 | | | Male & female (data shown for males) | | | FFQ | | | Q4: 76.6 g/day (energy-adj.)  Q1: 0 g/day (ref.)* | | | 43117 | | | 3379 | | | 19.3 | | | 0.9 (0.81-0.996) | | | 0.02 | | | 7 | | | Age, study area, smoking status, alcohol frequency, BMI, physical activity, hypertension with medication, self, reported diabetes, green tea, coffee, energy-adjusted consumption of vegetables and fruits, total energy and total fat, menopausal status (only for females), exogenous hormone use (only for females), dairy intake | | | (23) |
| Miyagawa et al. 2024 (Male) | | Japan | | | | 2005 | | | J-MICC | | 35–69 | | | Male & female (data shown for males) | | | FFQ | | | T3: 40.2 g/day (energy-adj.)  T1: 0 g/day (ref.)* | | | 34118 | | | 307 | | | 12 | | | 0.96 (0.73-1.28) | | | 0.772 | | | 9 | | | Age, study site, history of hypertension, diabetes, dyslipidemia, BMI, smoking status, drinking status, physical activity, dietary intake of red meat, fish, vegetables, fruits | | | (24) |
| Miyagawa et al. 2024 (Female) | | Japan | | | | 2005 | | | J-MICC | | 35–69 | | | Male & female (data shown for females) | | | FFQ | | | T3: 61.6 g/day (energy-adj.)  T1: 5.5 g/day (ref.)* | | | 45597 | | | 223 | | | 12 | | | 0.64 (0.46-0.9) | | | 0.007 | | | 9 | | | Age, study site, history of hypertension, diabetes, dyslipidemia, BMI, smoking status, drinking status, physical activity, dietary intake of red meat, fish, vegetables, fruits | | | (24) |
| **Fermented milks / Cancer** | | | | | | | | | | | | | | | | | | | | | | | | | | | | | | | | | | | | | | | | | | | | |
| Khan et al. 2004 (Female) | | Japan | | | | 1984 | | | Hokkaido Study | | 40–97 | | | Male & female (data shown for females) | | | FFQ | | | C5: several times per week, everyday  C1: never, several times per year, several times per month (ref.) | | | 1634 | | | 89 | | | 14.8 | | | 0.7 (0.4-1.3) | | |  | | | 7 | | | Age, health status, health education, health screening, smoking | | | (26) |
| Khan et al. 2004 (Male) | | Japan | | | | 1984 | | | Hokkaido Study | | 40–97 | | | Male & female (data shown for males) | | | FFQ | | | C5: several times per week, everyday  C1: never, several times per year, several times per month (ref.) | | | 1524 | | | 155 | | | 13.8 | | | 0.8 (0.5-1.3) | | |  | | | 7 | | | Age, health status, health education, health screening, smoking | | | (26) |
| Matsumoto et al. 2007 | | Japan | | | | 1992 | | | JMS | | 19–93 | | | Male & female | | | FFQ | | | Q5: almost everyday Q1: seldom (ref.) | | | 11606 | | | 255 | | | 9.15 | | | 1.48 (0.59-3.72) | | | 0.41 | | | 5 | | | Age, sex | | | (27) |
| Bonthuis et al. 2010 | | Australia | | | | 1992 | | | Nambour Skin Cancer Study | | 25–78 | | | Male & female | | | FFQ | | | Q3: 76 g/day Q1: 0 g/day (ref.) | | | 1529 | | | 58 | | | 14.4 | | | There was no association between yoghurt intake and Cancer mortality (Data are not provided). (nan-nan) | | | | | | 8 | | | Age, sex, total energy intake, BMI, alcohol intake, school leaving age, physical activity level, pack-years of smoking, dietary supplement use, beta-carotene treatment during trial, presence of any medical condition, dietary calcium | | | (8) |
| Praagman et al. 2015 | | Netherlands | | | | 1993 | | | EPIC-NL | | 20–70 | | | Male & female | | | FFQ | | | Q4: 144.5 g/day (energy-adj.)  Q1: 3.8 g/day (ref.)* | | | 34409 | | | 1216 | | | 15 | | | 1.02 (0.86-1.2) | | | 0.6 | | | 8 | | | Age, sex, smoking habit, BMI, physical activity, education level, hypertension at baseline, intakes of alcohol and energy-adjusted intakes of fruit and vegetables, total energy intake | | | (11) |
| Farvid et al. 2017 | | Iran | | | | 2004 | | | Golestan Study | | 36–85 | | | Male & female | | | FFQ | | | Q5: 0.9 servings/day  Q1: 0.1 servings/day (ref.)** | | | 42403 | | | 859 | | | 8 | | | 0.86 (0.69-1.08) | | | 0.18 | | | 8 | | | Age, ethnicity, education, marital status, residency, smoking, opium use, alcohol use, BMI, systolic blood pressure, occupational physical activity, family history of cancer, wealth score, medication use, energy intake | | | (14) |
| Mazidi et al. 2019 | | USA | | | | 1999 | | | NHANES | | > 20 | | | Male & female | | | 24-h recall | | | Q4: 3.08 cup eq servings/d Q1: 0.25 cup eq/day (ref.)* | | | 24474 | | | 827 | | | 6.4 | | | 1 (0.99-1.01) | | | 0.352 | | | 9 | | | Age, sex, race, education, marital status, poverty to income ratio, total energy intake, physical activity, smoking, alcohol consumption, carbohydrates, saturated fat, protein, dietary fiber, BMI, hypertension, diabetes | | | (17) |
| Pala et al. 2019 | | Italy | | | | 1993 | | | EPIC-Italy | | 45–64 | | | Male & female | | | FFQ | | | Q4: >120 g/day  Q1 : 0 g/day (ref.) | | | 45009 | | | 1456 | | | 14.9 | | | 1 (0.83-1.2) | | | 0.82 | | | 8 | | | Region, sex, age, energy intake, weight, height, waist-to-hip ratio, alcohol consumption, smoking status, physical activity, relative index of inequality, Italian Mediterranean Index, intake of sugar | | | (16) |
| Schmid et al. 2020 (Female) | | USA | | | | 1980 | | | NHS | | 30–59 | | | Male & female (data shown for females) | | | SFFQ | | | >4 servings/week  Never (ref.) | | | 82348 | | | 7985 | | | 31.5 | | | 0.87 (0.78-0.98) | | | 0.04 | | | 8 | | | Height, BMI, BMI at age 18 (females) or 21 (males), race, physical activity, smoking status, history of hypertension, history of hypercholesterolemia, history of diabetes, family history of cancer, family history of diabetes, family history of myocardial infarction, current multivitamin use, regular aspirin use, menopausal status and hormone use (only for females), total caloric intake, alcohol consumption, glycemic load, intakes of unprocessed red meat, processed meat, nuts, fruits, vegetables, total calcium, total fiber | | | (18) |
| Schmid et al. 2020 (Male) | | USA | | | | 1980 | | | NHS | | 40–79 | | | Male & female (data shown for males) | | | SFFQ | | | >4 servings/week  Never (ref.) | | | 40278 | | | 4000 | | | 25 | | | 0.95 (0.79-1.13) | | | 0.19 | | | 8 | | | Height, BMI, BMI at age 18 (females) or 21 (males), race, physical activity, smoking status, history of hypertension, history of hypercholesterolemia, history of diabetes, family history of cancer, family history of diabetes, family history of myocardial infarction, current multivitamin use, regular aspirin use, menopausal status and hormone use (only for females), total caloric intake, alcohol consumption, glycemic load, intakes of unprocessed red meat, processed meat, nuts, fruits, vegetables, total calcium, total fiber | | | (18) |
| Nakanishi et al. 2021 | | Japan | | | | 2009 | | | Yamagata Study | | 40–74 | | | Male & female | | | FFQ | | | High (>1 times/day) None (<1 times/month) (ref.) | | | 14264 | | | 90 | | | 9 | | | 0.53 (0.27-0.99) | | | 0.047 | | | 7 | | | Age, sex, smoking status, alcohol consumption, BMI, hypertension, diabetes, education | | | (19) |
| Lin et al. 2022 | | USA | | | | 1999 | | | NHANES | | > 18 | | | Male & female | | | 24-h recall | | | NR | | | 32625 | | | 863 | | | 8.1 | | | 1 (0.72-1.38) | | | 0.972 | | | 9 | | | Age, sex, race, BMI, white blood cell count, hemoglobin, platelet count, total bilirubin, creatinine, blood urea nitrogen, hypertension, diabetes, asthma congestive heart failure, coronary heart disease, stroke, chronic bronchitis, and cancer | | | (21) |
| Lu et al. 2022 (Male) | | Japan | | | | 1990 | | | Miyagi Cohort | | 40–64 | | | Male & female (data shown for males) | | | FFQ | | | 3 times/week or almost daily  Almost never (ref.) | | | 16565 | | | 1713 | | | 25 | | | 1.03 (0.85-1.24) | | | 0.791 | | | 9 | | | Age (continuous), education level, BMI, smoking status, alcohol drinking status, history of hypertension, history of diabetes, energy intake, fish intake, vegetable and fruit intake | | | (22) |
| Lu et al. 2022 (Female) | | Japan | | | | 1990 | | | Miyagi Cohort | | 40–64 | | | Male & female (data shown for females) | | | FFQ | | | 3 times/week or almost daily  Almost never (ref.) | | | 17596 | | | 839 | | | 25 | | | 1.1 (0.89-1.34) | | | 0.541 | | | 9 | | | Age (continuous), education level, BMI, smoking status, alcohol drinking status, history of hypertension, history of diabetes, energy intake, fish intake, vegetable and fruit intake | | | (22) |
| Ge et al. 2023 (Male) | | Japan | | | | 1995 | | | JPHC | | 40–69 | | | Male & female (data shown for males) | | | FFQ | | | Q4: 76.6 g/day (energy-adj.)  Q1: 0 g/day (ref.)* | | | 43117 | | | 5364 | | | 19.3 | | | 0.91 (0.84-0.99) | | | 0.04 | | | 7 | | | Age, study area, smoking status, alcohol frequency, BMI, physical activity, hypertension with medication, self, reported diabetes, green tea, coffee, energy-adjusted consumption of vegetables and fruits, total energy and total fat, menopausal status (only for females), exogenous hormone use (only for females), dairy intake | | | (23) |
| Ge et al. 2023 (Female) | | Japan | | | | 1995 | | | JPHC | | 40–69 | | | Male & female (data shown for females) | | | FFQ | | | Q4: 100.8 g/day (energy-adj.)  Q1: 0 g/day (ref.)* | | | 50193 | | | 3076 | | | 19.3 | | | 0.92 (0.82-1.02) | | | 0.33 | | | 7 | | | Age, study area, smoking status, alcohol frequency, BMI, physical activity, hypertension with medication, self, reported diabetes, green tea, coffee, energy-adjusted consumption of vegetables and fruits, total energy and total fat, menopausal status (only for females), exogenous hormone use (only for females), dairy intake | | | (23) |
| Miyagawa et al. 2024 (Female) | | Japan | | | | 2005 | | | J-MICC | | 35–69 | | | Male & female (data shown for females) | | | FFQ | | | T3: 61.6 g/day (energy-adj.)  T1: 5.5 g/day (ref.)* | | | 45597 | | | 725 | | | 12 | | | 1.03 (0.85-1.24) | | | 0.793 | | | 9 | | | Age, study site, history of hypertension, diabetes, dyslipidemia, BMI, smoking status, drinking status, physical activity, dietary intake of red meat, fish, vegetables, fruits | | | (24) |
| Miyagawa et al. 2024 (Male) | | Japan | | | | 2005 | | | J-MICC | | 35–69 | | | Male & female (data shown for males) | | | FFQ | | | T3: 40.2 g/day (energy-adj.)  T1: 0 g/day (ref.)* | | | 34118 | | | 1363 | | | 12 | | | 0.93 (0.81-1.06) | | | 0.251 | | | 9 | | | Age, study site, history of hypertension, diabetes, dyslipidemia, BMI, smoking status, drinking status, physical activity, dietary intake of red meat, fish, vegetables, fruits | | | (24) |
| **Yogurt** | | | | | | | | | | | | | | | | | | | | | | | | | | | | | | | | | | | | | | | | | | | | |
| **Yogurt / All-causes** | | | | | | | | | | | | | | | | | | | | | | | | | | | | | | | | | | | | | | | | | | | | |
| Bonthuis et al. 2010 | | Australia | | | | 1992 | | | Nambour Skin Cancer Study | | 25–78 | | | Male & female | | | FFQ | | | Q3: 76 g/day Q1: 0 g/day (ref.)* | | | 1529 | | | 177 | | | 14.4 | | | 1.22 (0.77-1.93) | | | 0.36 | | | 8 | | | Age, sex, total energy intake, BMI, alcohol intake, school leaving age, physical activity level, pack-years of smoking, dietary supplement use, beta-carotene treatment during trial, presence of any medical condition, dietary calcium | | | (8) |
| Sluik et al. 2014 | | Multiple | | | | 1992 | | | EPIC | | 45–64 | | | Male & female | | | FFQ | | | Q3: 71 g/day (energy-adj.)  Q1: 3 g/day (ref.)* | | | 258911 | | | 12135 | | | 9.9 | | | 1 (1.0-1.0) | | | 0.21 | | | 8 | | | Age, region, sex, educational attainment, alcohol consumption, physical activity, smoking status and smoking intensity, factor loadings for the first three dietary patterns derived from factor analysis on 26 food groups | | | (10) |
| Praagman et al. 2015 | | Netherlands | | | | 1993 | | | EPIC-NL | | 20–70 | | | Male & female | | | FFQ | | | Q4: 144.5 g/day (energy-adj.)  Q1: 3.8 g/day (ref.)* | | | 34409 | | | 2436 | | | 15 | | | 0.97 (0.86-1.09) | | | 0.9 | | | 8 | | | Age, sex, smoking habit, BMI, physical activity, education level, hypertension at baseline, intakes of alcohol and energy-adjusted intakes of fruit and vegetables, total energy intake | | | (11) |
| Farvid et al. 2017 | | Iran | | | | 2004 | | | Golestan Study | | 36–85 | | | Male & female | | | FFQ | | | Q5: 0.9 servings/day  Q1: 0.1 servings/day (ref.)** | | | 42403 | | | 3291 | | | 8 | | | 0.89 (0.89-1.0) | | | 0.03 | | | 8 | | | Age, ethnicity, education, marital status, residency, smoking, opium use, alcohol use, BMI, systolic blood pressure, occupational physical activity, family history of cancer, wealth score, medication use, energy intake | | | (14) |
| Dehghan et al. 2018 | | Multiple | | | | 2003 | | | PURE | | 35–70 | | | Male & female | | | FFQ | | | Q4: 1.5 servings/day Q1: 0 servings/day (ref.)* | | | 136384 | | | 6796 | | | 9.1 | | | 0.83 (0.69-0.99) | | | 0.0051 | | | 8 | | | Age, sex, education, urban or rural location, smoking, physical activity, history of diabetes, family history of cardiovascular disease, family history of cancer, quintiles of fruit, vegetable, red meat, starchy foods intake, total energy intake, centre was included as a random effect to account for clustering by location | | | (15) |
| Pala et al. 2019 | | Italy | | | | 1993 | | | EPIC-Italy | | 45–64 | | | Male & female | | | FFQ | | | Q4: >120 g/day  Q1 : 0 g/day (ref.) | | | 45009 | | | 2468 | | | 14.9 | | | 0.95 (0.82-1.09) | | | 0.14 | | | 8 | | | Region, sex, age, energy intake, weight, height, waist-to-hip ratio, alcohol consumption, smoking status, physical activity, relative index of inequality, Italian Mediterranean Index, intake of sugar | | | (16) |
| Mazidi et al. 2019 | | USA | | | | 1999 | | | NHANES | | > 20 | | | Male & female | | | 24-h recall | | | Q4: 3.08 cup eq servings/d Q1: 0.25 cup eq/day (ref.)* | | | 24474 | | | 3520 | | | 6.4 | | | 0.93 (0.85-1.01) | | | 0.523 | | | 9 | | | Age, sex, race, education, marital status, poverty to income ratio, total energy intake, physical activity, smoking, alcohol consumption, carbohydrates, saturated fat, protein, dietary fiber, BMI, hypertension, diabetes | | | (17) |
| Schmid et al. 2020 (Male) | | USA | | | | 1980 | | | NHS | | 40–79 | | | Male & female (data shown for males) | | | SFFQ | | | >4 servings/week  Never (ref.) | | | 40278 | | | 12397 | | | 26 | | | 1.05 (0.95-1.16) | | | 0.7 | | | 8 | | | Height, BMI, BMI at age 18 (females) or 21 (males), race, physical activity, smoking status, history of hypertension, history of hypercholesterolemia, history of diabetes, family history of cancer, family history of diabetes, family history of myocardial infarction, current multivitamin use, regular aspirin use, menopausal status and hormone use (only for females), total caloric intake, alcohol consumption, glycemic load, intakes of unprocessed red meat, processed meat, nuts, fruits, vegetables, total calcium, total fiber | | | (18) |
| Schmid et al. 2020 (Female) | | USA | | | | 1980 | | | NHS | | 30–59 | | | Male & female (data shown for females) | | | SFFQ | | | >4 servings/week  Never (ref.) | | | 82348 | | | 20831 | | | 32 | | | 0.91 (0.85-0.98) | | | 0.34 | | | 8 | | | Height, BMI, BMI at age 18 (females) or 21 (males), race, physical activity, smoking status, history of hypertension, history of hypercholesterolemia, history of diabetes, family history of cancer, family history of diabetes, family history of myocardial infarction, current multivitamin use, regular aspirin use, menopausal status and hormone use (only for females), total caloric intake, alcohol consumption, glycemic load, intakes of unprocessed red meat, processed meat, nuts, fruits, vegetables, total calcium, total fiber | | | (18) |
| Nakanishi et al. 2021 | | Japan | | | | 2009 | | | Yamagata Study | | 40–74 | | | Male & female | | | FFQ | | | High (>1 times/day) None (<1 times/month) (ref.) | | | 14264 | | | 265 | | | 9 | | | 0.7 (0.49-0.99) | | | 0.04 | | | 7 | | | Age, sex, smoking status, alcohol consumption, BMI, hypertension, diabetes, education | | | (19) |
| Guo et al. 2022 | | Denmark | | | | 1982 | | | MONICA | | 30–60 | | | Male & female | | | 7-day weighed food record | | | Q4: 194.7 g/week  Q1: 0 g/week** | | | 1746 | | | 660 | | | 30 | | | 1.05 (0.76-1.45) | | | 0.95 | | | 8 | | | Sex, BMI, food energy intake, alcohol consumption, education, smoking, physical activity, family history of myocardial infarction, multivitamin use, serum total cholesterol, serum triaclyglycerols, incidence of hypertension | | | (5) |
| Lin et al. 2022 | | USA | | | | 1999 | | | NHANES | | > 18 | | | Male & female | | | 24-h recall | | | NR | | | 32625 | | | 3881 | | | 8.1 | | | 0.83 (0.71-0.98) | | | 0.035 | | | 9 | | | Age, sex, race, BMI, white blood cell count, hemoglobin, platelet count, total bilirubin, creatinine, blood urea nitrogen, hypertension, diabetes, asthma congestive heart failure, coronary heart disease, stroke, chronic bronchitis, and cancer | | | (21) |
| Lu et al. 2022 (Male) | | Japan | | | | 1990 | | | Miyagi Cohort | | 40–64 | | | Male & female (data shown for males) | | | FFQ | | | 3 times/week or almost daily  Almost never (ref.) | | | 16565 | | | 4354 | | | 25 | | | 1.04 (0.92-1.17) | | | 0.253 | | | 9 | | | Age (continuous), education level, BMI, smoking status, alcohol drinking status, history of hypertension, history of diabetes, energy intake, fish intake, vegetable and fruit intake | | | (22) |
| Lu et al. 2022 (Female) | | Japan | | | | 1990 | | | Miyagi Cohort | | 40–64 | | | Male & female (data shown for females) | | | FFQ | | | 3 times/week or almost daily  Almost never (ref.) | | | 17596 | | | 2522 | | | 25 | | | 0.92 (0.81-1.03) | | | 0.146 | | | 9 | | | Age (continuous), education level, BMI, smoking status, alcohol drinking status, history of hypertension, history of diabetes, energy intake, fish intake, vegetable and fruit intake | | | (22) |
| Miyagawa et al. 2024 (Male) | | Japan | | | | 2005 | | | J-MICC | | 35–69 | | | Male & female (data shown for males) | | | FFQ | | | T3: 40.2 g/day (energy-adj.)  T1: 0 g/day (ref.)* | | | 34118 | | | 2379 | | | 12 | | | 0.9 (0.82-0.999) | | | 0.034 | | | 9 | | | Age, study site, history of cardiometabolic diseases, BMI, smoking status, drinking status, physical activity, dietary intake of red meat, fish, vegetables, fruits | | | (24) |
| Miyagawa et al. 2024 (Female) | | Japan | | | | 2005 | | | J-MICC | | 35–69 | | | Male & female (data shown for females) | | | FFQ | | | T3: 61.6 g/day (energy-adj.)  T1: 5.5 g/day (ref.)* | | | 45597 | | | 1344 | | | 12 | | | 0.87 (0.76-0.997) | | | 0.046 | | | 9 | | | Age, study site, history of cardiometabolic diseases, BMI, smoking status, drinking status, physical activity, dietary intake of red meat, fish, vegetables, fruits | | | (24) |
| **Study & subgroup details** | | **Region** | | | | **Start year** | | | **Cohort** | | **Age range at entry** | | | **Sex** | | | **Dietary assessm.** | | | **Exposure levels** | | | **No. of subjects** | | | **No. of deaths** | | | **Years of FU** | | | **HR (95% CI)** | | | **p-value** | | | **NOS** | | | **Adjustments** | | | **Ref.** |
| **Yogurt / CVD** | | | | | | | | | | | | | | | | | | | | | | | | | | | | | | | | | | | | | | | | | | | | |
| Bonthuis et al. 2010 | | Australia | | | | 1992 | | | Nambour Skin Cancer Study | | 25–78 | | | Male & female | | | FFQ | | | Q3: 76 g/day Q1: 0 g/day (ref.)* | | | 1529 | | | 61 | | | 14.4 | | | 0.65 (0.26-1.58) | | | 0.52 | | | 8 | | | Age, sex, total energy intake, BMI, alcohol intake, school leaving age, physical activity level, pack-years of smoking, dietary supplement use, beta-carotene treatment during trial, presence of any medical condition, dietary calcium | | | (8) |
| Praagman et al. 2015 | | Netherlands | | | | 1993 | | | EPIC-NL | | 20–70 | | | Male & female | | | FFQ | | | Q4: 144.5 g/day (energy-adj.)  Q1: 3.8 g/day (ref.)* | | | 34409 | | | 727 | | | 15 | | | 0.98 (0.79-1.22) | | | 0.9 | | | 8 | | | Age, sex, smoking habit, BMI, physical activity, education level, hypertension at baseline, intakes of alcohol and energy-adjusted intakes of fruit and vegetables, total energy intake | | | (11) |
| Praagman et al. 2015 (Stroke M.) | | Netherlands | | | | 1990 | | | Rotterdam Study | | ≥ 55 | | | Male & female | | | SFFQ | | | T3: >100 g/day  T1: <50 g/day (ref.)* | | | 4235 | | | 182 | | | 17.3 | | | 1.01 (0.71-1.44) | | | 0.93 | | | 8 | | | Age, sex, total energy intake, BMI, smoking, education level, alcohol intake, intakes of vegetables, fruit, meat, bread, fish coffee, tea | | | (6) |
| Praagman et al. 2015 (CHD M.) | | Netherlands | | | | 1990 | | | Rotterdam Study | | ≥ 55 | | | Male & female | | | SFFQ | | | T3: >100 g/day  T1: <50 g/day (ref.)* | | | 4235 | | | 350 | | | 17.3 | | | 0.98 (0.76-1.26) | | | 0.84 | | | 8 | | | Age, sex, total energy intake, BMI, smoking, education level, alcohol intake, intakes of vegetables, fruit, meat, bread, fish coffee, tea | | | (6) |
| Farvid et al. 2017 | | Iran | | | | 2004 | | | Golestan Study | | 36–85 | | | Male & female | | | FFQ | | | Q5: 0.9 servings/day  Q1: 0.1 servings/day (ref.)** | | | 42403 | | | 1467 | | | 8 | | | 0.84 (0.7-1.0) | | | 0.03 | | | 8 | | | Age, ethnicity, education, marital status, residency, smoking, opium use, alcohol use, BMI, systolic blood pressure, occupational physical activity, family history of cancer, wealth score, medication use, energy intake | | | (14) |
| Pala et al. 2019 | | Italy | | | | 1993 | | | EPIC-Italy | | 45–64 | | | Male & female | | | FFQ | | | Q4: >120 g/day  Q1 : 0 g/day (ref.) | | | 45009 | | | 249 | | | 14.9 | | | 0.85 (0.59-1.23) | | | 0.15 | | | 8 | | | Region, sex, age, energy intake, weight, height, waist-to-hip ratio, alcohol consumption, smoking status, physical activity, relative index of inequality, Italian Mediterranean Index, intake of sugar | | | (16) |
| Mazidi et al. 2019 | | USA | | | | 1999 | | | NHANES | | > 20 | | | Male & female | | | 24-h recall | | | Q4: 3.08 cup eq servings/d Q1: 0.25 cup eq/day (ref.)* | | | 24474 | | | 709 | | | 6.4 | | | 0.98 (0.97-0.99) | | | 0.125 | | | 9 | | | Age, sex, race, education, marital status, poverty to income ratio, total energy intake, physical activity, smoking, alcohol consumption, carbohydrates, saturated fat, protein, dietary fiber, BMI, hypertension, diabetes | | | (17) |
| Schmid et al. 2020 (Male) | | USA | | | | 1980 | | | NHS | | 40–79 | | | Male & female (data shown for males) | | | SFFQ | | | >4 servings/week  Never (ref.) | | | 40278 | | | 3733 | | | 25 | | | 1.1 (0.93-1.3) | | | 0.42 | | | 8 | | | Height, BMI, BMI at age 18 (females) or 21 (males), race, physical activity, smoking status, history of hypertension, history of hypercholesterolemia, history of diabetes, family history of cancer, family history of diabetes, family history of myocardial infarction, current multivitamin use, regular aspirin use, menopausal status and hormone use (only for females), total caloric intake, alcohol consumption, glycemic load, intakes of unprocessed red meat, processed meat, nuts, fruits, vegetables, total calcium, total fiber | | | (18) |
| Schmid et al. 2020 (Female) | | USA | | | | 1980 | | | NHS | | 30–59 | | | Male & female (data shown for females) | | | SFFQ | | | >4 servings/week  Never (ref.) | | | 82348 | | | 4207 | | | 31.5 | | | 0.92 (0.79-1.08) | | | 0.41 | | | 8 | | | Height, BMI, BMI at age 18 (females) or 21 (males), race, physical activity, smoking status, history of hypertension, history of hypercholesterolemia, history of diabetes, family history of cancer, family history of diabetes, family history of myocardial infarction, current multivitamin use, regular aspirin use, menopausal status and hormone use (only for females), total caloric intake, alcohol consumption, glycemic load, intakes of unprocessed red meat, processed meat, nuts, fruits, vegetables, total calcium, total fiber | | | (18) |
| Nakanishi et al. 2021 | | Japan | | | | 2009 | | | Yamagata Study | | 40–74 | | | Male & female | | | FFQ | | | High (>1 times/day) None (<1 times/month) (ref.) | | | 14264 | | | 40 | | | 9 | | | 1.06 (0.39-2.84) | | | 0.91 | | | 7 | | | Age, sex, smoking status, alcohol consumption, BMI, hypertension, diabetes, education | | | (19) |
| Lin et al. 2022 | | USA | | | | 1999 | | | NHANES | | > 18 | | | Male & female | | | 24-h recall | | | NR | | | 32625 | | | 651 | | | 8.1 | | | 0.68 (0.43-1.08) | | | 0.109 | | | 9 | | | Age, sex, race, BMI, white blood cell count, hemoglobin, platelet count, total bilirubin, creatinine, blood urea nitrogen, hypertension, diabetes, asthma congestive heart failure, coronary heart disease, stroke, chronic bronchitis, and cancer | | | (21) |
| Lu et al. 2022 (Male) | | Japan | | | | 1990 | | | Miyagi Cohort | | 40–64 | | | Male & female (data shown for males) | | | FFQ | | | 3 times/week or almost daily  Almost never (ref.) | | | 16565 | | | 1048 | | | 25 | | | 0.99 (0.78-1.26) | | | 0.488 | | | 9 | | | Age (continuous), education level, BMI, smoking status, alcohol drinking status, history of hypertension, history of diabetes, energy intake, fish intake, vegetable and fruit intake | | | (22) |
| Lu et al. 2022 (Female) | | Japan | | | | 1990 | | | Miyagi Cohort | | 40–64 | | | Male & female (data shown for females) | | | FFQ | | | 3 times/week or almost daily  Almost never (ref.) | | | 17596 | | | 645 | | | 25 | | | 0.87 (0.69-1.11) | | | 0.221 | | | 9 | | | Age (continuous), education level, BMI, smoking status, alcohol drinking status, history of hypertension, history of diabetes, energy intake, fish intake, vegetable and fruit intake | | | (22) |
| Miyagawa et al. 2024 (Male) | | Japan | | | | 2005 | | | J-MICC | | 35–69 | | | Male & female (data shown for males) | | | FFQ | | | T3: 40.2 g/day (energy-adj.)  T1: 0 g/day (ref.)* | | | 34118 | | | 307 | | | 12 | | | 0.96 (0.73-1.28) | | | 0.772 | | | 9 | | | Age, study site, history of hypertension, diabetes, dyslipidemia, BMI, smoking status, drinking status, physical activity, dietary intake of red meat, fish, vegetables, fruits | | | (24) |
| Miyagawa et al. 2024 (Female) | | Japan | | | | 2005 | | | J-MICC | | 35–69 | | | Male & female (data shown for females) | | | FFQ | | | T3: 61.6 g/day (energy-adj.)  T1: 5.5 g/day (ref.)* | | | 45597 | | | 223 | | | 12 | | | 0.64 (0.46-0.9) | | | 0.007 | | | 9 | | | Age, study site, history of hypertension, diabetes, dyslipidemia, BMI, smoking status, drinking status, physical activity, dietary intake of red meat, fish, vegetables, fruits | | | (24) |
| **Yogurt / Cancer** | | | | | | | | | | | | | | | | | | | | | | | | | | | | | | | | | | | | | | | | | | | | |
| Khan et al. 2004 (Male) | | Japan | | | | 1984 | | | Hokkaido Study | | 40–97 | | | Male & female (data shown for males) | | | FFQ | | | C5: several times per week, everyday  C1: never, several times per year, several times per month (ref.) | | | 1524 | | | 155 | | | 13.8 | | | 0.8 (0.5-1.3) | | |  | | | 7 | | | Age, health status, health education, health screening, smoking | | | (26) |
| Khan et al. 2004 (Female) | | Japan | | | | 1984 | | | Hokkaido Study | | 40–97 | | | Male & female (data shown for females) | | | FFQ | | | C5: several times per week, everyday  C1: never, several times per year, several times per month (ref.) | | | 1634 | | | 89 | | | 14.8 | | | 0.7 (0.4-1.3) | | |  | | | 7 | | | Age, health status, health education, health screening, smoking | | | (26) |
| Matsumoto et al. 2007 | | Japan | | | | 1992 | | | JMS | | 19–93 | | | Male & female | | | FFQ | | | Q5: almost everyday Q1: seldom (ref.) | | | 11606 | | | 255 | | | 9.15 | | | 1.48 (0.59-3.72) | | | 0.41 | | | 5 | | | Age, sex | | | (27) |
| Bonthuis et al. 2010 | | Australia | | | | 1992 | | | Nambour Skin Cancer Study | | 25–78 | | | Male & female | | | FFQ | | | Q3: 76 g/day Q1: 0 g/day (ref.) | | | 1529 | | | 58 | | | 14.4 | | | There was no association between yoghurt intake and Cancer mortality (Data are not provided). (nan-nan) | | | | | | 8 | | | Age, sex, total energy intake, BMI, alcohol intake, school leaving age, physical activity level, pack-years of smoking, dietary supplement use, beta-carotene treatment during trial, presence of any medical condition, dietary calcium | | | (8) |
| Praagman et al. 2015 | | Netherlands | | | | 1993 | | | EPIC-NL | | 20–70 | | | Male & female | | | FFQ | | | Q4: 144.5 g/day (energy-adj.)  Q1: 3.8 g/day (ref.)* | | | 34409 | | | 1216 | | | 15 | | | 1.02 (0.86-1.2) | | | 0.6 | | | 8 | | | Age, sex, smoking habit, BMI, physical activity, education level, hypertension at baseline, intakes of alcohol and energy-adjusted intakes of fruit and vegetables, total energy intake | | | (11) |
| Farvid et al. 2017 | | Iran | | | | 2004 | | | Golestan Study | | 36–85 | | | Male & female | | | FFQ | | | Q5: 0.9 servings/day  Q1: 0.1 servings/day (ref.)** | | | 42403 | | | 859 | | | 8 | | | 0.86 (0.69-1.08) | | | 0.18 | | | 8 | | | Age, ethnicity, education, marital status, residency, smoking, opium use, alcohol use, BMI, systolic blood pressure, occupational physical activity, family history of cancer, wealth score, medication use, energy intake | | | (14) |
| Pala et al. 2019 | | Italy | | | | 1993 | | | EPIC-Italy | | 45–64 | | | Male & female | | | FFQ | | | Q4: >120 g/day  Q1 : 0 g/day (ref.) | | | 45009 | | | 1456 | | | 14.9 | | | 1 (0.83-1.2) | | | 0.82 | | | 8 | | | Region, sex, age, energy intake, weight, height, waist-to-hip ratio, alcohol consumption, smoking status, physical activity, relative index of inequality, Italian Mediterranean Index, intake of sugar | | | (16) |
| Mazidi et al. 2019 | | USA | | | | 1999 | | | NHANES | | > 20 | | | Male & female | | | 24-h recall | | | Q4: 3.08 cup eq servings/d Q1: 0.25 cup eq/day (ref.)* | | | 24474 | | | 827 | | | 6.4 | | | 1 (0.99-1.01) | | | 0.352 | | | 9 | | | Age, sex, race, education, marital status, poverty to income ratio, total energy intake, physical activity, smoking, alcohol consumption, carbohydrates, saturated fat, protein, dietary fiber, BMI, hypertension, diabetes | | | (17) |
| Schmid et al. 2020 (Male) | | USA | | | | 1980 | | | NHS | | 40–79 | | | Male & female (data shown for males) | | | SFFQ | | | >4 servings/week  Never (ref.) | | | 40278 | | | 4000 | | | 25 | | | 0.95 (0.79-1.13) | | | 0.19 | | | 8 | | | Height, BMI, BMI at age 18 (females) or 21 (males), race, physical activity, smoking status, history of hypertension, history of hypercholesterolemia, history of diabetes, family history of cancer, family history of diabetes, family history of myocardial infarction, current multivitamin use, regular aspirin use, menopausal status and hormone use (only for females), total caloric intake, alcohol consumption, glycemic load, intakes of unprocessed red meat, processed meat, nuts, fruits, vegetables, total calcium, total fiber | | | (18) |
| Schmid et al. 2020 (Female) | | USA | | | | 1980 | | | NHS | | 30–59 | | | Male & female (data shown for females) | | | SFFQ | | | >4 servings/week  Never (ref.) | | | 82348 | | | 7985 | | | 31.5 | | | 0.87 (0.78-0.98) | | | 0.04 | | | 8 | | | Height, BMI, BMI at age 18 (females) or 21 (males), race, physical activity, smoking status, history of hypertension, history of hypercholesterolemia, history of diabetes, family history of cancer, family history of diabetes, family history of myocardial infarction, current multivitamin use, regular aspirin use, menopausal status and hormone use (only for females), total caloric intake, alcohol consumption, glycemic load, intakes of unprocessed red meat, processed meat, nuts, fruits, vegetables, total calcium, total fiber | | | (18) |
| Nakanishi et al. 2021 | | Japan | | | | 2009 | | | Yamagata Study | | 40–74 | | | Male & female | | | FFQ | | | High (>1 times/day) None (<1 times/month) (ref.) | | | 14264 | | | 90 | | | 9 | | | 0.53 (0.27-0.99) | | | 0.047 | | | 7 | | | Age, sex, smoking status, alcohol consumption, BMI, hypertension, diabetes, education | | | (19) |
| Lin et al. 2022 | | USA | | | | 1999 | | | NHANES | | > 18 | | | Male & female | | | 24-h recall | | | NR | | | 32625 | | | 863 | | | 8.1 | | | 1 (0.72-1.38) | | | 0.972 | | | 9 | | | Age, sex, race, BMI, white blood cell count, hemoglobin, platelet count, total bilirubin, creatinine, blood urea nitrogen, hypertension, diabetes, asthma congestive heart failure, coronary heart disease, stroke, chronic bronchitis, and cancer | | | (21) |
| Lu et al. 2022 (Male) | | Japan | | | | 1990 | | | Miyagi Cohort | | 40–64 | | | Male & female (data shown for males) | | | FFQ | | | 3 times/week or almost daily  Almost never (ref.) | | | 16565 | | | 1713 | | | 25 | | | 1.03 (0.85-1.24) | | | 0.791 | | | 9 | | | Age (continuous), education level, BMI, smoking status, alcohol drinking status, history of hypertension, history of diabetes, energy intake, fish intake, vegetable and fruit intake | | | (22) |
| Lu et al. 2022 (Female) | | Japan | | | | 1990 | | | Miyagi Cohort | | 40–64 | | | Male & female (data shown for females) | | | FFQ | | | 3 times/week or almost daily  Almost never (ref.) | | | 17596 | | | 839 | | | 25 | | | 1.1 (0.89-1.34) | | | 0.541 | | | 9 | | | Age (continuous), education level, BMI, smoking status, alcohol drinking status, history of hypertension, history of diabetes, energy intake, fish intake, vegetable and fruit intake | | | (22) |
| Miyagawa et al. 2024 (Male) | | Japan | | | | 2005 | | | J-MICC | | 35–69 | | | Male & female (data shown for males) | | | FFQ | | | T3: 40.2 g/day (energy-adj.)  T1: 0 g/day (ref.)* | | | 34118 | | | 1363 | | | 12 | | | 0.93 (0.81-1.06) | | | 0.251 | | | 9 | | | Age, study site, history of hypertension, diabetes, dyslipidemia, BMI, smoking status, drinking status, physical activity, dietary intake of red meat, fish, vegetables, fruits | | | (24) |
| Miyagawa et al. 2024 (Female) | | Japan | | | | 2005 | | | J-MICC | | 35–69 | | | Male & female (data shown for females) | | | FFQ | | | T3: 61.6 g/day (energy-adj.)  T1: 5.5 g/day (ref.)* | | | 45597 | | | 725 | | | 12 | | | 1.03 (0.85-1.24) | | | 0.793 | | | 9 | | | Age, study site, history of hypertension, diabetes, dyslipidemia, BMI, smoking status, drinking status, physical activity, dietary intake of red meat, fish, vegetables, fruits | | | (24) |
| **Yogurt / GI cancer** | | | | | | | | | | | | | | | | | | | | | | | | | | | | | | | | | | | | | | | | | | | | |
| Kojima et al. 2004 (Male Colon CA) | | Japan | | | | 1988 | | | JACC | | 40–79 | | | Male & female (data shown for males) | | | FFQ | | | 1–7 times/week  Seldom (ref.) | | | 45181 | | | 138 | | | 9.9 | | | 0.8 (0.42-1.51) | | | 0.37 | | | 8 | | | Age, family history of colorectal cancer, BMI, frequency of alcohol intake, current smoking status, walking time per day, educational level | | | (28) |
| Kojima et al. 2004 (Female Colon CA) | | Japan | | | | 1988 | | | JACC | | 40–79 | | | Male & female (data shown for females) | | | FFQ | | | 1–7 times/week  Seldom (ref.) | | | 62643 | | | 146 | | | 9.9 | | | 0.97 (0.61-1.56) | | | 0.93 | | | 8 | | | Age, family history of colorectal cancer, BMI, frequency of alcohol intake, current smoking status, walking time per day, educational level | | | (28) |
| Kojima et al. 2004 (Male Rectal CA) | | Japan | | | | 1988 | | | JACC | | 40–79 | | | Male & female (data shown for males) | | | FFQ | | | 1–7 times/week  Seldom (ref.) | | | 45181 | | | 116 | | | 9.9 | | | 0.46 (0.21-1.02) | | | 0.04 | | | 8 | | | Age, family history of colorectal cancer, BMI, frequency of alcohol intake, current smoking status, walking time per day, educational level | | | (28) |
| Kojima et al. 2004 (Female Rectal CA) | | Japan | | | | 1988 | | | JACC | | 40–79 | | | Male & female (data shown for females) | | | FFQ | | | 1–7 times/week  Seldom (ref.) | | | 62643 | | | 57 | | | 9.9 | | | 1.51 (0.6-3.8) | | | 0.14 | | | 8 | | | Age, family history of colorectal cancer, BMI, frequency of alcohol intake, current smoking status, walking time per day, educational level | | | (28) |
| Khan et al. 2004 (Male Stomach CA) | | Japan | | | | 1984 | | | Hokkaido Study | | 40–97 | | | Male & female (data shown for males) | | | FFQ | | | C5: several times per week, everyday  C1: never, several times per year, several times per month (ref.) | | | 1524 | | | 36 | | | 13.8 | | | 1.6 (0.8-3.6) | | |  | | | 7 | | | Age, health status, health education, health screening, smoking | | | (26) |
| Khan et al. 2004 (Female Stomach CA) | | Japan | | | | 1984 | | | Hokkaido Study | | 40–97 | | | Male & female (data shown for females) | | | FFQ | | | C5: several times per week, everyday  C1: never, several times per year, several times per month (ref.) | | | 1634 | | | 15 | | | 14.8 | | | 0.3 (0.0-2.3) | | |  | | | 7 | | | Age, health status, health education, health screening, smoking | | | (26) |
| Khan et al. 2004 (Male Pancreatic CA) | | Japan | | | | 1984 | | | Hokkaido Study | | 40–97 | | | Male & female (data shown for males) | | | FFQ | | | C5: several times per week, everyday  C1: never, several times per year, several times per month (ref.) | | | 1524 | | | 12 | | | 13.8 | | | 0.5 (0.1-4.2) | | |  | | | 7 | | | Age, health status, health education, health screening, smoking | | | (26) |
| Khan et al. 2004 (Female Pancreatic CA) | | Japan | | | | 1984 | | | Hokkaido Study | | 40–97 | | | Male & female (data shown for females) | | | FFQ | | | C5: several times per week, everyday  C1: never, several times per year, several times per month (ref.) | | | 1634 | | | 13 | | | 14.8 | | | 0.9 (0.2-3.9) | | |  | | | 7 | | | Age, health status, health education, health screening, smoking | | | (26) |
| Khan et al. 2004 (Male Colorectal CA) | | Japan | | | | 1984 | | | Hokkaido Study | | 40–97 | | | Male & female (data shown for males) | | | FFQ | | | C5: several times per week, everyday  C1: never, several times per year, several times per month (ref.) | | | 1524 | | | 15 | | | 13.8 | | | 0.7 (0.3-2.0) | | |  | | | 7 | | | Age, health status, health education, health screening, smoking | | | (26) |
| Khan et al. 2004 (Female Colorectal CA) | | Japan | | | | 1984 | | | Hokkaido Study | | 40–97 | | | Male & female (data shown for females) | | | FFQ | | | C5: several times per week, everyday  C1: never, several times per year, several times per month (ref.) | | | 1634 | | | 14 | | | 14.8 | | | 0.4 (0.1-2.8) | | |  | | | 7 | | | Age, health status, health education, health screening, smoking | | | (26) |
| Matsumoto et al. 2007 (Colon CA) | | Japan | | | | 1992 | | | JMS | | 19–93 | | | Male & female | | | FFQ | | | Q5: almost everyday Q1: seldom (ref.) | | | 11606 | | | 25 | | | 9.15 | | | 1.28 (0.3-5.48) | | | 0.74 | | | 5 | | | Age, sex | | | (27) |
| Matsumoto et al. 2007 (Stomach CA) | | Japan | | | | 1992 | | | JMS | | 19–93 | | | Male & female | | | FFQ | | | Q5: almost everyday Q1: seldom (ref.) | | | 11606 | | | 32 | | | 9.15 | | | 0.47 (0.06-3.46) | | | 0.46 | | | 5 | | | Age, sex | | | (27) |
| Matsumoto et al. 2007 (Bile duct CA) | | Japan | | | | 1992 | | | JMS | | 19–93 | | | Male & female | | | FFQ | | | Q5: almost everyday Q1: seldom (ref.) | | | 11606 | | | 13 | | | 9.15 | | | 1.17 (0.15-9.1) | | | 0.88 | | | 5 | | | Age, sex | | | (27) |
| Matsumoto et al. 2007 (Pancreatic CA) | | Japan | | | | 1992 | | | JMS | | 19–93 | | | Male & female | | | FFQ | | | Q5: almost everyday Q1: seldom (ref.) | | | 11606 | | | 10 | | | 9.15 | | | 2.77 (0.58-13.3) | | | 0.2 | | | 5 | | | Age, sex | | | (27) |
| Tokui et al. 2022 (Male Stomach CA) | | Japan | | | | 1988 | | | JACC | | 40–79 | | | Male & female (data shown for males) | | | FFQ | | | >1 times/day  None (ref.) | | | 46465 | | | 574 | | | 9.9 | | | 0.82 (0.5-1.37) | | | 0.47 | | | 6 | | | Age | | | (29) |
| Tokui et al. 2022 (Female Stomach CA) | | Japan | | | | 1988 | | | JACC | | 40–79 | | | Male & female (data shown for females) | | | FFQ | | | >1 times/day  None (ref.) | | | 64327 | | | 285 | | | 9.9 | | | 0.88 (0.47-1.64) | | | 0.93 | | | 6 | | | Age | | | (29) |
| **Yogurt / Lung cancer** | | | | | | | | | | | | | | | | | | | | | | | | | | | | | | | | | | | | | | | | | | | | |
| Ozasa et al. 2001 (Male) | | Japan | | | | 1988 | | | JACC | | 40–79 | | | Male & female (data shown for males) | | | FFQ | | | Q3: >3 dishes/day Q1: <1 dishes/day (ref.) | | | 42940 | | | 446 | | | 7.7 | | | 0.81 (0.54-1.22) | | | 0.14 | | | 8 | | | Age, parents' history of lung cancer, smoking status, smoking index, time since quitting smoking | | | (30) |
| Ozasa et al. 2001 (Female) | | Japan | | | | 1988 | | | JACC | | 40–79 | | | Male & female (data shown for females) | | | FFQ | | | Q3: >3 dishes/day Q1: <1 dishes/day (ref.) | | | 55308 | | | 126 | | | 7.7 | | | 0.82 (0.45-1.52) | | | 0.45 | | | 8 | | | Age, parents' history of lung cancer, smoking status, smoking index, time since quitting smoking | | | (30) |
| Khan et al. 2004 (Male) | | Japan | | | | 1984 | | | Hokkaido Study | | 40–97 | | | Male & female (data shown for males) | | | FFQ | | | C5: several times per week, everyday  C1: never, several times per year, several times per month (ref.) | | | 1524 | | | 41 | | | 13.8 | | | 1.3 (0.6-2.8) | | |  | | | 7 | | | Age, health status, health education, health screening, smoking | | | (26) |
| Khan et al. 2004 (Female) | | Japan | | | | 1984 | | | Hokkaido Study | | 40–97 | | | Male & female (data shown for females) | | | FFQ | | | C5: several times per week, everyday  C1: never, several times per year, several times per month (ref.) | | | 1634 | | | 10 | | | 14.8 | | | 0.5 (0.1-4.0) | | |  | | | 7 | | | Age, health status, health education, health screening, smoking | | | (26) |
| Matsumoto et al. 2007 | | Japan | | | | 1992 | | | JMS | | 19–93 | | | Male & female | | | FFQ | | | Q5: almost everyday Q1: seldom (ref.) | | | 11606 | | | 56 | | | 9.15 | | | 0.95 (0.29-3.03) | | | 0.92 | | | 5 | | | Age, sex | | | (27) |
| **Yogurt / Reproductive cancer** | | | | | | | | | | | | | | | | | | | | | | | | | | | | | | | | | | | | | | | | | | | | |
| Park et al. 2007 | | USA | | | | 1995 | | | NIH-AARP | | 50–71 | | | Male | | | FFQ | | | C5: > 3 servings/day  C1: < 0.5 servings/day (ref.) | | | 293888 | | | 178 | | | 6 | | | 0.78 (0.25-2.5) | | | 0.68 | | | 7 | | | Age, race/ethnicity, education, marital status, BMI, physical activity, smoking, alcohol consumption, history of diabetes, family history of prostate cancer, PSA screening, dietary calcium, energy intake | | | (31) |
| Sakauchi et al. 2007 | | Japan | | | | 1988 | | | JACC | | 40–79 | | | Female | | | FFQ | | | Q3: ≥1–2 times/week Q1: seldom (ref.) | | | 63541 | | | 77 | | | 13.3 | | | 1.66 (0.71-3.91) | | | 0.24 | | | 7 | | | Age, menopausal status, number of pregnancies, history of sex hormone use, BMI, physical activity, education | | | (32) |
| **Cheese** | | | | | | | | | | | | | | | | | | | | | | | | | | | | | | | | | | | | | | | | | | | | |
| **Cheese / All-causes** | | | | | | | | | | | | | | | | | | | | | | | | | | | | | | | | | | | | | | | | | | | | |
| Mann et al. 1997 | | UK | | | | 1980 | | | UK Health-Conscious Diet Cohort | | 16–79 | | | Male & female | | | SFFQ | | | C3: ≥ 5 times/week C1: < 1 times/week (ref.) | | | 10802 | | | 383 | | | 13.3 | | | 1.02 (0.76-1.37) | | |  | | | 6 | | | Age, sex, smoking, social class | | | (33) |
| Fortes et al. 2000 | | Italy | | | | 1993 | | | Rome Elderly Cohort | | ≥ 65 | | | Male & female | | | FFQ | | | T3: > 3 times/week  T1: <1 times/week (ref.) | | | 161 | | | 53 | | | 5 | | | 1.3 (0.51-3.34) | | |  | | | 7 | | | No adjustments | | | (34) |
| Bonthuis et al. 2010 | | Australia | | | | 1992 | | | Nambour Skin Cancer Study | | 25–78 | | | Male & female | | | FFQ | | | Q3: 30 g/day  Q1: 4 g/day (ref.)* | | | 1529 | | | 177 | | | 14.4 | | | 0.91 (0.57-1.45) | | | 0.56 | | | 8 | | | Age, sex, total energy intake, BMI, alcohol intake, school leaving age, physical activity level, pack-years of smoking, dietary supplement use, beta-carotene treatment during trial, presence of any medical condition, dietary calcium | | | (8) |
| Goldbohm et al. 2011 (Female) | | Netherlands | | | | 1986 | | | NLCS | | 55–69 | | | Male & female (data shown for females) | | | FFQ | | | Q5/C5: 56 g/day  Q1/C1: 1 g/day (ref.)* | | | 62573 | | | 5478 | | | 10 | | | 0.98 (0.88-1.1) | | | 0.607 | | | 8 | | | Age, education, smoking, physical activity, BMI, multivitamin use, alcohol, energy, energy-adjusted mono- and polyunsaturated fat intakes, vegetable and fruit consumption | | | (9) |
| Goldbohm et al. 2011 (Male) | | Netherlands | | | | 1986 | | | NLCS | | 55–69 | | | Male & female (data shown for males) | | | FFQ | | | Q5/C5: 56 g/day  Q1/C1: 1 g/day (ref.)* | | | 58279 | | | 10658 | | | 10 | | | 1.04 (0.96-1.12) | | | 0.083 | | | 8 | | | Age, education, smoking, physical activity, BMI, multivitamin use, alcohol, energy, energy-adjusted mono- and polyunsaturated fat intakes, vegetable and fruit consumption | | | (9) |
| van et al. Aerde 2013 | | Netherlands | | | | 1989 | | | The Hoorn Study | | 50–75 | | | Male & female | | | FFQ | | | Q4: ≥14.7 g/day Q1: ≤6.0 g/day (ref.)* | | | 1956 | | | 403 | | | 12.4 | | | 0.96 (0.84-1.09) | | | 0.51 | | | 8 | | | Age, sex, BMI, smoking, educational level, total energy intake, alcohol consumption, physical activity, intake of meat, fish, bread, vegetables, fruit, coffee, tea | | | (3) |
| Sluik et al. 2014 | | Multiple | | | | 1992 | | | EPIC | | 45–64 | | | Male & female | | | FFQ | | | Q3: 39 g/day (energy-adj.)  Q1: 24 g/day (ref.)* | | | 258911 | | | 12135 | | | 9.9 | | | 0.96 (0.95-0.98) | | | 0.23 | | | 8 | | | Age, region, sex, educational attainment, alcohol consumption, physical activity, smoking status and smoking intensity, factor loadings for the first three dietary patterns derived from factor analysis on 26 food groups | | | (10) |
| Praagman et al. 2015 | | Netherlands | | | | 1993 | | | EPIC-NL | | 20–70 | | | Male & female | | | FFQ | | | Q4: 53.2 g/day (energy-adj.)  Q1: 6.6 g/day (ref.)* | | | 34409 | | | 2436 | | | 15 | | | 1 (0.89-1.12) | | | 0.9 | | | 8 | | | Age, sex, smoking habit, BMI, physical activity, education level, hypertension at baseline, intakes of alcohol and energy-adjusted intakes of fruit and vegetables, total energy intake | | | (11) |
| Bongard et al. 2016 | | France | | | | 1995 | | | MONICA | | 45–64 | | | Male | | | 3-day food record | | | Q4: 283 g/day (energy-adj.)  Q1: 0 g/day (ref.)* | | | 960 | | | 150 | | | 14.8 | | | 1.16 (0.77-1.77) | | | 0.68 | | | 8 | | | Center, age, payment of income tax, obesity, alcohol consumption, smoking habits, physical activity, presence of a serious chronic condition, diet quality score | | | (12) |
| Farvid et al. 2017 | | Iran | | | | 2004 | | | Golestan Study | | 36–85 | | | Male & female | | | FFQ | | | Q5: 0.8 servings/day  Q1: 0 servings/day (ref.)** | | | 42403 | | | 3291 | | | 8 | | | 0.84 (0.73-0.96) | | | 0.02 | | | 8 | | | Age, ethnicity, education, marital status, residency, smoking, opium use, alcohol use, BMI, systolic blood pressure, occupational physical activity, family history of cancer, wealth score, medication use, energy intake | | | (14) |
| Tognon et al. 2017 | | Sweden | | | | 1986 | | | NSHDS | | 24–74 | | | Male & female | | | FFQ | | | Q4: ≥2.5 times/day (energy-adj.)  Q1: <1 times/week (ref.) | | | 103256 | | | 6892 | | | 13.7 | | | 0.94 (0.91-0.97) | | | 0.001 | | | 8 | | | Age, sex, BMI, screening year, smoking, education, energy intake | | | (13) |
| Dehghan et al. 2018 | | Multiple | | | | 2003 | | | PURE | | 35–70 | | | Male & female | | | FFQ | | | Q4: 1.7 servings/day Q1: 0 servings/day (ref.)* | | | 136384 | | | 6796 | | | 9.1 | | | 0.87 (0.72-1.05) | | | 0.2383 | | | 8 | | | Age, sex, education, urban or rural location, smoking, physical activity, history of diabetes, family history of cardiovascular disease, family history of cancer, quintiles of fruit, vegetable, red meat, starchy foods intake, total energy intake, centre was included as a random effect to account for clustering by location | | | (15) |
| Tognon et al. 2018 | | Sweden | | | | 1971 | | | H70 | | 70 | | | Male & female | | | Interview questionnaire | | | T3: >45 g/day (males and females)  T1: <28.7 (males) and <21.4 (females) g/day (ref.) | | | 1213 | | | 833 | | | 13.2 | | | 0.71 (0.48-1.04) | | |  | | | 9 | | | Sex, birth cohort (included as a stratification variable), smoking status, BMI, education, marital status, physical activity, total energy intake | | | (35) |
| Virtanen et al. 2019 | | Finland | | | | 1984 | | | KIHD | | 42–60 | | | Male | | | 4-day food record | | | Q4: 50 g/day (energy-adj.)  Q1: 0 g/day (ref.)* | | | 2641 | | | 1225 | | | 22.3 | | | 0.99 (0.83-1.17) | | | 0.91 | | | 8 | | | Age, examination year, energy intake, income, education years, marital status, leisure-time physical activity, pack-years of smoking, alcohol intake, BMI, diagnosis of type 2 diabetes, cardiovascular disease, cancer, or hypertension or use of cardiac, hypercholesterolemia, hypertension, or diabetes medications, intakes of fiber and saturated, monounsaturated, polyunsaturated, trans fatty acids | | | (4) |
| Ding et al. 2019 (NHS) | | USA | | | | 1976 | | | NHS, NHS II, HPFS | | 30–55 | | | Male & female (data shown for females) | | | FFQ | | | C4: > 32.7 g/day C1: < 30 g/week (ref.) | | | 74805 | | | 25182 | | | 40 | | | 1.09 (1.0-1.18) | | | 0.07 | | | 8 | | | Age, family history of CVD, history of cancer, family history of cardiovascular disease, baseline disease status (hypertension, hypercholesterolemia), baseline BMI, physical activity, alternate healthy eating index score, total energy intake, smoking status, alcohol consumption, postmenopausal status (only for females), current postmenopausal hormone use (only for females) | | | (36) |
| Ding et al. 2019 (HPFS) | | USA | | | | 1986 | | | NHS, NHS II, HPFS | | 40–75 | | | Male & female (data shown for males) | | | FFQ | | | C4: > 27.6 g/day C1: < 30 g/week (ref.) | | | 49602 | | | 23560 | | | 30 | | | 0.92 (0.85-0.99) | | | 0.04 | | | 8 | | | Age, family history of CVD, history of cancer, family history of cardiovascular disease, baseline disease status (hypertension, hypercholesterolemia), baseline BMI, physical activity, alternate healthy eating index score, total energy intake, smoking status, alcohol consumption, postmenopausal status (only for females), current postmenopausal hormone use (only for females) | | | (36) |
| Ding et al. 2019 (NHSII) | | USA | | | | 1989 | | | NHS, NHS II, HPFS | | 25–42 | | | Male & female (data shown for females) | | | FFQ | | | C4: > 36 g/day C1: < 30 g/week (ref.) | | | 93348 | | | 2696 | | | 27 | | | 1.2 (0.96-1.51) | | | 0.009 | | | 8 | | | Age, family history of CVD, history of cancer, family history of cardiovascular disease, baseline disease status (hypertension, hypercholesterolemia), baseline BMI, physical activity, alternate healthy eating index score, total energy intake, smoking status, alcohol consumption, postmenopausal status (only for females), current postmenopausal hormone use (only for females) | | | (36) |
| Pala et al. 2019 | | Italy | | | | 1993 | | | EPIC-Italy | | 45–64 | | | Male & female | | | FFQ | | | Q4: >100 g/day  Q1: 0 to ≤28 g/day (ref.) | | | 45009 | | | 2468 | | | 14.9 | | | 0.99 (0.84-1.16) | | | 0.72 | | | 8 | | | Region, sex, age, energy intake, weight, height, waist-to-hip ratio, alcohol consumption, smoking status, physical activity, relative index of inequality, Italian Mediterranean Index, intake of sugar | | | (16) |
| Mazidi et al. 2019 | | USA | | | | 1999 | | | NHANES | | > 20 | | | Male & female | | | 24-h recall | | | Q4: 4.6 oz natural cheese or 6.2 oz processed cheese Q1: 0.38 oz natural cheese or 0.5 oz processed cheese* | | | 24474 | | | 3520 | | | 6.4 | | | 0.92 (0.87-0.97) | | | 0.001 | | | 9 | | | Age, sex, race, education, marital status, poverty to income ratio, total energy intake, physical activity, smoking, alcohol consumption, carbohydrates, saturated fat, protein, dietary fiber, BMI, hypertension, diabetes | | | (17) |
| Sonestedt et al. 2021 | | Sweden | | | | 1991 | | | MDCS | | 45–73 | | | Male & female | | | SFFQ, food record, and interview | | | C6: >100 g/day C1: 0-20 g/day (ref.) | | | 26190 | | | 7156 | | | 19 | | | 0.83 (0.72-0.95) | | | 0.001 | | | 9 | | | Age, sex, diet assessment method, season, energy, BMI, education, physical activity, smoking, alcohol habits, diet (fruit and vegetables, meat, fiber, sugar-sweetened beverages) | | | (20) |
| Guo et al. 2022 | | Denmark | | | | 1982 | | | MONICA | | 30–60 | | | Male & female | | | 7-day weighed food record | | | Q4: 73.7 g/week  Q1: 6.3 g/week** | | | 1746 | | | 660 | | | 30 | | | 0.96 (0.76-1.22) | | | 0.46 | | | 8 | | | Sex, BMI, food energy intake, alcohol consumption, education, smoking, physical activity, family history of myocardial infarction, multivitamin use, serum total cholesterol, serum triaclyglycerols, incidence of hypertension | | | (5) |
| Lu et al. 2022 (Male) | | Japan | | | | 1990 | | | Miyagi Cohort | | 40–64 | | | Male & female (data shown for males) | | | FFQ | | | 3 times/week or almost daily  Almost never (ref.) | | | 16565 | | | 4354 | | | 25 | | | 1.05 (0.91-1.22) | | | 0.356 | | | 9 | | | Age (continuous), education level, BMI, smoking status, alcohol drinking status, history of hypertension, history of diabetes, energy intake, fish intake, vegetable and fruit intake | | | (22) |
| Lu et al. 2022 (Female) | | Japan | | | | 1990 | | | Miyagi Cohort | | 40–64 | | | Male & female (data shown for females) | | | FFQ | | | 3 times/week or almost daily  Almost never (ref.) | | | 17596 | | | 2522 | | | 25 | | | 0.89 (0.74-1.07) | | | 0.016 | | | 9 | | | Age (continuous), education level, BMI, smoking status, alcohol drinking status, history of hypertension, history of diabetes, energy intake, fish intake, vegetable and fruit intake | | | (22) |
| Ge et al. 2023 (Male) | | Japan | | | | 1995 | | | JPHC | | 40–69 | | | Male & female (data shown for males) | | | FFQ | | | Q4: 6.1 g/day (energy-adj.)  Q1: 0 g/day (ref.)* | | | 43117 | | | 14211 | | | 19.3 | | | 0.99 (0.94-1.04) | | | 0.79 | | | 7 | | | Age, study area, smoking status, alcohol frequency, BMI, physical activity, hypertension with medication, self, reported diabetes, green tea, coffee, energy-adjusted consumption of vegetables and fruits, total energy and total fat, menopausal status (only for females), exogenous hormone use (only for females), dairy intake | | | (23) |
| Ge et al. 2023 (Female) | | Japan | | | | 1995 | | | JPHC | | 40–69 | | | Male & female (data shown for females) | | | FFQ | | | Q4: 7.6 g/day (energy-adj.)  Q1: 0 g/day (ref.)* | | | 50193 | | | 9547 | | | 19.3 | | | 0.99 (0.92-1.05) | | | 0.68 | | | 7 | | | Age, study area, smoking status, alcohol frequency, BMI, physical activity, hypertension with medication, self, reported diabetes, green tea, coffee, energy-adjusted consumption of vegetables and fruits, total energy and total fat, menopausal status (only for females), exogenous hormone use (only for females), dairy intake | | | (23) |
| **Cheese / CVD** | | | | | | | | | | | | | | | | | | | | | | | | | | | | | | | | | | | | | | | | | | | | |
| Mann et al. 1997 | | UK | | | | 1980 | | | UK Health-Conscious Diet Cohort | | 16–79 | | | Male & female | | | SFFQ | | | C3: ≥ 5 times/week C1: < 1 times/week (ref.) | | | 10802 | | | 64 | | | 13.3 | | | 2.47 (0.97-6.26) | | | 0.01 | | | 6 | | | Age, sex, smoking, social class | | | (33) |
| Bonthuis et al. 2010 | | Australia | | | | 1992 | | | Nambour Skin Cancer Study | | 25–78 | | | Male & female | | | FFQ | | | Q3: 30 g/day  Q1: 4 g/day (ref.)* | | | 1529 | | | 61 | | | 14.4 | | | 0.64 (0.27-1.49) | | | 0.54 | | | 8 | | | Age, sex, total energy intake, BMI, alcohol intake, school leaving age, physical activity level, pack-years of smoking, dietary supplement use, beta-carotene treatment during trial, presence of any medical condition, dietary calcium | | | (8) |
| Goldbohm et al. 2011 (Female Stroke M.) | | Netherlands | | | | 1986 | | | NLCS | | 55–69 | | | Male & female (data shown for females) | | | FFQ | | | Q5/C5: 56 g/day  Q1/C1: 1 g/day (ref.)* | | | 62573 | | | 322 | | | 10 | | | 0.65 (0.39-1.1) | | | 0.169 | | | 8 | | | Age, education, smoking, physical activity, BMI, multivitamin use, alcohol, energy, energy-adjusted mono- and polyunsaturated fat intakes, vegetable and fruit consumption | | | (9) |
| Goldbohm et al. 2011 (Male Stroke M.) | | Netherlands | | | | 1986 | | | NLCS | | 55–69 | | | Male & female (data shown for males) | | | FFQ | | | Q5/C5: 56 g/day  Q1/C1: 1 g/day (ref.)* | | | 58279 | | | 520 | | | 10 | | | 1.11 (0.75-1.64) | | | 0.403 | | | 8 | | | Age, education, smoking, physical activity, BMI, multivitamin use, alcohol, energy, energy-adjusted mono- and polyunsaturated fat intakes, vegetable and fruit consumption | | | (9) |
| Goldbohm et al. 2011 (Female IHD M.) | | Netherlands | | | | 1986 | | | NLCS | | 55–69 | | | Male & female (data shown for females) | | | FFQ | | | Q5/C5: 56 g/day  Q1/C1: 1 g/day (ref.)* | | | 62573 | | | 692 | | | 10 | | | 1.01 (0.68-1.5) | | | 0.832 | | | 8 | | | Age, education, smoking, physical activity, BMI, multivitamin use, alcohol, energy, energy-adjusted mono- and polyunsaturated fat intakes, vegetable and fruit consumption | | | (9) |
| Goldbohm et al. 2011 (Male IHD M.) | | Netherlands | | | | 1986 | | | NLCS | | 55–69 | | | Male & female (data shown for males) | | | FFQ | | | Q5/C5: 56 g/day  Q1/C1: 1 g/day (ref.)* | | | 58279 | | | 1997 | | | 10 | | | 0.97 (0.74-1.27) | | | 0.639 | | | 8 | | | Age, education, smoking, physical activity, BMI, multivitamin use, alcohol, energy, energy-adjusted mono- and polyunsaturated fat intakes, vegetable and fruit consumption | | | (9) |
| van Aerde et al. 2013 | | Netherlands | | | | 1989 | | | The Hoorn Study | | 50–75 | | | Male & female | | | FFQ | | | Q4: ≥14.7 g/day Q1: ≤6.0 g/day (ref.)* | | | 1956 | | | 116 | | | 12.4 | | | 1.09 (0.87-1.35) | | | 0.46 | | | 8 | | | Age, sex, BMI, smoking, educational level, total energy intake, alcohol consumption, physical activity, intake of meat, fish, bread, vegetables, fruit, coffee, tea | | | (3) |
| Praagman et al. 2015 | | Netherlands | | | | 1993 | | | EPIC-NL | | 20–70 | | | Male & female | | | FFQ | | | Q4: 53.2 g/day (energy-adj.)  Q1: 6.6 g/day (ref.)* | | | 34409 | | | 727 | | | 15 | | | 0.8 (0.65-0.99) | | | 0.1 | | | 8 | | | Age, sex, smoking habit, BMI, physical activity, education level, hypertension at baseline, intakes of alcohol and energy-adjusted intakes of fruit and vegetables, total energy intake | | | (11) |
| Praagman et al. 2015 (CHD M.) | | Netherlands | | | | 1990 | | | Rotterdam Study | | ≥ 55 | | | Male & female | | | SFFQ | | | T3: >40 g/day  T1: <20 g/day (ref.)* | | | 4235 | | | 350 | | | 17.3 | | | 1.18 (0.86-1.64) | | | 0.36 | | | 8 | | | Age, sex, total energy intake, BMI, smoking, education level, alcohol intake, intakes of vegetables, fruit, meat, bread, fish coffee, tea | | | (6) |
| Praagman et al. 2015 (Stroke M.) | | Netherlands | | | | 1990 | | | Rotterdam Study | | ≥ 55 | | | Male & female | | | SFFQ | | | T3: >40 g/day  T1: <20 g/day (ref.)* | | | 4235 | | | 182 | | | 17.3 | | | 1.07 (0.7-1.64) | | | 0.79 | | | 8 | | | Age, sex, total energy intake, BMI, smoking, education level, alcohol intake, intakes of vegetables, fruit, meat, bread, fish coffee, tea | | | (6) |
| Farvid et al. 2017 | | Iran | | | | 2004 | | | Golestan Study | | 36–85 | | | Male & female | | | FFQ | | | Q5: 0.8 servings/day  Q1: 0 servings/day (ref.)** | | | 42403 | | | 1467 | | | 8 | | | 0.74 (0.61-0.91) | | | 0.02 | | | 8 | | | Age, ethnicity, education, marital status, residency, smoking, opium use, alcohol use, BMI, systolic blood pressure, occupational physical activity, family history of cancer, wealth score, medication use, energy intake | | | (14) |
| Ding et al. 2019 (NHS) | | USA | | | | 1976 | | | NHS, NHS II, HPFS | | 30–55 | | | Male & female (data shown for females) | | | FFQ | | | C4: > 34.5 g/day C1: < 30 g/week (ref.) | | | 74805 | | | 4418 | | | 40 | | | 1.15 (0.95-1.39) | | | 0.23 | | | 8 | | | Age, family history of CVD, history of cancer, family history of cardiovascular disease, baseline disease status (hypertension, hypercholesterolemia), baseline BMI, physical activity, alternate healthy eating index score, total energy intake, smoking status, alcohol consumption, postmenopausal status (only for females), current postmenopausal hormone use (only for females) | | | (36) |
| Ding et al. 2019 (HPFS) | | USA | | | | 1986 | | | NHS, NHS II, HPFS | | 40–75 | | | Male & female (data shown for males) | | | FFQ | | | C4: > 34.8 g/day C1: < 30 g/week (ref.) | | | 49602 | | | 7467 | | | 30 | | | 0.86 (0.75-0.99) | | | 0.05 | | | 8 | | | Age, family history of CVD, history of cancer, family history of cardiovascular disease, baseline disease status (hypertension, hypercholesterolemia), baseline BMI, physical activity, alternate healthy eating index score, total energy intake, smoking status, alcohol consumption, postmenopausal status (only for females), current postmenopausal hormone use (only for females) | | | (36) |
| Ding et al. 2019 (NHSII) | | USA | | | | 1989 | | | NHS, NHS II, HPFS | | 25–42 | | | Male & female (data shown for females) | | | FFQ | | | C4: > 42 g/day C1: < 30 g/week (ref.) | | | 93348 | | | 258 | | | 27 | | | 1.4 (0.7-2.79) | | | 0.01 | | | 8 | | | Age, family history of CVD, history of cancer, family history of cardiovascular disease, baseline disease status (hypertension, hypercholesterolemia), baseline BMI, physical activity, alternate healthy eating index score, total energy intake, smoking status, alcohol consumption, postmenopausal status (only for females), current postmenopausal hormone use (only for females) | | | (36) |
| Pala et al. 2019 | | Italy | | | | 1993 | | | EPIC-Italy | | 45–64 | | | Male & female | | | FFQ | | | Q4: >100 g/day  Q1: 0 to ≤28 g/day (ref.) | | | 45009 | | | 459 | | | 14.9 | | | 0.88 (0.6-1.3) | | | 0.61 | | | 8 | | | Region, sex, age, energy intake, weight, height, waist-to-hip ratio, alcohol consumption, smoking status, physical activity, relative index of inequality, Italian Mediterranean Index, intake of sugar | | | (16) |
| Mazidi et al. 2019 | | USA | | | | 1999 | | | NHANES | | > 20 | | | Male & female | | | 24-h recall | | | Q4: 4.6 oz natural cheese or 6.2 oz processed cheese Q1: 0.38 oz natural cheese or 0.5 oz processed cheese* | | | 24474 | | | 709 | | | 6.4 | | | 1.04 (0.88-1.11) | | | 0.723 | | | 9 | | | Age, sex, race, education, marital status, poverty to income ratio, total energy intake, physical activity, smoking, alcohol consumption, carbohydrates, saturated fat, protein, dietary fiber, BMI, hypertension, diabetes | | | (17) |
| Lu et al. 2022 (Male) | | Japan | | | | 1990 | | | Miyagi Cohort | | 40–64 | | | Male & female (data shown for males) | | | FFQ | | | 3 times/week or almost daily  Almost never (ref.) | | | 16565 | | | 1048 | | | 25 | | | 1.01 (0.75-1.34) | | | 0.136 | | | 9 | | | Age (continuous), education level, BMI, smoking status, alcohol drinking status, history of hypertension, history of diabetes, energy intake, fish intake, vegetable and fruit intake | | | (22) |
| Lu et al. 2022 (Female) | | Japan | | | | 1990 | | | Miyagi Cohort | | 40–64 | | | Male & female (data shown for females) | | | FFQ | | | 3 times/week or almost daily  Almost never (ref.) | | | 17596 | | | 645 | | | 25 | | | 0.99 (0.7-1.41) | | | 0.634 | | | 9 | | | Age (continuous), education level, BMI, smoking status, alcohol drinking status, history of hypertension, history of diabetes, energy intake, fish intake, vegetable and fruit intake | | | (22) |
| Ge et al. 2023 (Male) | | Japan | | | | 1995 | | | JPHC | | 40–69 | | | Male & female (data shown for males) | | | FFQ | | | Q4: 6.1 g/day (energy-adj.)  Q1: 0 g/day (ref.)* | | | 43117 | | | 3379 | | | 19.3 | | | 0.87 (0.78-0.97) | | | 0.04 | | | 7 | | | Age, study area, smoking status, alcohol frequency, BMI, physical activity, hypertension with medication, self, reported diabetes, green tea, coffee, energy-adjusted consumption of vegetables and fruits, total energy and total fat, menopausal status (only for females), exogenous hormone use (only for females), dairy intake | | | (23) |
| Ge et al. 2023 (Female) | | Japan | | | | 1995 | | | JPHC | | 40–69 | | | Male & female (data shown for females) | | | FFQ | | | Q4: 7.6 g/day (energy-adj.)  Q1: 0 g/day (ref.)* | | | 50193 | | | 2582 | | | 19.3 | | | 0.91 (0.8-1.04) | | | 0.43 | | | 7 | | | Age, study area, smoking status, alcohol frequency, BMI, physical activity, hypertension with medication, self, reported diabetes, green tea, coffee, energy-adjusted consumption of vegetables and fruits, total energy and total fat, menopausal status (only for females), exogenous hormone use (only for females), dairy intake | | | (23) |
| **Cheese / Cancer** | | | | | | | | | | | | | | | | | | | | | | | | | | | | | | | | | | | | | | | | | | | | |
| Khan et al. 2004 (Male) | | Japan | | | | 1984 | | | Hokkaido Study | | 40–97 | | | Male & female (data shown for males) | | | FFQ | | | C5: several times per week, everyday  C1: never, several times per year, several times per month (ref.) | | | 1524 | | | 155 | | | 13.8 | | | 1 (0.7-1.7) | | |  | | | 7 | | | Age, health status, health education, health screening, smoking | | | (26) |
| Khan et al. 2004 (Female) | | Japan | | | | 1984 | | | Hokkaido Study | | 40–97 | | | Male & female (data shown for females) | | | FFQ | | | C5: several times per week, everyday  C1: never, several times per year, several times per month (ref.) | | | 1634 | | | 89 | | | 14.8 | | | 1.1 (0.6-2.2) | | |  | | | 7 | | | Age, health status, health education, health screening, smoking | | | (26) |
| Bonthuis et al. 2010 | | Australia | | | | 1992 | | | Nambour Skin Cancer Study | | 25–78 | | | Male & female | | | FFQ | | | Q3: 30 g/day  Q1: 4 g/day (ref.) | | | 1529 | | | 58 | | | 14.4 | | | No association between cheese intake and Cancer mortality (Data are not provided). (nan-nan) | | | | | | 8 | | | Age, sex, total energy intake, BMI, alcohol intake, school leaving age, physical activity level, pack-years of smoking, dietary supplement use, beta-carotene treatment during trial, presence of any medical condition, dietary calcium | | | (8) |
| Praagman et al. 2015 | | Netherlands | | | | 1993 | | | EPIC-NL | | 20–70 | | | Male & female | | | FFQ | | | Q4: 53.2 g/day (energy-adj.)  Q1: 6.6 g/day (ref.)* | | | 34409 | | | 1216 | | | 15 | | | 1.11 (0.94-1.3) | | | 0.3 | | | 8 | | | Age, sex, smoking habit, BMI, physical activity, education level, hypertension at baseline, intakes of alcohol and energy-adjusted intakes of fruit and vegetables, total energy intake | | | (11) |
| Farvid et al. 2017 | | Iran | | | | 2004 | | | Golestan Study | | 36–85 | | | Male & female | | | FFQ | | | Q5: 0.8 servings/day  Q1: 0 servings/day (ref.)** | | | 42403 | | | 859 | | | 8 | | | 0.98 (0.75-1.29) | | | 0.99 | | | 8 | | | Age, ethnicity, education, marital status, residency, smoking, opium use, alcohol use, BMI, systolic blood pressure, occupational physical activity, family history of cancer, wealth score, medication use, energy intake | | | (14) |
| Ding et al. 2019 (NHS) | | USA | | | | 1976 | | | NHS, NHS II, HPFS | | 30–55 | | | Male & female (data shown for females) | | | FFQ | | | C4: > 33.9 g/day C1: < 30 g/week (ref.) | | | 74805 | | | 7641 | | | 40 | | | 1.13 (0.98-1.3) | | | 0.01 | | | 8 | | | Age, family history of CVD, history of cancer, family history of cardiovascular disease, baseline disease status (hypertension, hypercholesterolemia), baseline BMI, physical activity, alternate healthy eating index score, total energy intake, smoking status, alcohol consumption, postmenopausal status (only for females), current postmenopausal hormone use (only for females) | | | (36) |
| Ding et al. 2019 (NHSII) | | USA | | | | 1989 | | | NHS, NHS II, HPFS | | 25–42 | | | Male & female (data shown for females) | | | FFQ | | | C4: > 32.7 g/day C1: < 30 g/week (ref.) | | | 93348 | | | 1157 | | | 27 | | | 1.09 (0.76-1.55) | | | 0.41 | | | 8 | | | Age, family history of CVD, history of cancer, family history of cardiovascular disease, baseline disease status (hypertension, hypercholesterolemia), baseline BMI, physical activity, alternate healthy eating index score, total energy intake, smoking status, alcohol consumption, postmenopausal status (only for females), current postmenopausal hormone use (only for females) | | | (36) |
| Ding et al. 2019 (HPFS) | | USA | | | | 1986 | | | NHS, NHS II, HPFS | | 40–75 | | | Male & female (data shown for males) | | | FFQ | | | C4: > 27.3 g/day C1: < 30 g/week (ref.) | | | 49602 | | | 6322 | | | 30 | | | 0.91 (0.78-1.07) | | | 0.39 | | | 8 | | | Age, family history of CVD, history of cancer, family history of cardiovascular disease, baseline disease status (hypertension, hypercholesterolemia), baseline BMI, physical activity, alternate healthy eating index score, total energy intake, smoking status, alcohol consumption, postmenopausal status (only for females), current postmenopausal hormone use (only for females) | | | (36) |
| Pala et al. 2019 | | Italy | | | | 1993 | | | EPIC-Italy | | 45–64 | | | Male & female | | | FFQ | | | Q4: >100 g/day  Q1 : 0 to ≤28 g/day (ref.) | | | 45009 | | | 1456 | | | 14.9 | | | 1.08 (0.88-1.32) | | | 0.77 | | | 8 | | | Region, sex, age, energy intake, weight, height, waist-to-hip ratio, alcohol consumption, smoking status, physical activity, relative index of inequality, Italian Mediterranean Index, intake of sugar | | | (16) |
| Mazidi et al. 2019 | | USA | | | | 1999 | | | NHANES | | > 20 | | | Male & female | | | 24-h recall | | | Q4: 4.6 oz natural cheese or 6.2 oz processed cheese Q1: 0.38 oz natural cheese or 0.5 oz processed cheese* | | | 24474 | | | 827 | | | 6.4 | | | 0.99  (0.98-1.02) | | | 0.852 | | | 9 | | | Age, sex, race, education, marital status, poverty to income ratio, total energy intake, physical activity, smoking, alcohol consumption, carbohydrates, saturated fat, protein, dietary fiber, BMI, hypertension, diabetes | | | (17) |
| Lu et al. 2022 (Male) | | Japan | | | | 1990 | | | Miyagi Cohort | | 40–64 | | | Male & female (data shown for males) | | | FFQ | | | 3 times/week or almost daily  Almost never (ref.) | | | 16565 | | | 1713 | | | 25 | | | 1.08 (0.85-1.36) | | | 0.14 | | | 9 | | | Age (continuous), education level, BMI, smoking status, alcohol drinking status, history of hypertension, history of diabetes, energy intake, fish intake, vegetable and fruit intake | | | (22) |
| Lu et al. 2022 (Female) | | Japan | | | | 1990 | | | Miyagi Cohort | | 40–64 | | | Male & female (data shown for females) | | | FFQ | | | 3 times/week or almost daily  Almost never (ref.) | | | 17596 | | | 839 | | | 25 | | | 0.95 (0.7-1.31) | | | 0.923 | | | 9 | | | Age (continuous), education level, BMI, smoking status, alcohol drinking status, history of hypertension, history of diabetes, energy intake, fish intake, vegetable and fruit intake | | | (22) |
| Ge et al. 2023 (Male) | | Japan | | | | 1995 | | | JPHC | | 40–69 | | | Male & female (data shown for males) | | | FFQ | | | Q4: 6.1 g/day (energy-adj.)  Q1: 0 g/day (ref.)* | | | 43117 | | | 5364 | | | 19.3 | | | 1.07 (0.98-1.16) | | | 0.11 | | | 7 | | | Age, study area, smoking status, alcohol frequency, BMI, physical activity, hypertension with medication, self, reported diabetes, green tea, coffee, energy-adjusted consumption of vegetables and fruits, total energy and total fat, menopausal status (only for females), exogenous hormone use (only for females), dairy intake | | | (23) |
| Ge et al. 2023 (Female) | | Japan | | | | 1995 | | | JPHC | | 40–69 | | | Male & female (data shown for females) | | | FFQ | | | Q4: 7.6 g/day (energy-adj.)  Q1: 0 g/day (ref.)* | | | 50193 | | | 3076 | | | 19.3 | | | 1.1 (0.98-1.22) | | | 0.06 | | | 7 | | | Age, study area, smoking status, alcohol frequency, BMI, physical activity, hypertension with medication, self, reported diabetes, green tea, coffee, energy-adjusted consumption of vegetables and fruits, total energy and total fat, menopausal status (only for females), exogenous hormone use (only for females), dairy intake | | | (23) |
| **Cheese / GI cancer** | | | | | | | | | | | | | | | | | | | | | | | | | | | | | | | | | | | | | | | | | | | | |
| Kojima et al. 2004 (Male Colon CA) | | Japan | | | | 1988 | | | JACC | | 40–79 | | | Male & female (data shown for males) | | | FFQ | | | 1–7 times/week  Seldom (ref.) | | | 45181 | | | 138 | | | 9.9 | | | 1.17 (0.68-2.01) | | | 0.53 | | | 8 | | | Age, family history of colorectal cancer, BMI, frequency of alcohol intake, current smoking status, walking time per day, educational level | | | (28) |
| Kojima et al. 2004 (Female Colon CA) | | Japan | | | | 1988 | | | JACC | | 40–79 | | | Male & female (data shown for females) | | | FFQ | | | 1–7 times/week  Seldom (ref.) | | | 62643 | | | 146 | | | 9.9 | | | 1.01 (0.61-1.69) | | | 0.98 | | | 8 | | | Age, family history of colorectal cancer, BMI, frequency of alcohol intake, current smoking status, walking time per day, educational level | | | (28) |
| Kojima et al. 2004 (Male Rectal CA) | | Japan | | | | 1988 | | | JACC | | 40–79 | | | Male & female (data shown for males) | | | FFQ | | | 1–7 times/week  Seldom (ref.) | | | 45181 | | | 116 | | | 9.9 | | | 1.19 (0.7-2.02) | | | 0.38 | | | 8 | | | Age, family history of colorectal cancer, BMI, frequency of alcohol intake, current smoking status, walking time per day, educational level | | | (28) |
| Kojima et al. 2004 (Female Rectal CA) | | Japan | | | | 1988 | | | JACC | | 40–79 | | | Male & female (data shown for females) | | | FFQ | | | 1–7 times/week  Seldom (ref.) | | | 62643 | | | 57 | | | 9.9 | | | 2.52 (1.11-5.72) | | | 0.07 | | | 8 | | | Age, family history of colorectal cancer, BMI, frequency of alcohol intake, current smoking status, walking time per day, educational level | | | (28) |
| Khan et al. 2004 (Male Stomach CA) | | Japan | | | | 1984 | | | Hokkaido Study | | 40–97 | | | Male & female (data shown for males) | | | FFQ | | | C5: several times per week, everyday  C1: never, several times per year, several times per month (ref.) | | | 1524 | | | 36 | | | 13.8 | | | 1.2 (0.5-3.0) | | |  | | | 7 | | | Age, health status, health education, health screening, smoking | | | (26) |
| Khan et al. 2004 (Female Stomach CA) | | Japan | | | | 1984 | | | Hokkaido Study | | 40–97 | | | Male & female (data shown for females) | | | FFQ | | | C5: several times per week, everyday  C1: never, several times per year, several times per month (ref.) | | | 1634 | | | 15 | | | 14.8 | | | 1.2 (0.3-5.4) | | |  | | | 7 | | | Age, health status, health education, health screening, smoking | | | (26) |
| Khan et al. 2004 (Male Pancreatic CA) | | Japan | | | | 1984 | | | Hokkaido Study | | 40–97 | | | Male & female (data shown for males) | | | FFQ | | | C5: several times per week, everyday  C1: never, several times per year, several times per month (ref.) | | | 1524 | | | 12 | | | 13.8 | | | 0.6 (0.1-4.4) | | |  | | | 7 | | | Age, health status, health education, health screening, smoking | | | (26) |
| Khan et al. 2004 (Female Pancreatic CA) | | Japan | | | | 1984 | | | Hokkaido Study | | 40–97 | | | Male & female (data shown for females) | | | FFQ | | | C5: several times per week, everyday  C1: never, several times per year, several times per month (ref.) | | | 1634 | | | 13 | | | 14.8 | | | 1.7 (0.4-7.9) | | |  | | | 7 | | | Age, health status, health education, health screening, smoking | | | (26) |
| Khan et al. 2004 (Female Colorectal CA) | | Japan | | | | 1984 | | | Hokkaido Study | | 40–97 | | | Male & female (data shown for females) | | | FFQ | | | C5: several times per week, everyday  C1: never, several times per year, several times per month (ref.) | | | 1634 | | | 14 | | | 14.8 | | | 1.5 (0.3-6.8) | | |  | | | 7 | | | Age, health status, health education, health screening, smoking | | | (26) |
| Tokui et al. 2022 (Male Stomach CA) | | Japan | | | | 1988 | | | JACC | | 40–79 | | | Male & female (data shown for males) | | | FFQ | | | >1 times/day  None (ref.) | | | 46465 | | | 574 | | | 9.9 | | | 0.79 (0.39-1.61) | | | 0.64 | | | 6 | | | Age | | | (29) |
| Tokui et al. 2022 (Female Stomach CA) | | Japan | | | | 1988 | | | JACC | | 40–79 | | | Male & female (data shown for females) | | | FFQ | | | >1 times/day  None (ref.) | | | 64327 | | | 285 | | | 9.9 | | | 1.18 (0.52-2.69) | | | 0.8 | | | 6 | | | Age | | | (29) |
| **Cheese / Lung cancer** | | | | | | | | | | | | | | | | | | | | | | | | | | | | | | | | | | | | | | | | | | | | |
| Ozasa et al. 2001 (Male) | | Japan | | | | 1988 | | | JACC | | 40–79 | | | Male & female (data shown for males) | | | FFQ | | | Q3: >3 dishes/day Q1: <1 dishes/day (ref.) | | | 42940 | | | 446 | | | 7.7 | | | 0.59 (0.38-0.91) | | | 0.0029 | | | 8 | | | Age, parents' history of lung cancer, smoking status, smoking index, time since quitting smoking | | | (30) |
| Ozasa et al. 2001 (Female) | | Japan | | | | 1988 | | | JACC | | 40–79 | | | Male & female (data shown for females) | | | FFQ | | | Q3: >3 dishes/day Q1: <1 dishes/day (ref.) | | | 55308 | | | 126 | | | 7.7 | | | 0.81 (0.39-1.66) | | | 0.33 | | | 8 | | | Age, parents' history of lung cancer, smoking status, smoking index, time since quitting smoking | | | (30) |
| Khan et al. 2004 (Male) | | Japan | | | | 1984 | | | Hokkaido Study | | 40–97 | | | Male & female (data shown for males) | | | FFQ | | | C5: several times per week, everyday  C1: never, several times per year, several times per month (ref.) | | | 1524 | | | 41 | | | 13.8 | | | 1.6 (0.8-3.4) | | |  | | | 7 | | | Age, health status, health education, health screening, smoking | | | (26) |
| Khan et al. 2004 (Female) | | Japan | | | | 1984 | | | Hokkaido Study | | 40–97 | | | Male & female (data shown for females) | | | FFQ | | | C5: several times per week, everyday  C1: never, several times per year, several times per month (ref.) | | | 1634 | | | 10 | | | 14.8 | | | 1.1 (0.1-8.8) | | |  | | | 7 | | | Age, health status, health education, health screening, smoking | | | (26) |
| **Cheese / Reproductive cancer** | | | | | | | | | | | | | | | | | | | | | | | | | | | | | | | | | | | | | | | | | | | | |
| Mills et al. 1988 | | USA | | | | 1960 | | | AHS | | 30–85 | | | Female | | | FFQ | | | C4: >3 days/week C1: none/occasional (ref.) | | | 16190 | | | 142 | | | 20 | | | 1.25 (0.6-2.61) | | | 0.98 | | | 8 | | | Age at menarche, age at first pregnancy, age at menopause, percent desirable weight, education, consumption of other animal products | | | (37) |
| Park et al. 2007 | | USA | | | | 1995 | | | NIH-AARP | | 50–71 | | | Male | | | FFQ | | | C5: > 3 servings/day  C1: < 0.5 servings/day (ref.) | | | 293888 | | | 178 | | | 6 | | | 1.24 (0.56-2.75) | | | 0.74 | | | 7 | | | Age, race/ethnicity, education, marital status, BMI, physical activity, smoking, alcohol consumption, history of diabetes, family history of prostate cancer, PSA screening, dietary calcium, energy intake | | | (31) |
| Sakauchi et al. 2007 | | Japan | | | | 1988 | | | JACC | | 40–79 | | | Female | | | FFQ | | | Q3: ≥1–2 times/week Q1: seldom (ref.) | | | 63541 | | | 77 | | | 13.3 | | | 1.66 (0.65-4.25) | | | 0.27 | | | 7 | | | Age, menopausal status, number of pregnancies, history of sex hormone use, BMI, physical activity, education | | | (32) |
| **Study & subgroup details** | | | **Region** | **Start year** | | | **Cohort** | | | **Age range at entry** | | | **Sex** | | | **Dietary assessm.** | | | **Exposure levels** | | | **No. of subjects** | | | **No. of deaths** | | | **Years of FU** | | | **HR (95% CI)** | | | **p-value** | | | **NOS** | | | **Adjustments** | | | **Ref.** | |
| **Miso** | | | | | | | | | | | | | | | | | | | | | | | | | | | | | | | | | | | | | | | | | | | | |
| **Miso / All-causes** | | | | | | | | | | | | | | | | | | | | | | | | | | | | | | | | | | | | | | | | | | | | |
| Katagiri et al. 2020 (Male) | | | Japan | 1990 | | | JPHC | | | 45–74 | | | Male & female (data shown for males) | | | FFQ | | | Q5: >31.1 g/day (energy-adj.) Q1: <7.7 g/day (ref.) | | | 42750 | | | 8370 | | | 14.8 | | | 0.95 (0.87-1.02) | | | 0.2 | | | 8 | | | Age, geographical area, smoking, frequency of alcohol intake, BMI, sports or physical exercise, history of diabetes or taking drugs for diabetes, taking antihypertensives, health check-up, total energy intake, intake of green tea, coffee, fish, meat, fruit, vegetables | | | (38) | |
| Katagiri et al. 2020 (Female) | | | Japan | 1990 | | | JPHC | | | 45–74 | | | Male & female (data shown for females) | | | FFQ | | | Q5: >26.3 g/day (energy-adj.) Q1: <6.4 g/day (ref.) | | | 50165 | | | 4933 | | | 14.8 | | | 0.89 (0.81-0.97) | | | 0.03 | | | 8 | | | Age, geographical area, smoking, frequency of alcohol intake, BMI, sports or physical exercise, history of diabetes or taking drugs for diabetes, taking antihypertensives, health check-up, total energy intake, intake of green tea, coffee, fish, meat, fruit, vegetables | | | (38) | |
| **Miso / CVD** | | | | | | | | | | | | | | | | | | | | | | | | | | | | | | | | | | | | | | | | | | | | |
| Katagiri et al. 2020 (Male) | | | Japan | 1990 | | | JPHC | | | 45–74 | | | Male & female (data shown for males) | | | FFQ | | | Q5: >31.1 g/day (energy-adj.) Q1: <7.7 g/day (ref.) | | | 42750 | | | 2000 | | | 14.8 | | | 0.95 (0.82-1.1) | | | 0.84 | | | 8 | | | Age, geographical area, smoking, frequency of alcohol intake, BMI, sports or physical exercise, history of diabetes or taking drugs for diabetes, taking antihypertensives, health check-up, total energy intake, intake of green tea, coffee, fish, meat, fruit, vegetables | | | (38) | |
| Katagiri et al. 2020 (Female) | | | Japan | 1990 | | | JPHC | | | 45–74 | | | Male & female (data shown for females) | | | FFQ | | | Q5: >26.3 g/day (energy-adj.) Q1: <6.4 g/day (ref.) | | | 50165 | | | 1326 | | | 14.8 | | | 0.94 (0.79-1.13) | | | 0.86 | | | 8 | | | Age, geographical area, smoking, frequency of alcohol intake, BMI, sports or physical exercise, history of diabetes or taking drugs for diabetes, taking antihypertensives, health check-up, total energy intake, intake of green tea, coffee, fish, meat, fruit, vegetables | | | (38) | |
| **Miso / Cancer** | | | | | | | | | | | | | | | | | | | | | | | | | | | | | | | | | | | | | | | | | | | | |
| Khan et al. 2004 | | | Japan | 1984 | | | Hokkaido Study | | | 40–97 | | | Male & female (data shown for males) | | | FFQ | | | C5: several times per week, everyday  C1: never, several times per year, several times per month (ref.) | | | 1524 | | | 155 | | | 13.8 | | | 0.4 (0.1-1.1) | | | | | | 7 | | | Age, health status, health education, health screening, smoking | | | (26) | |
| Katagiri et al. 2020 (Male) | | | Japan | 1990 | | | JPHC | | | 45–74 | | | Male & female (data shown for males) | | | FFQ | | | Q5: >31.1 g/day (energy-adj.) Q1: <7.7 g/day (ref.) | | | 42750 | | | 3320 | | | 14.8 | | | 1.02 (0.91-1.16) | | | 0.74 | | | 8 | | | Age, geographical area, smoking, frequency of alcohol intake, BMI, sports or physical exercise, history of diabetes or taking drugs for diabetes, taking antihypertensives, health check-up, total energy intake, intake of green tea, coffee, fish, meat, fruit, vegetables | | | (38) | |
| Katagiri et al. 2020 (Female) | | | Japan | 1990 | | | JPHC | | | 45–74 | | | Male & female (data shown for females) | | | FFQ | | | Q5: >26.3 g/day (energy-adj.) Q1: <6.4 g/day (ref.) | | | 50165 | | | 1817 | | | 14.8 | | | 0.88 (0.75-1.03) | | | 0.23 | | | 8 | | | Age, geographical area, smoking, frequency of alcohol intake, BMI, sports or physical exercise, history of diabetes or taking drugs for diabetes, taking antihypertensives, health check-up, total energy intake, intake of green tea, coffee, fish, meat, fruit, vegetables | | | (38) | |
| **Miso / GI cancer** | | | | | | | | | | | | | | | | | | | | | | | | | | | | | | | | | | | | | | | | | | | | |
| Hirayama et al. 1981 (Male Gastric CA) | | | Japan | 1966 | | | Hirayama Cohort | | | ≥ 49 | | | Male & female (data shown for males) | | | Interview questionnaire | | | Daily intake  No intake (ref.) | | | 122261 | | | 2562 | | | 13 | | | 0.67 (0.56-0.8) | | | | | | 6 | | | Sex, age, smoking, occupation, residence, marital status, alcohol, rice, meat, fish, milk, pickles, green-yellow vegetables, hot green tea intake | | | (39) | |
| Hirayama et al. 1981 (Female Gastric CA) | | | Japan | 1966 | | | Hirayama Cohort | | | ≥ 49 | | | Male & female (data shown for females) | | | Interview questionnaire | | | Daily intake  No intake (ref.) | | | 142857 | | | 1351 | | | 13 | | | 0.69 (0.54-0.87) | | | | | | 6 | | | Sex, age, smoking, occupation, residence, marital status, alcohol, rice, meat, fish, milk, pickles, green-yellow vegetables, hot green tea intake | | | (39) | |
| Ngoan et al. 2002 (Male Stomach CA) | | | Japan | 1986 | | | Fukuoka Prefecture cohort | | | 15–96 | | | Male & female (data shown for males) | | | FFQ | | | C3: ≥ 2 times/day C1: ≤2-4 times/week (ref.) | | | 5917 | | | 77 | | | 13 | | | 1.4 (0.7-3.2) | | | | | | 5 | | | Age, sex | | | (40) | |
| Ngoan et al. 2002 (Female Stomach CA) | | | Japan | 1986 | | | Fukuoka Prefecture cohort | | | 20–92 | | | Male & female (data shown for females) | | | FFQ | | | C3: ≥ 2 times/day C1: ≤2-4 times/week (ref.) | | | 7333 | | | 39 | | | 13 | | | 0.7 (0.2-3.4) | | | | | | 5 | | | Age, sex | | | (40) | |
| Kurozawa et al. 2004 (Male Hepato CA) | | | Japan | 1988 | | | JACC | | | 40–59 | | | Male & female (data shown for males) | | | FFQ | | | Q3: 2- servings/day Q1: ≤ servings/day (ref.) | | | 46465 | | | 287 | | | 9.9 | | | 4.36 (0.99-19.33) | | | | | | 5 | | | No adjustment | | | (41) | |
| Kurozawa et al. 2004 (old Men Hepato CA) | | | Japan | 1988 | | | JACC | | | 60–79 | | | Male & female (data shown for males) | | | FFQ | | | Q3: 2- servings/day Q1: ≤ servings/day (ref.) | | | 46465 | | | 287 | | | 9.9 | | | 1.12 (0.43-2.91) | | | | | | 5 | | | No adjustment | | | (41) | |
| Kurozawa et al. 2004 (Female Hepato CA) | | | Japan | 1988 | | | JACC | | | 40–59 | | | Male & female (data shown for females) | | | FFQ | | | Q3: 2- servings/day Q1: ≤ servings/day (ref.) | | | 64327 | | | 114 | | | 9.9 | | | 0.31 (0.03-3.12) | | | | | | 5 | | | No adjustment | | | (41) | |
| Kurozawa et al. 2004 (old Female Hepato CA) | | | Japan | 1988 | | | JACC | | | 60–79 | | | Male & female (data shown for females) | | | FFQ | | | Q3: 2- servings/day Q1: ≤ servings/day (ref.) | | | 64327 | | | 114 | | | 9.9 | | | 0.17 (0.04-0.67) | | | 0 .05 | | | 5 | | | No adjustment | | | (41) | |
| Khan et al. 2004 (Stomach CA) | | | Japan | 1984 | | | Hokkaido Study | | | 40–97 | | | Male & female (data shown for males) | | | FFQ | | | C5: several times per week, everyday  C1: never, several times per year, several times per month (ref.) | | | 1524 | | | 36 | | | 13.8 | | | 0.2 (0.1-0.8) | | | 0.05 | | | 7 | | | Age, health status, health education, health screening, smoking | | | (26) | |
| Tokui et al. 2022 (Male Stomach CA) | | | Japan | 1988 | | | JACC | | | 40–79 | | | Male & female (data shown for males) | | | FFQ | | | >1 times/day  None (ref.) | | | 46465 | | | 574 | | | 9.9 | | | 1.44 (0.86-2.42) | | | 0.36 | | | 6 | | | Age | | | (29) | |
| Tokui et al. 2022 (Female Stomach CA) | | | Japan | 1988 | | | JACC | | | 40–79 | | | Male & female (data shown for females) | | | FFQ | | | >1 times/day  None (ref.) | | | 64327 | | | 285 | | | 9.9 | | | 1.46 (0.81-2.61) | | | 0.19 | | | 6 | | | Age | | | (29) | |
| **Study & subgroup details** | **Region** | | | | **Start year** | | | **Cohort** | | | | **Age range at entry** | | | **Sex** | | | **Dietary assessm.** | | | **Exposure levels** | | | **No. of subjects** | | | **No. of deaths** | | | **Years of FU** | | | **HR (95% CI)** | | | **p-value** | | | **NOS** | | | **Adjustments** | | **Ref.** |
| **Bread** | | | | | | | | | | | | | | | | | | | | | | | | | | | | | | | | | | | | | | | | | | | | |
| **Bread / All-causes** | | | | | | | | | | | | | | | | | | | | | | | | | | | | | | | | | | | | | | | | | | | | |
| Fortes et al. 2000 | Italy | | | | 1993 | | | Rome Elderly Cohort | | | | ≥ 65 | | | Male & female | | | FFQ | | | T3: > 3 times/week  T1: <1 times/week (ref.) | | | 161 | | | 53 | | | 5 | | | 0.65 (0.29-1.45) | | |  | | | 7 | | | No adjustments | | (34) |
| Bongard et al. 2016 | France | | | | 1995 | | | MONICA | | | | 45–64 | | | Male | | | 3-day food record | | | Q4: 460 g/day (energy-adj.)  Q1: 0 g/day (ref.)* | | | 960 | | | 150 | | | 14.8 | | | 0.8 (0.51-1.26) | | | 0.08 | | | 8 | | | Center, age, payment of income tax, obesity, alcohol consumption, smoking habits, physical activity, presence of a serious chronic condition, diet quality score | | (12) |
| **Bread / CVD** | | | | | | | | | | | | | | | | | | | | | | | | | | | | | | | | | | | | | | | | | | | | |
| Rebello et al. 2014 (Male Whole W.) | Singapore | | | | 1993 | | | SCHS | | | | 45–74 | | | Male & female (data shown for males) | | | SFFQ | | | Q3: >1 slices/day  Q1: 0 slices/day (ref.)* | | | 23501 | | | 1022 | | | 15 | | | 0.94 (0.66-1.33) | | | 0.095 | | | 9 | | | Age, dialect group, year of interview, energy intake, cigarette smoking, alcohol intake, physical activity, education, BMI, history of hypertension, use of hormone-replacement therapy (only for females), ratio of PUFAs to SFAs, cholesterol intake, fiber intake | | (42) |
| Rebello et al. 2014 (Female Whole W.) | Singapore | | | | 1993 | | | SCHS | | | | 45–74 | | | Male & female (data shown for females) | | | SFFQ | | | Q3: >1 slices/day  Q1: 0 slices/day (ref.)* | | | 29968 | | | 638 | | | 15 | | | 0.51 (0.3-0.89) | | | 0.01 | | | 9 | | | Age, dialect group, year of interview, energy intake, cigarette smoking, alcohol intake, physical activity, education, BMI, history of hypertension, use of hormone-replacement therapy (only for females), ratio of PUFAs to SFAs, cholesterol intake, fiber intake | | (42) |
| Rebello et al. 2014 (Male White W.) | Singapore | | | | 1993 | | | SCHS | | | | 45–74 | | | Male & female (data shown for males) | | | SFFQ | | | Q3: >1 slices/day  Q1: 0 slices/day (ref.)* | | | 23501 | | | 1022 | | | 15 | | | 1.12 (0.9-1.39) | | | 0.2 | | | 9 | | | Age, dialect group, year of interview, energy intake, cigarette smoking, alcohol intake, physical activity, education, BMI, history of hypertension, use of hormone-replacement therapy (only for females), ratio of PUFAs to SFAs, cholesterol intake, fiber intake | | (42) |
| Rebello et al. 2014 (Female White W.) | Singapore | | | | 1993 | | | SCHS | | | | 45–74 | | | Male & female (data shown for females) | | | SFFQ | | | Q3: >1 slices/day  Q1: 0 slices/day (ref.)* | | | 29968 | | | 638 | | | 15 | | | 0.79 (0.6-1.04) | | | 0.11 | | | 9 | | | Age, dialect group, year of interview, energy intake, cigarette smoking, alcohol intake, physical activity, education, BMI, history of hypertension, use of hormone-replacement therapy (only for females), ratio of PUFAs to SFAs, cholesterol intake, fiber intake | | (42) |
| Wada et al. 2022 (Male) | Japan | | | | 1993 | | | Takayama Study | | | | > 35 | | | Male & female (data shown for males) | | | FFQ | | | Q4: 76.7 g/day Q1: 2.9 g/day (ref.)* | | | 13355 | | | 779 | | | 14.1 | | | 0.92 (0.74-1.15) | | |  | | | 8 | | | Smoking status, physical activity, alcohol intake, coffee intake, salt Intake, marital status, education level, BMI, history of diabetes and hypertension, menopausal status (only for females) | | (43) |
| Wada et al. 2022 (Female) | Japan | | | | 1993 | | | Takayama Study | | | | > 35 | | | Male & female (data shown for females) | | | FFQ | | | Q4: 78.8 g/day Q1: 7.2 g/day (ref.)* | | | 15724 | | | 907 | | | 14.1 | | | 0.97 (0.8-1.19) | | |  | | | 8 | | | Smoking status, physical activity, alcohol intake, coffee intake, salt Intake, marital status, education level, BMI, history of diabetes and hypertension, menopausal status (only for females) | | (43) |
| **Bread / Lung cancer** | | | | | | | | | | | | | | | | | | | | | | | | | | | | | | | | | | | | | | | | | | | | |
| Chow et al. 1992 | USA | | | | 1966 | | | Lutheran Brotherhood Cohort | | | | ≥ 35 | | | Male | | | FFQ | | | Q4: > 240 times/month Q1: < 91 times/month (ref.) | | | 17633 | | | 219 | | | 20 | | | 1.0 (0.6-1.7) | | |  | | | 8 | | | Age, smoking status, industry/occupation | | (44) |
| Khan et al. 2004 (Male) | Japan | | | | 1984 | | | Hokkaido Study | | | | 40–97 | | | Male & female (data shown for males) | | | FFQ | | | C5: several times per week, everyday  C1: never, several times per year, several times per month (ref.) | | | 1524 | | | 41 | | | 13.8 | | | 0.7 (0.3-1.6) | | |  | | | 7 | | | Age, health status, health education, health screening, smoking | | (26) |
| Khan et al. 2004 (Female) | Japan | | | | 1984 | | | Hokkaido Study | | | | 40–97 | | | Male & female (data shown for females) | | | FFQ | | | C5: several times per week, everyday  C1: never, several times per year, several times per month (ref.) | | | 1634 | | | 10 | | | 14.8 | | | 0.3 (0.0-2.5) | | |  | | | 7 | | | Age, health status, health education, health screening, smoking | | (26) |
| **Choco&cocoa** | | | | | | | | | | | | | | | | | | | | | | | | | | | | | | | | | | | | | | | | | | | | |
| **Choco&cocoa / All-causes** | | | | | | | | | | | | | | | | | | | | | | | | | | | | | | | | | | | | | | | | | | | | |
| Paganini-Hill et al. 2007 | USA | | | | 1980 | | | Leisure World Cohort | | | | 44–101 | | | Male & female | | | FFQ | | | C5: few days/week to daily C1: rarely or never (ref.) | | | 13624 | | | 11386 | | | 23 | | | 0.98 (0.93-1.04) | | |  | | | 7 | | | Age, sex, smoking, exercise, BMI, alcohol intake, histories of hypertension, angina, heart attack, stroke, diabetes, rheumatoid arthritis, cancer | | (45) |
| Zhong et al. 2021 | USA | | | | 1993 | | | PLCO | | | | 55–74 | | | Male & female | | | FFQ | | | Q4: >2 servings/week (energy-adj.)  Q1: 0 servings/week (ref.)** | | | 91891 | | | 19586 | | | 13.5 | | | 0.87 (0.82-0.93) | | | 0.009 | | | 8 | | | Age, sex, ethnicity, educational level, marital status, study center, history of hypertension, history of diabetes, aspirin use, hormone use status (only for females), smoking status, alcohol consumption, BMI, physical activity, energy intake from diet, consumption of red meat, processed meat, fruit, vegetable, whole grain, dairy, coffee, tea | | (46) |
| Zhao et al. 2022 | Finland | | | | 1986 | | | ATBC | | | | 50–69 | | | Male | | | FFQ | | | Q5: 12.4 g/day (energy-adj.)  Q1: 0 g/day (ref.) | | | 27111 | | | 22064 | | | 31 | | | 0.88 (0.85-0.92) | | | 0.0001 | | | 8 | | | Age, BMI, energy intake, smoking, serum HDL and total cholesterol, intervention assignment, education, physical activity, alcohol consumption, Alternate MD score, systolic and diastolic blood pressure, history of cardiovascular disease and diabetes | | (47) |
| Sun et al. 2023 | USA | | | | 1993 | | | WHI | | | | 50–79 | | | Female | | | FFQ | | | Q5: ≥1 servings/day  Q1: 0 servings/week (ref.) | | | 84709 | | | 25388 | | | 19 | | | 0.9 (0.84-0.97) | | | 0.02 | | | 8 | | | Αge, race, ethnicity, education, annual family income, neighborhood-level socioeconomic status, observational study/clinical trial, unopposed estrogen use, estrogen and progesterone use, smoking status, physical activity, ΒΜΙ, alcohol intake, coffee or tea intake, total energy intake, baseline diabetes status, baseline high blood cholesterol status, family history of heart attack or stroke, Healthy Eating Index-2015 score | | (48) |
| **Choco&cocoa / CVD** | | | | | | | | | | | | | | | | | | | | | | | | | | | | | | | | | | | | | | | | | | | | |
| Buijsse et al. 2006 | Netherlands | | | | 1985 | | | Zutphen Elderly Study | | | | 65–84 | | | Male | | | Interview questionnaire | | | Q3: 4.18 g/day Q1: 0.0 g/day (ref.)* | | | 470 | | | 152 | | | 15 | | | 0.5 (0.32-0.78) | | | 0.004 | | | 9 | | | Age, BMI, alcohol intake, physical activity, smoking, diet prescription, aspirin use, anticoagulant use, physician in blood pressure, diet prescription, dietary cholesterol, intake of trans fatty acids, saturated fat, folic acid, vit C, vit E, beta-carotene, K, sodium, Ca and Mg, total calories | | (49) |
| Kwok et al. 2015 | UK | | | | 1993 | | | EPIC-Norfolk | | | | 40–79 | | | Male & female | | | FFQ | | | Q5: 15.6–98.8 g/day  Q1: 0 g/day (ref.)* | | | 20951 | | | 1107 | | | 11.3 | | | 0.75 (0.62-0.92) | | | 0.011 | | | 8 | | | Sex, age, smoking, physical activity, ΒΜΙ, energy intake, alcohol consumption, diabetes, systolic blood pressure, LDL cholesterol, HDL cholesterol | | (50) |
| Ho et al. 2021 | USA | | | | 2018 | | | Million Veteran Program | | | | 64 ± 12 | | | Male & female | | | FFQ | | | Q5: ≥5 times/week (141.7 g) Q1: <1 times/month (28.3 g) (ref.) | | | 188447 | | | 6946 | | | 3.2 | | | 0.89 (0.84-0.96) | | | 0.0001 | | | 5 | | | Age, sex, race, BMI, smoking, physical activity, alcohol consumption | | (51) |
| Zhong et al. 2021 | USA | | | | 1993 | | | PLCO | | | | 55–74 | | | Male & female | | | FFQ | | | Q4: >2 servings/week (energy-adj.)  Q1: 0 servings/week (ref.)** | | | 91891 | | | 5490 | | | 13.5 | | | 0.78 (0.7-0.88) | | | 0.02 | | | 8 | | | Age, sex, ethnicity, educational level, marital status, study center, history of hypertension, history of diabetes, aspirin use, hormone use status (only for females), smoking status, alcohol consumption, BMI, physical activity, energy intake from diet, consumption of red meat, processed meat, fruit, vegetable, whole grain, dairy, coffee, tea | | (46) |
| Zhao et al. 2022 | Finland | | | | 1986 | | | ATBC | | | | 50–69 | | | Male | | | FFQ | | | Q5: 12.4 g/day (energy-adj.)  Q1: 0 g/day (ref.) | | | 27111 | | | 9121 | | | 31 | | | 0.87 (0.82-0.94) | | | 0.0002 | | | 8 | | | Age, BMI, energy intake, smoking, serum HDL and total cholesterol, intervention assignment, education, physical activity, alcohol consumption, Alternate MD score, systolic and diastolic blood pressure, history of cardiovascular disease and diabetes | | (47) |
| Sun et al. 2023 | USA | | | | 1993 | | | WHI | | | | 50–79 | | | Female | | | FFQ | | | Q5: ≥1 servings/day  Q1: 0 servings/week (ref.) | | | 84709 | | | 7069 | | | 19 | | | 0.92 (0.8-1.05) | | |  | | | 8 | | | Αge, race, ethnicity, education, annual family income, neighborhood-level socioeconomic status, observational study/clinical trial, unopposed estrogen use, estrogen and progesterone use, smoking status, physical activity, ΒΜΙ, alcohol intake, coffee or tea intake, total energy intake, baseline diabetes status, baseline high blood cholesterol status, family history of heart attack or stroke, Healthy Eating Index-2015 score | | (48) |

*Median **Mean

CA, cancer; CI, confidence interval; CVD, cardiovascular disease; FFQ, food frequency questionnaire; FU, follow-up; HR, hazard ratio; NOS, Newcastle-Ottawa Scale; Ref., reference; SFFQ, semi-quantitative food frequency questionnaire; W., wheat.

# References

1. Paveljšek D, Pertziger E, Fardet A, Panagiotakos DB, Savary-Auzeloux I, Adamberg S, et al. A systematic review of prospective evidence linking non-alcoholic fermented food consumption with lower mortality risk. Front Nutr. 2025 Nov 3;12:1657100.

2. Soedamah-Muthu SS, Masset G, Verberne L, Geleijnse JM, Brunner EJ. Consumption of dairy products and associations with incident diabetes, CHD and mortality in the Whitehall II study. Br J Nutr. 2013 Feb 28;109(4):718–26.

3. Van Aerde MA, Soedamah-Muthu SS, Geleijnse JM, Snijder MB, Nijpels G, Stehouwer CDA, et al. Dairy intake in relation to cardiovascular disease mortality and all-cause mortality: the Hoorn study. Eur J Nutr. 2013 Mar;52(2):609–16.

4. Virtanen HE, Voutilainen S, Koskinen TT, Mursu J, Kokko P, Ylilauri MP, et al. Dietary proteins and protein sources and risk of death: the Kuopio Ischaemic Heart Disease Risk Factor Study. Am J Clin Nutr. 2019 May;109(5):1462–71.

5. Guo J, Givens DI, Heitmann BL. Association between dairy consumption and cardiovascular disease events, bone fracture and all-cause mortality. Huang HK, editor. PLoS ONE. 2022 Sept 9;17(9):e0271168.

6. Praagman J, Franco OH, Ikram MA, Soedamah-Muthu SS, Engberink MF, Van Rooij FJA, et al. Dairy products and the risk of stroke and coronary heart disease: the Rotterdam study. Eur J Nutr. 2015 Sept;54(6):981–90.

7. Silva FM, Giatti L, Diniz MDFHS, Brant LCC, Barreto SM. Dairy product consumption reduces cardiovascular mortality: results after 8 year follow-up of ELSA-Brasil. Eur J Nutr. 2022 Mar;61(2):859–69.

8. Bonthuis M, Hughes MCB, Ibiebele TI, Green AC, Van Der Pols JC. Dairy consumption and patterns of mortality of Australian adults. Eur J Clin Nutr. 2010 June;64(6):569–77.

9. Goldbohm RA, Chorus AM, Galindo Garre F, Schouten LJ, Van Den Brandt PA. Dairy consumption and 10-y total and cardiovascular mortality: a prospective cohort study in the Netherlands. Am J Clin Nutr. 2011 Mar;93(3):615–27.

10. Sluik D, Boeing H, Li K, Kaaks R, Johnsen NF, Tjønneland A, et al. Lifestyle factors and mortality risk in individuals with diabetes mellitus: are the associations different from those in individuals without diabetes? Diabetologia. 2014 Jan;57(1):63–72.

11. Praagman J, Dalmeijer GW, Van Der Schouw YT, Soedamah-Muthu SS, Monique Verschuren WM, Bas Bueno-de-Mesquita H, et al. The relationship between fermented food intake and mortality risk in the European Prospective Investigation into Cancer and Nutrition-Netherlands cohort. Br J Nutr. 2015 Feb 14;113(3):498–506.

12. Bongard V, Arveiler D, Dallongeville J, Ruidavets JB, Wagner A, Simon C, et al. Food groups associated with a reduced risk of 15-year all-cause death. Eur J Clin Nutr. 2016 June;70(6):715–22.

13. Tognon G, Nilsson LM, Shungin D, Lissner L, Jansson JH, Renström F, et al. Nonfermented milk and other dairy products: associations with all-cause mortality. Am J Clin Nutr. 2017 June;105(6):1502–11.

14. Farvid MS, Malekshah AF, Pourshams A, Poustchi H, Sepanlou SG, Sharafkhah M, et al. Dairy food intake and all-cause, cardiovascular disease, and cancer mortality. Am J Epidemiol. 2017 Apr 15;185(8):697–711.

15. Dehghan M, Mente A, Rangarajan S, Sheridan P, Mohan V, Iqbal R, et al. Association of dairy intake with cardiovascular disease and mortality in 21 countries from five continents (PURE): a prospective cohort study. The Lancet. 2018 Nov;392(10161):2288–97.

16. Pala V, Sieri S, Chiodini P, Masala G, Palli D, Mattiello A, et al. Associations of dairy product consumption with mortality in the European Prospective Investigation into Cancer and Nutrition (EPIC)–Italy cohort. Am J Clin Nutr. 2019 Nov;110(5):1220–30.

17. Mazidi M, Mikhailidis DP, Sattar N, Howard G, Graham I, Banach M. Consumption of dairy product and its association with total and cause specific mortality – a population-based cohort study and meta-analysis. Clin Nutr. 2019 Dec;38(6):2833–45.

18. Schmid D, Song M, Zhang X, Willett WC, Vaidya R, Giovannucci EL, et al. Yogurt consumption in relation to mortality from cardiovascular disease, cancer, and all causes: a prospective investigation in 2 cohorts of US women and men. Am J Clin Nutr. 2020 Mar;111(3):689–97.

19. Nakanishi A, Homma E, Osaki T, Sho R, Souri M, Sato H, et al. Association between milk and yogurt intake and mortality: a community-based cohort study (Yamagata study). BMC Nutr. 2021 Dec;7(1):33.

20. Sonestedt E, Borné Y, Wirfält E, Ericson U. Dairy consumption, lactase persistence, and mortality risk in a cohort from southern Sweden. Front Nutr. 2021 Nov 24;8:779034.

21. Lin P, Gui X, Liang Z, Wang T. Association of yogurt and dietary supplements containing probiotic consumption with all-cause and cause-specific mortality in US adults: a population-based cohort study. Front Nutr. 2022 Feb 7;9:803076.

22. Lu Y, Sugawara Y, Matsuyama S, Fukao A, Tsuji I. Association of dairy intake with all-cause, cancer, and cardiovascular disease mortality in Japanese adults: a 25-year population-based cohort. Eur J Nutr. 2022 Apr;61(3):1285–97.

23. Ge S, Zha L, Sobue T, Kitamura T, Iso H, Ishihara J, et al. Associations between dairy intake and mortality due to all-cause and cardiovascular disease: the Japan Public Health Center-based prospective study. Eur J Nutr. 2023 Aug;62(5):2087–104.

24. Miyagawa N, Takashima N, Harada A, Kadota A, Kondo K, Miura K, et al. Dairy intake and all-cause, cancer, and cardiovascular disease mortality risk in a large Japanese population: a 12-year follow-up of the J-MICC study. JAT. 2024;65049.

25. Zhang S, Li H, Engström G, Niu K, Qi L, Borné Y, et al. Milk intake, lactase persistence genotype, plasma proteins and risks of cardiovascular events in the Swedish general population. Eur J Epidemiol. 2023 Feb;38(2):211–24.

26. Khan M, Goto R, Kobayashi K, Suzumura S, Nagata Y, Sonoda T, et al. Dietary habits and cancer mortality among middle aged and older Japanese living in Hokkaido, Japan by cancer site and sex. APJCP. 2004;5:58–65.

27. Matsumoto M, Ishikawa S, Nakamura Y, Kayaba K, Kajii E. Consumption of dairy products and cancer risks. J Epidemiol. 2007;17(2):38–44.

28. Kojima M, Wakai K, Tamakoshi K, Tokudome S, Toyoshima H, Watanabe Y, et al. Diet and colorectal cancer mortality: results from the Japan collaborative cohort study. Nutr Cancer. 2004 Sept;50(1):23–32.

29. Tokui N, Yoshimura T, Fujino Y, Mizoue T, Hoshiyama Y, Yatsuya H, et al. Dietary habits and stomach cancer risk in the JACC study. J Epidemiol. 2005;15(Supplement_II):S98–108.

30. Ozasa K, Watanabe Y, Ito Y, Suzuki K, Tamakoshi A, Seki N, et al. Dietary habits and risk of lung cancer death in a large‐scale cohort study (JACC study) in Japan by sex and smoking habit. Japanese Journal of Cancer Research. 2001 Dec;92(12):1259–69.

31. Park Y, Mitrou PN, Kipnis V, Hollenbeck A, Schatzkin A, Leitzmann MF. Calcium, dairy foods, and risk of incident and fatal prostate cancer: the NIH-AARP diet and health study. Am J Epidemiol. 2007 Aug 28;166(11):1270–9.

32. Sakauchi F, Khan MMH, Mori M, Kubo T, Fujino Y, Suzuki S, et al. Dietary habits and risk of ovarian cancer death in a large-scale cohort study (JACCstudy) in Japan. Nutr Cancer. 2007 June 8;57(2):138–45.

33. Mann JI, Appleby PN, Key TJ, Thorogood M. Dietary determinants of ischaemic heart disease in health conscious individuals. Heart. 1997 Nov 1;78(5):450–5.

34. Fortes C, Forastiere F, Farchi S, Rapiti E, Pastori G, Perucci CA. Diet and overall survival in a cohort of very elderly people. Epidemiology. 2000;11(4):440–5.

35. Tognon G, Rothenberg E, Petrolo M, Sundh V, Lissner L. Dairy product intake and mortality in a cohort of 70-year-old Swedes: a contribution to the nordic diet discussion. Eur J Nutr. 2018 Dec;57(8):2869–76.

36. Ding M, Li J, Qi L, Ellervik C, Zhang X, Manson JE, et al. Associations of dairy intake with risk of mortality in women and men: three prospective cohort studies. BMJ. 2019 Nov 27;367:l6204.

37. Mills PK, Annegers JF, Phillips RL. Animal product consumption and subsequent fatal breast cancer risk among seventh-day adventists. Am J Epidemiol. 1988 Mar;127(3):440–53.

38. Katagiri R, Sawada N, Goto A, Yamaji T, Iwasaki M, Noda M, et al. Association of soy and fermented soy product intake with total and cause specific mortality: prospective cohort study. BMJ. 2020 Jan 29;368:m34.

39. Hirayama T. Relationship of soybean paste soup intake to gastric cancer risk. Nutr Cancer. 1981 Jan;3(4):223–33.

40. Ngoan LT, Mizoue T, Fujino Y, Tokui N, Yoshimura T. Dietary factors and stomach cancer mortality. Br J Cancer. 2002 July;87(1):37–42.

41. Kurozawa Y, Ogimoto I, Shibata A, Nose T, Yoshimura T, Suzuki H, et al. Dietary habits and risk of death due to hepatocellular carcinoma in a large scale cohort study in Japan. Univariate analysis of JACC study data. Kurume Med J. 2004;51(2):141–9.

42. Rebello SA, Koh H, Chen C, Naidoo N, Odegaard AO, Koh WP, et al. Amount, type, and sources of carbohydrates in relation to ischemic heart disease mortality in a Chinese population: a prospective cohort study. Am J Clin Nutr. 2014 July;100(1):53–64.

43. Wada K, Oba S, Nagata C. Rice-based diet and cardiovascular disease mortality in Japan: from the Takayama study. Nutrients. 2022 May 30;14(11):2291.

44. Chow WH, Schuman LM, McLaughlin JK, Bjelke E, Gridley G, Wacholder S, et al. A cohort study of tobacco use, diet, occupation, and lung cancer mortality. Cancer Causes & Control. 1992;3(3):247–54.

45. Paganini-Hill A, Kawas CH, Corrada MM. Non-alcoholic beverage and caffeine consumption and mortality: the Leisure World cohort study. Preventive Medicine. 2007 Apr;44(4):305–10.

46. Zhong GC, Hu TY, Yang PF, Peng Y, Wu JJ, Sun WP, et al. Chocolate consumption and all-cause and cause-specific mortality in a US population: a post hoc analysis of the PLCO cancer screening trial. Aging. 2021 July 31;13(14):18564–85.

47. Zhao B, Gan L, Yu K, Männistö S, Huang J, Albanes D. Relationship between chocolate consumption and overall and cause-specific mortality, systematic review and updated meta-analysis. Eur J Epidemiol. 2022 Apr;37(4):321–33.

48. Sun Y, Liu B, Snetselaar LG, Wallace RB, Shadyab AH, Chen GC, et al. Chocolate consumption in relation to all-cause and cause-specific mortality in women: the women’s health initiative. JAND. 2023 June;123(6):902-911.e3.

49. Buijsse B, Feskens EJM, Kok FJ, Kromhout D. Cocoa intake, blood pressure, and cardiovascular mortality. Arch Intern Med. 2006;166:411–7.

50. Kwok CS, Boekholdt SM, Lentjes MAH, Loke YK, Luben RN, Yeong JK, et al. Habitual chocolate consumption and risk of cardiovascular disease among healthy men and women. Heart. 2015 Aug 15;101(16):1279–87.

51. Ho YL, Nguyen XMT, Yan JQ, Vassy JL, Gagnon DR, Gaziano JM, et al. Chocolate consumption and risk of coronary artery disease: the Million Veteran Program. Am J Clin Nutr. 2021 May;113(5):1137–44.
